# Mitochondrial complex I bridges a connection between regulation of carbon flexibility and gastrointestinal commensalism in the human fungal pathogen *Candida albicans*

**DOI:** 10.1371/journal.ppat.1006414

**Published:** 2017-06-01

**Authors:** Xinhua Huang, Xiaoqing Chen, Yongmin He, Xiaoyu Yu, Shanshan Li, Ning Gao, Lida Niu, Yinhe Mao, Yuanyuan Wang, Xianwei Wu, Wenjuan Wu, Jianhua Wu, Dongsheng Zhou, Xiangjiang Zhan, Changbin Chen

**Affiliations:** 1 Unit of Pathogenic Fungal Infection & Host Immunity, CAS Key Laboratory of Molecular Virology & Immunology, Institut Pasteur of Shanghai, Chinese Academy of Sciences, Shanghai, China; 2 College of Life Science, Shanghai University, Shanghai, China; 3 Department of Laboratory Medicine, Shanghai East Hospital, Tongji University School of medicine, Shanghai, China; 4 College of Life Science, Shanghai Normal University, Shanghai, China; 5 Department of Dermatology, Shanghai Changhai Hospital, Second Military Medical University, Shanghai, China; 6 State Key Laboratory of Pathogen and Biosecurity, Beijing Institute of Microbiology and Epidemiology, Beijing, China; 7 Key Laboratory of Animal Ecology and Conservation Biology, Institute of Zoology, Chinese Academy of Sciences, Beijing, China; University of Toronto, CANADA

## Abstract

Efficient assimilation of alternative carbon sources in glucose-limited host niches is critical for colonization of *Candida albicans*, a commensal yeast that frequently causes opportunistic infection in human. *C*. *albicans* evolved mechanistically to regulate alternative carbon assimilation for the promotion of fungal growth and commensalism in mammalian hosts. However, this highly adaptive mechanism that *C*. *albicans* employs to cope with alternative carbon assimilation has yet to be clearly understood. Here we identified a novel role of *C*. *albicans* mitochondrial complex I (CI) in regulating assimilation of alternative carbon sources such as mannitol. Our data demonstrate that CI dysfunction by deleting the subunit Nuo2 decreases the level of NAD^+^, downregulates the NAD^+^-dependent mannitol dehydrogenase activity, and consequently inhibits hyphal growth and biofilm formation in conditions when the carbon source is mannitol, but not fermentative sugars like glucose. Mannitol-dependent morphogenesis is controlled by a ROS-induced signaling pathway involving Hog1 activation and Brg1 repression. *In vivo* studies show that *nuo2*Δ/Δ mutant cells are severely compromised in gastrointestinal colonization and the defect can be rescued by a glucose-rich diet. Thus, our findings unravel a mechanism by which *C*. *albicans* regulates carbon flexibility and commensalism. Alternative carbon assimilation might represent a fitness advantage for commensal fungi in successful colonization of host niches.

## Author summary

Most fermentative sugars like glucose, although routinely used in laboratory cell culture medium, are in fact only present at very low levels and even absent in many host niches. Therefore, assimilation of alternative nutrients is essential for the survival, proliferation and infection of most clinically important microbial pathogens like *C*. *albicans* in their hosts. In this study, we show that mitochondrial complex I (CI) is indispensable for proper hyphal growth and biofilm formation of *C*. *albicans* cells when mannitol, but not fermentative sugars like glucose or mannose, is used as the sole carbon source. We also find that a specific signaling pathway that senses and responds to the alternative carbon source incorporates input from mitochondrially-derived molecules like reactive oxygen species (ROS) to influence activation of the Hog1 MAPK and expression of the biofilm-regulator Brg1. Our findings further demonstrate that CI dysfunction confers a severe defect of *C*. *albicans* in gastrointestinal colonization and changing the diet with glucose is able to significantly rescue the commensal defect. Our study suggests that *C*. *albicans* has a unique regulatory system to sense and utilize the alternative carbon sources abundant in the GI tract and to promote commensalism. Significantly, CI activity appears to play a vital role in this highly adaptive system to regulate commensalism, in addition to its well-characterized role in virulence.

## Introduction

*Candida albicans* is by far the most prevalent commensal and pathogenic *Candida* species. In mammals, this polymorphic fungus most commonly resides as a lifelong, harmless commensal on mucosal surfaces of the oropharynx, gastrointestinal and genitourinary tracts in 30–70% of healthy individuals [1–3]. The mucosa surface provides a natural barrier in preventing the invasion of *C*. *albicans*. Successful colonization by the fungus requires a homeostasis between *C*. *albicans* and host immunity [4]. Once the balance is disrupted, e.g., unbalanced microbial flora after antibiotic treatment, weakened host immune response or impaired proliferation of epithelial cells, *C*. *albicans* rapidly transits from being a commensal to a pathogen and therefore causes serious and life-threatening systemic infections [5].

For clinically important microbial pathogens like *C*. *albicans*, assimilation of locally available nutrients is important for their survival, proliferation and infection in the diverse host niches. Interestingly, nutrients available in the host are mostly different from the ones supplied in the laboratory culture medium. Most fermentative sugars including glucose, fructose and galactose, although routinely used in laboratory cell culture medium, are actually only present at very low levels and even absent in many host niches. For example, compared to 2% glucose often used for culturing *C*. *albicans in vitro*, only 4–7 mM (0.07–0.13%) glucose exists in the bloodstream and its concentration in vaginal fluids is about 28 mM (0.5%) [6, 7]. Whereas in human intestinal tract, glucose concentrations are thought to be extremely low because glucose derived either from hydrolysis of starch or from sucrose is rapidly taken up into the epithelial cells by glucose transporters [8]. Therefore, *C*. *albicans* colonization in these glucose-poor niches must rely on alternative, non-fermentative carbon sources. For example, physiologically relevant carbon sources, including amino acids, fatty acids, carboxylic acids, glycerol, mannitol and N-acetylglucosamine (GlcNAc), are found at varying concentrations in different host niches and constitute major relevant nutrients for *C*. *albicans* upon infection. A classical example supporting the considerable metabolic flexibility of *C*. *albicans* is the glyoxylate cycle, a metabolic pathway that permits the use of two-carbon compounds as carbon sources and is required for *C*. *albicans* virulence in the mouse model of systemic candidiasis [9]. Disruption of the GlcNAc catabolic pathway significantly causes morphological changes and attenuated virulence in *C*. *albicans* [10]. Lactate is the carboxylic acid highly enriched in the gastrointestinal tracts [11]. Inhibition of lactate assimilation by deleting *CYB2*, a gene encoding L-lactate dehydrogenase, compromises the ability of *Candida glabrata* to colonize in the GI tract [12]. Moreover, lactate influences *C*. *albicans* recognition and phagocytosis by immune cells [13]. These studies attest to the distinct effect of alternative carbon assimilation on the host-pathogen interaction, particularly its contribution to virulence and commensalism. As previously stated, mechanisms that *C*. *albicans* employs to cope with alternative carbon assimilation are largely unknown. It remains unclear whether the regulation of carbon flexibility contributes to colonization of *C*. *albicans* in different host niches.

Mitochondria are the organelles generating most energy supply for the eukaryotic cells, converting oxygen and nutrients into the coenzyme adenosine triphosphate (ATP), which is primarily synthesized by the process of oxidative phosphorylation (OXPHOS) and the OXPHOS-electron transport chain (ETC). The ETC harbors five integral protein complexes in the inner mitochondrial membrane: the first four transfer high-energy electrons from NADH to molecular oxygen, and potential energy is established by a proton gradient and finally dissipated through complex V to synthesize ATP [14]. Among these complexes, mitochondrial complex I (CI), also referred to as NADH:ubiquinone oxidoreductase, is the first enzyme of ETC catalyzing NADH oxidation by regenerating NAD^+^ [15]. In addition to the primary role in ATP generation from nutrients, CI has other important cellular functions. For example, CI activity has been found to be important in regulating the yeast-to-hypha morphogenesis and pathogenicity of *C*. *albicans*. *NDH51* encodes the NADH dehydrogenase protein of CI and deletion of *NDH51* in *C*. *albicans* results in a filamentation defect even at low level of glucose [16]. Growth and Oxidation Adaption protein 1 (Goa1) is a mitochondrial protein uniquely present in the CTG subclade of the *Saccharomycotina* including *C*. *albicans*, deletion of *GOA1* in *C*. *albicans* significantly impairs the enzymatic function of CI [17] and therefore exhibits increased reactive oxygen species (ROS) production and cell death, heightened sensitivity to oxidants and neutrophil killing, and avirulent in a murine systemic infection model [18]. Similarly, deletion of either *NUO1* or *NUO2*, each encoding a subunit of NADH:ubiquinone oxidoreductase, causes CI disassembly and a number of physiological defects including reduced oxygen consumption, decreased mitochondrial redox potential, decreased CI activity, increased ROS and decreased chronological aging *in vitro*, as well as significantly attenuated virulence in a murine systemic infection model [19, 20]. Importantly, a link between the mitochondrial activity and intracellular signals appears to exist and decides the fate of cellular morphology in *C*. *albicans*. Post-transcriptional regulation by the RNA binding protein Puf3 and the mRNA deadenylase Ccr4 is crucial for mitochondrial biogenesis in *C*. *albicans* biofilms and this regulatory mechanism links metabolic adaptation to biofilm maturation [21]. Environmental factors such as methylene blue (MB) treatment interfere with mitochondrial activity and consequently down-regulate ATP production, which significantly decreases signaling through the Ras1-cAMP-PKA pathway and inhibits the Ras1-dependent yeast-to-hypha switch [22]. Of significance to our study, these data reflect a fact that most of the identified mitochondria-associated cellular events appear to rely on carbon sources. Indeed, searching for genes essential for utilization of GlcNAc identified a mitochondrial protein Mcu1 and deletion of *MCU1* leads to defects in utilizing a variety of non-fermentative carbon sources such as GlcNAc, sodium citrate, sodium pyruvate, acetate, ethanol and glycerol [23]. CI dysfunction by either *NUO1* or *NUO2* deletion results in severe growth defects in conditions when glycerol or oleic acid is used as the sole carbon source. Given that alternative carbon sources, rather than fermentative sugars, are highly enriched in mammalian host niches such as GI tract [24], these studies strongly suggest that mitochondrial activity may actively promote *C*. *albicans* colonization in host niches by regulating alternative carbon assimilation. However, the exact regulatory mechanisms, especially signaling pathways that incorporate input from mitochondria to sense and respond to alternative carbon assimilation, have not been fully elucidated.

In this study, we identified an uncharacterized carbon source-dependent function of CI in regulating hyphal morphogenesis and biofilm development of *C*. *albicans*. Both *in vitro* and *in vivo* studies demonstrate that CI triggers a specific, carbon source-dependent signaling pathway to allow *C*. *albicans* to adjust to disparate environments with varied availability of carbon sources, highlighting its role in modulating morphological changes of *C*. *albicans* by manipulating its flexibility to respond to local changes in intracellular environment and metabolism.

## Results

### Mitochondrial complex I (CI) is essential for mannitol-induced biofilm formation in *C*. *albicans*

**Fig 1:**
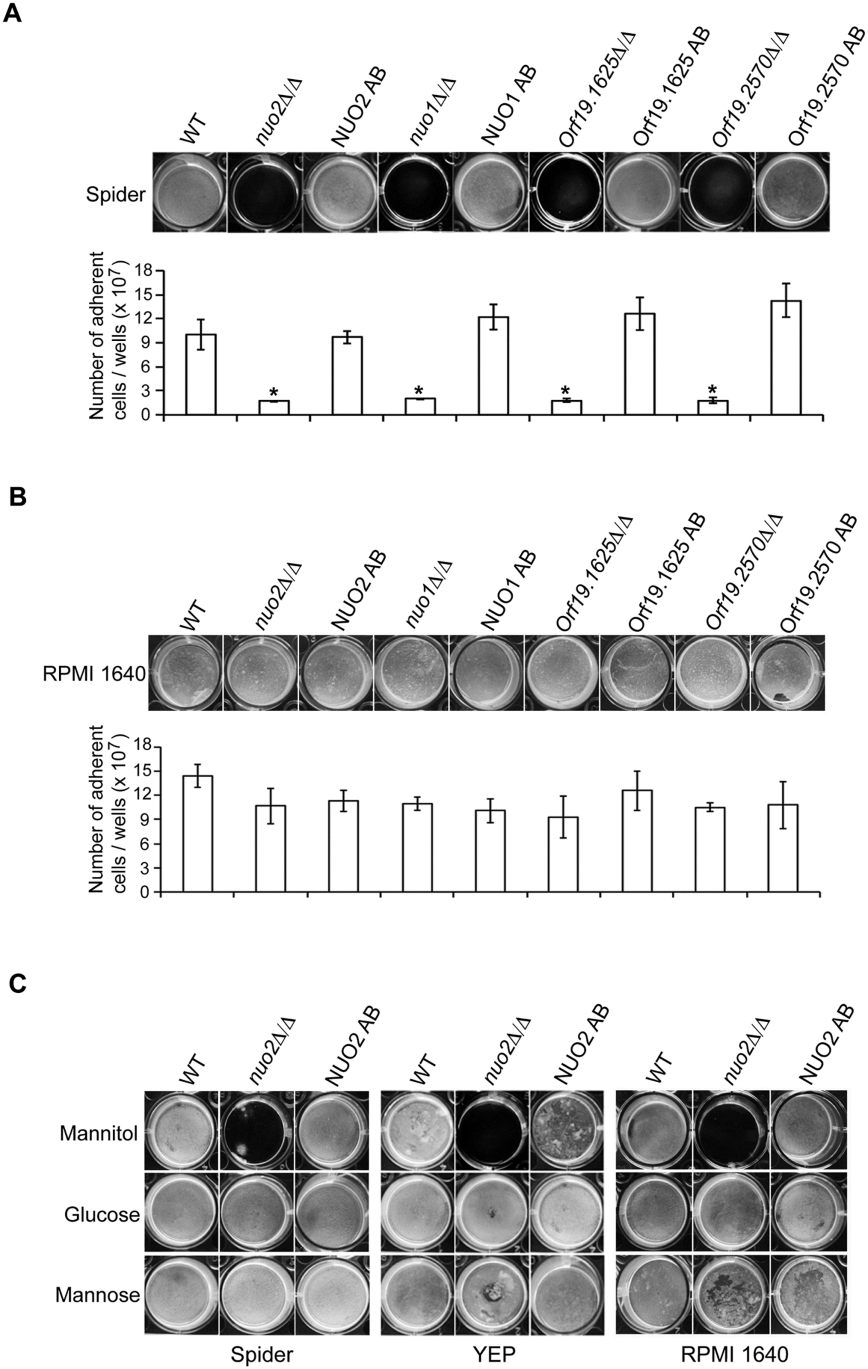
CI is required for mannitol-induced biofilm formation in *C*. *albicans*. (A) *in vitro* biofilm formation on Spider medium. Cultures of wild type *C*. *albians*, the CI subunit (Nuo2, Nuo1, orf19.1625 and orf19.2570) mutant strains, and their respective complemented derivatives, were grown in biofilm mode on 12-well plates for 48 h. The visual appearance of biofilms was shown and samples were further analyzed by the number of adherent cells. Four CI mutants were significantly reduced in biofilm formation in Spider medium using mannitol as the sole carbon source (“*”represents *P*<0.001 for WT vs. mutant). Values are the mean ± SD from two independent experiments with at least three replicates. AB indicates heterogeneous addback of one copy of the deleted gene to the corresponding mutant. (B) *in vitro* biofilm formation on RPMI 1640 medium. Cultures were treated in exactly the same way as described in (A), with the exception that growth medium was changed from Spider to RPMI 1640. (C) Requirement of the CI subunit Nuo2 in biofilm formation depends on available carbon sources. Shown is visual appearance of biofilms when cultures of wild type, *nuo2*Δ/Δ or NUO2 AB strain were grown in YEP, Spider or RPMI 1640 medium supplemented with different carbon sources.

A standard set of biofilm-inducing condition is to expose the *C*. *albicans* cells on either the silicone square substrate or directly on the bottom of 12-well bovine serum-recoated polystyrene plates, and incubate for 24 to 48 hours in Spider media, with gentle shaking of samples at 37°C [25]. Using this method, we screened the homozygous gene deletion library [26] for mutants defective in biofilm formation. Our screen identified a list of important regulators (S4 Table). Among them, four genes (*NUO1/Orf19*.*6607*, *NUO2/Orf19*.*287*, *Orf19*.*1625*, and *Orf19*.*2570*), each encoding the subunit of mitochondrial complex I (CI), were chosen for further analysis. Fig 1A showed that deletion of any one of the four CI subunits severely abolished biofilm formation. We did not observe biofilm formation in these mutants even when the incubation time was extended up to 14 days (S1A Fig). The biofilm defects of these mutants were also confirmed by dry weight measurements (S1B and S1C Fig). Reintroducing an ectopic copy of the wild type allele back into each mutant rescued the biofilm defect of each mutant, indicating that the phenotype is due to the deleted genes. Hence, all of the four CI subunits are required for wild type biofilm formation *in vitro*, at least under this set of biofilm-inducing condition.

To further ascertain the importance of these CI subunits in biofilm formation, we subjected the same set of strains to a different type of biofilm assay, where we kept all manipulations unchanged except that the cells were incubated in RPMI 1640, a medium previously known to promote biofilm formation in *C*. *albicans* [27]. To our surprise, we found that in RPMI 1640 medium, these four mutants formed biofilm that were indistinguishable from the wild type (Fig 1B), suggesting that nutrients differed in Spider and RPMI 1640 media may account for the opposite biofilm phenotypes. Nutritional comparison of these two media revealed differences in the source of carbon, that is, Spider medium uses mannitol while RPMI 1640 uses glucose as the sole carbon source. This led us to hypothesize that source of carbon might be contributing to the nature of biofilm development observed in these CI mutants. Therefore, we evaluated biofilm formation of these CI mutants in a carbon source-switching assay by adding one of three carbon sources, including glucose, mannitol and mannose, to different biofilm-inducing media. The reason we use mannose in this assay is because mannitol degradation produces D-fructose-6-phosphate via a conversion of mannitol to mannose and mannose is converted to mannose-6-phosphate which can enter the glycolysis pathway (Details can be found in *Candida genome database*). Because all four identified genes belong to the same family of CI subunits and each mutant displays a similar phenotype, we chose *NUO2* as a representative and carried out the rest of experiments. As shown in Fig 1C, *nuo2*Δ/Δ mutant displayed regular biofilm formation in Spider medium when mannitol was replaced by either glucose or mannose. In contrast, adding mannitol to glucose-free YEP or RPMI 1640 medium significantly compromised the biofilm growth of *nuo2*Δ/Δ mutant. Considering all these explicit results, we propose that a functional CI positively regulates biofilm development by imposing its effect on assimilation of mannitol, a sugar normally recognized as an alternative carbon source.

### Nuo2 is required for mannitol-induced hyphal formation in *C*. *albicans*

**Fig 2:**
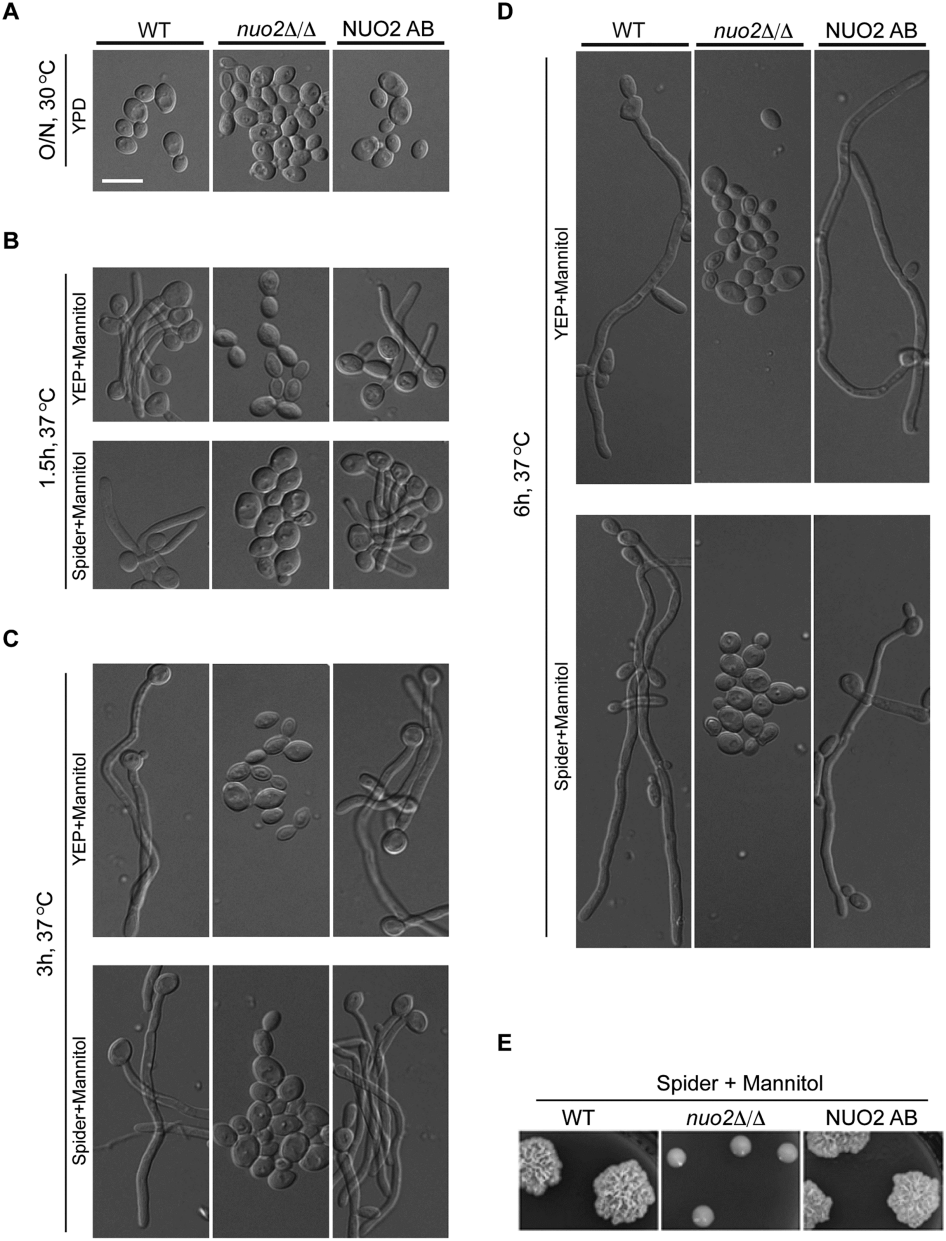
Nuo2 is required for mannitol-stimulated hyphal growth in *C*. *albicans*. (A to D) Cultures of wild type, *nuo2*Δ/Δ and NUO2 AB strains were grown overnight in a liquid YPD at 30°C, pelleted, washed in PBS, resuspended in an equal volume of PBS, and diluted 1:250 in either YEP or Spider medium supplemented with 2% of mannitol. Cells were continued to incubate at 37°C and hyphal morphologies were visualized under microscopy. Experiments were repeated in triplicates. Shown are representative images of *C*. *albicans* cells at initial overnight culture (A), 1.5 h (B), 3 h (C) and 6 h (D) after incubation in medium containing mannitol. Scale bars are 10μm. (E) Cells from Wild type, *nuo2*Δ/Δ and NUO2 AB strains were serially diluted and a representative dilution is displayed on Spider medium containing 2% mannitol at 37°C. Picture was taken after 2–3 days of growth.

Our findings that deleting *NUO2* impairs *C*. *albicans* biofilm development in a carbon source-dependent manner prompted us to investigate the role of this CI subunit in hyphal morphogenesis, given that hyphal development is an important step in normal biofilm development [28]. A standard hyphal induction assay was performed at 37°C in Spider or YEP medium supplemented with different carbon sources. Cells from wild type, *nuo2*Δ/Δ mutant and *nuo2*Δ/Δ+*NUO2* complemented strains (NUO2 AB) were harvested from overnight cultures (Fig 2A) and re-inoculated to indicated medium. Aliquots of the cells were microscopically visualized at different time points. Apparently, when mannitol was used as the sole carbon source, massive *C*. *albicans* hyphae were observed in wild type, whereas *nuo2*Δ/Δ mutant cells were completely devoid of hyphae and remained in yeast form (Fig 2B–2D). Intriguingly, hyphal inhibition was still evident in *nuo2*Δ/Δ cells even for a prolonged incubation (up to 14 days) (S2A Fig). Additionally, the hyphae-defective phenotype of *nuo2*Δ/Δ mutant can be recapitulated in solid media (Fig 2E and S2B Fig). This phenotype was complemented by re-introduction of the wild type *NUO2* gene into the mutant genome. By comparison, defective hyphal growth of *nuo2*Δ/Δ mutant could be partially rescued by replacement of carbon source with either glucose (S3 Fig) or mannose (S4 Fig), and its germination rate and the hyphal elongation were much slower than wild type strain. These results support the proposition that hyphal morphology can also be influenced by carbon source, and necessarily in relation to CI function.

A recent study done by She *et al*. [20] has validated Nuo2 as a NADH:ubiquinone oxidoreductase and annotated its gene function. Mutant cells lacking *NUO2* displayed reduced oxygen consumption, decreased mitochondrial redox potential, decreased CI activity, increased reactive oxidant species (ROS) and attenuated virulence in a murine systemic infection model. In line with these reported findings, we also observed a significant reduction of intracellular ATP levels in *nuo2*Δ/Δ mutant regardless of carbon sources, although the effect of mannitol appears to be more evident (S5 Fig). Taking into consideration the *in vitro* phenotypic characteristics of *nuo2*Δ/Δ mutant, our results suggest that CI dysfunction results in defective biofilm formation and hyphal growth in a carbon source-dependent manner, reflecting the possibility that the mutant cells may have an intrinsic inability to sense and utilize the alternative carbon sources such as mannitol.

### Inhibition of hyphal growth of *nuo2*Δ/Δ mutant is not majorly due to a general growth defect or potentially increased extracellular osmotic stress

**Fig 3:**
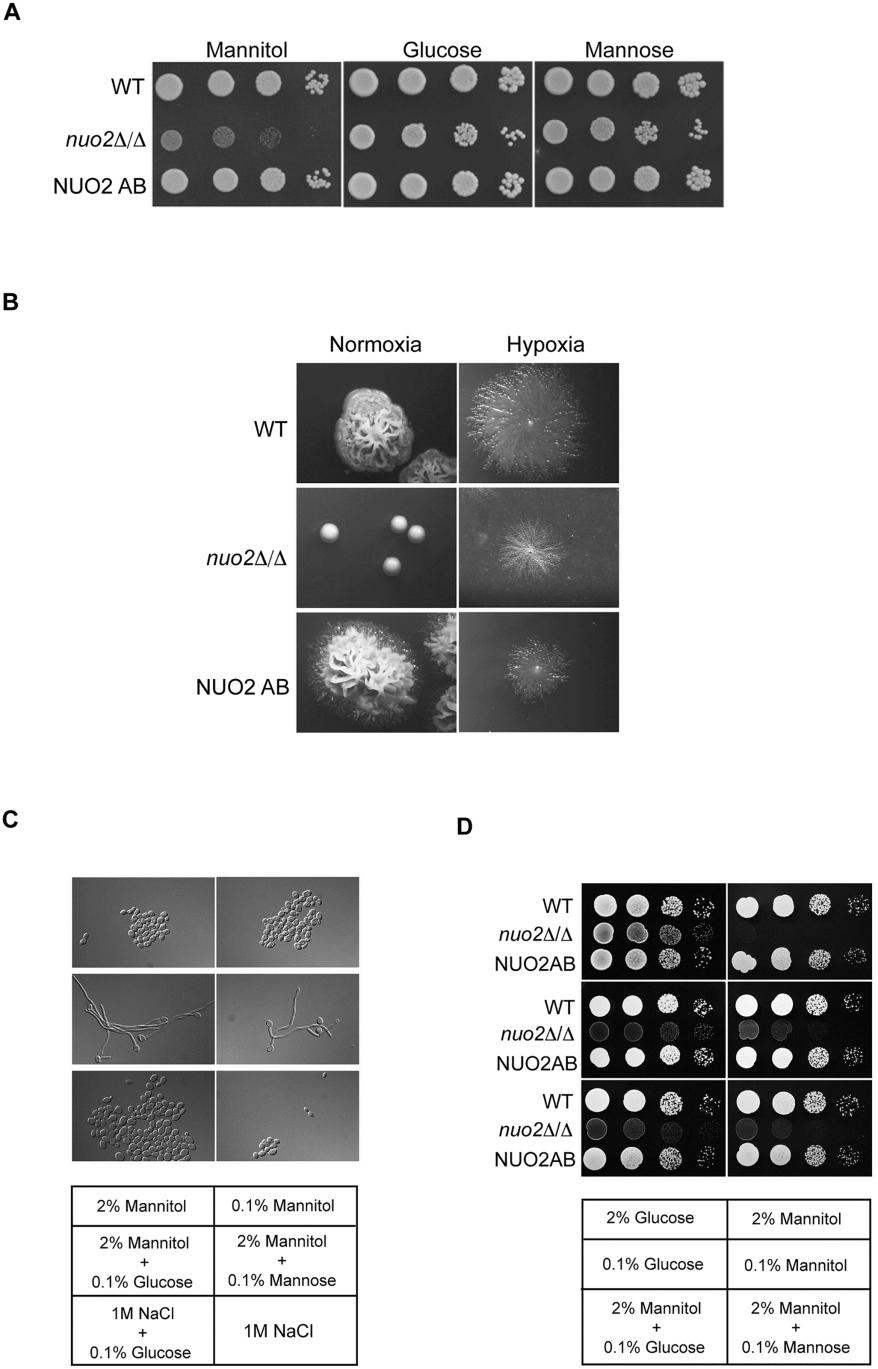
Mannitol-dependent hyphal inhibition in the *nuo2*Δ/Δ mutant is not majorly due to defective proliferation and increased osmotic potential. (A) Vegetative growth of Wild type, *nuo2*Δ/Δ and NUO2 AB strains on YEP medium supplemented with 2% of mannitol, glucose or mannose. Cells were grown to mid-log stage (8h of growth; OD_600_ = 1.0), serially diluted and displayed on plates. The colonies were photographed after incubation at 30°C for 3 days. (B) Hyphal comparison of indicated strains under normoxic and hypoxic conditions. Strains were initially plated for single colonies on Spider medium and incubated under normoxic or hypoxic (controlled CO_2_ incubator; less than 0.2% oxygen) condition in the dark at 37°C. Pictures were taken after 3 days of incubation under normoxic condition and 7 days under hypoxic condition, respectively. (C) Phenotypic assay of Nuo2 including morphology under conditions that YEP medium is supplemented with different combinations of carbon sources and salt. The *nuo2*Δ/Δ mutant cells were inoculated on YEP medium containing different concentrations of carbon sources in the presence or absence of salt (1M NaCl) and incubated at 37°C. Pictures were taken after 3 h of incubation. (D) Effect of glucose or mannose supplementation on vegetative growth of *nuo2*Δ/Δ mutant under mannitol condition. Cell cultures (pre-diluted to OD_600_ = 0.8) from wild type, *nuo2*Δ/Δ or NUO2 AB strains were diluted serially in 10 fold increments prior to being spotted onto YEP plates supplemented with different combinations of carbon sources. Plates were incubated at 30°C and pictures were taken after 2 days.

The role of mitochondrion as a power station in generating ATP undoubtedly could have important effects on cell growth. Indeed, severe growth defects were observed in *nuo2*Δ/Δ mutant when mannitol, rather than glucose or mannose, was used as the sole carbon source (Fig 3A and S6A–S6C Fig). Here, incubation on different carbon sources was at 30°C instead of 37°C. Notably, cells lacking *NUO2* still presented in the yeast form (Fig 2 and S7A Fig) after an increase in temperature, a condition that stimulates filamentous growth in wild type and complemented strains, and we found that the null mutant strain exhibited indistinguishable patterns of growth under both temperature conditions (S6 and S7B Figs). Therefore, the restrictions of assaying morphology and cell proliferation of *nuo2*Δ/Δ mutant at two different temperatures (37°C vs 30°C) could be neglected. Furthermore, our observation that *nuo2*Δ/Δ cells displayed severe growth defects in a mannitol-dependent manner raised an immediate question of whether inability of this mutant to trigger hyphal growth in mannitol-containing medium is due to a general, inherent defect in vegetative growth. To answer this, we assessed hyphal morphogenesis of the *nuo2*Δ/Δ cells under different conditions. Firstly, we examined its growth under hypoxia (0.2% O_2_), a condition that *C*. *albicans* cells grow exclusively as hyphae [29, 30]. Cells were inoculated on mannitol-containing YEP medium and incubated at 37°C for 7 days under normoxic and hypoxic conditions, respectively. We found that although *nuo2*Δ/Δ mutant cells grew even more slowly, hypoxia restored regular but shorter hyphae (Fig 3B, note that pictures were taken after 7 days incubation), indicating that lack of hyphae formation in the medium using mannitol as the sole carbon source is not due to a general growth defect of the *nuo2*Δ/Δ mutant. In contrast, normoxic condition failed to stimulate hyphae formation even the incubation time was extended up to 30 days (S2B Fig). Secondly, we evaluated hyphal morphology of *nuo2*Δ/Δ mutant when glucose was added to mannitol-containing medium. Cells were grown at 37°C in YEP medium supplemented with different combinations of carbon sources and hyphal morphologies were monitored under microscopy. Although hyphal formation was completely suppressed by adding 2% of mannitol to YEP medium, we found in Fig 3C that adding an extremely low concentration of glucose or mannose (0.1%) to the mannitol medium is sufficient to restore hyphal growth, however, the treatment only leads to a modest growth improvement, as mutant cells propagated more slowly than the wild type and NUO2 AB strains (Fig 3D and S6D–S6G Fig). These results further support the notion that filamentation defect of *nuo2*Δ/Δ mutant is independent from its growth defect, that is, impaired growth appears to play a minor role in hyphal suppression. In addition, a previous study in the *C*. *albicans* strain BWP17 showed that CI dysfunction by deleting the CI subunit-encoding gene *NDH51* caused a severe defect in filamentous growth at 37°C in the basal salts medium (BSM), a condition known to induce filamentation [16]. Moreover, treating the wild type cells with rotenone, a potent CI inhibitor that inhibits the transfer of electrons from Fe-S centers in CI to ubiquinone [31], significantly decreases filamentation without affecting the doubling times [32]. These results implied that the hyphal defect of *ndh51*Δ/Δ cells was due to inhibition of the respiratory pathway and not to impaired growth. Using a similar strategy, we evaluated the effect of rotenone on proliferation and hyphal morphology of wild type (SN250) cells. After exposure to rotenone for 6h, cells proliferated at almost the same rate as untreated (control) (S8A Fig), but the rate of filamentation was significantly decreased to only 20% (S8B Fig; note that 95% of cells undergo filamentous growth in the absence of rotenone). Further filamentation decreases occurred at later time points (S8B Fig) but cell proliferation was only mildly slowed down (S8A Fig). These results match the observed decrease in the *NUO2* transcript level after rotenone treatment (S8C Fig). Our data, therefore, provide indirect evidence that hyphal inhibition by CI dysfunction could not be ascribed to impaired growth. However, in BSM, we observed that CI dysfunction by deleting *NUO2* substantially blocked hyphal development but only marginally impaired the cell proliferation (S8D Fig), implying that the unknown composition of the medium may somehow compensate for the mannitol-dependent growth defect in *nuo2*Δ/Δ mutant. Taken together, our data argue against the notion that a defect in growth is the predominant mechanism by which CI dysfunction blocks hyphal development in a carbon source-dependent manner. It is likely that defects in hyphal formation and proliferation may be uncoupled when CI mutant cells are exposed to alternative carbon sources such as mannitol.

Previous studies have established that the addition of mannitol to the culture medium could impose effects on cells by either the osmotic effect [33] or the role of mannitol as a carbon source [34]. Because the *nuo2*Δ/Δ mutant cells failed to efficiently utilize mannitol present in the medium, we next ask whether an osmotic effect due to extracellular accumulation of unassimilated mannitol could contribute to the defective hyphal growth of this mutant. Given that glucose has been found to repress the uptake of mannitol in *C*. *albicans* [35], we monitored hyphal growth of *nuo2*Δ/Δ cells in conditions when 0.1% glucose was added to YEP medium containing either 2% mannitol or a high concentration of salt (1M NaCl), an osmotic condition that has been reported to completely inhibit the switch of *C*. *albicans* yeast cells to hyphal form [36]. As shown in Fig 3C, we observed regular hyphal growth after 0.1% glucose was added to the medium containing mannitol but not salt, arguing against a role for mannitol-induced osmotic stress in this phenotype.

Therefore, it is more evident that CI functions to mediate mannitol-induced biofilm development and hyphal growth by regulating its assimilation and an uncharacterized signal transduction pathway might be involved.

### Nuo2 is required for mannitol-induced enzymatic activity of the NAD^+^-dependent mannitol dehydrogenase

**Fig 4:**
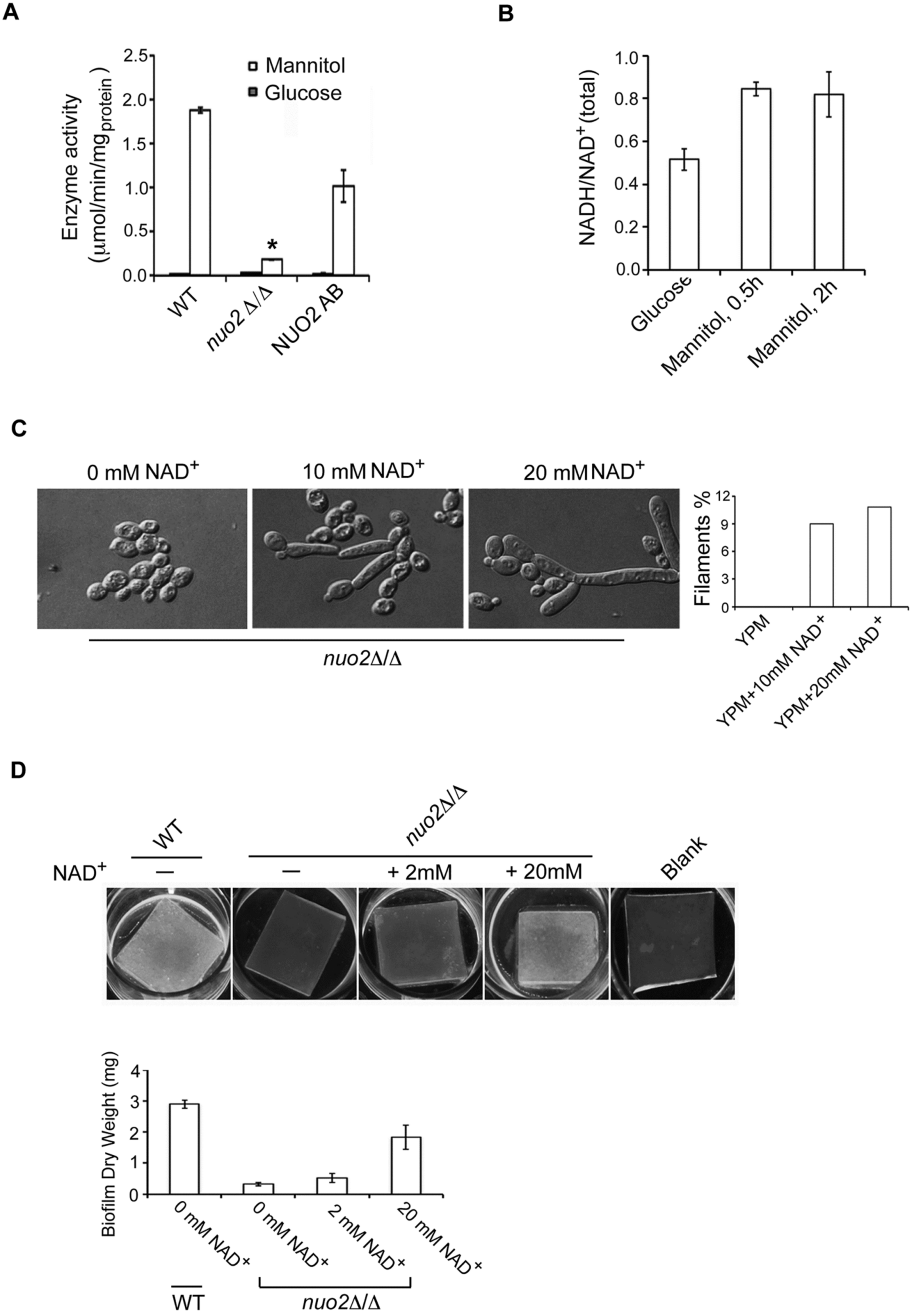
Glycolytic inhibition by decreasing availability of NAD^+^ contributes to defective hyphae and biofilm formation of *nuo2*Δ/Δ cells under mannitol condition. (A) Mannitol dehydrogenase activity in whole-cell extracts derived from Wild type, *nuo2*Δ/Δ and NUO2 AB cells. Cells were grown at 37°C in 50ml of YPD (YEP + 2% of glucose) or YPM (YEP + 2% of mannitol) for 2 days and cell lysates were used to assay the enzyme activity. “*”represents *P*<0.001 for WT vs. mutant. The results are the means of at least three independent experiments, and error bars show mean values and SD. (B) Treating the *nuo2*Δ/Δ cells with mannitol significantly increases the NADH/NAD^+^ ratio. Mutant cells were originally incubated in YPD medium and then shift to the same volume of YPM, and continued to incubate at 37°C. Cell lysates were prepared before or after mannitol treatment and NAD^+^ and NADH contents were measured. The results are the means of at least three independent experiments, and error bars represent the SDs. (C) NAD^+^ supplementation increases filamentation of *nuo2*Δ/Δ mutant in mannitol-containing medium. An overnight culture of *nuo2*Δ/Δ mutant were diluted and inoculated in mannitol-containing YEP medium in the presence or absence of 10mM or 20mM NAD^+^. Cells were continued to incubate at 37°C for 4h and hyphal morphologies were visualized under microscopy. Experiments were repeated in triplicates. Shown are representative images of *C*. *albicans* cells after 4h incubation. Quantification of filamentation in 100 cells in each experiment is shown on the right. (D) NAD^+^ supplementation is able to restore biofilm formation of *nuo2*Δ/Δ mutant in mannitol-containing medium. As in C, cells from wild type or *nuo2*Δ/Δ strain were grown as biofilms in mannitol-containing YEP medium supplemented with different doses of NAD^+^. Samples were analyzed by cell adhesion on plate with silicone square and biofilm dry weights. Values are the mean ± SD from two independent experiments with at least three replicates.

Mannitol metabolism requires an enzyme named mannitol dehydrogenase which mediates the conversion of mannitol to mannose by catalyzing the chemical reaction: Mannitol + NAD^+^—Mannose + NADH + H^+^ [37]. Mannitol dehydrogenase belongs to the family of NAD^+^-dependent oxidoreductases that specifically act on the CH-OH group of donor with NAD^+^ as acceptor [38]. Since replacing mannitol with mannose is able to restore the hyphal growth and biofilm formation of *nuo2*Δ/Δ mutant, we hypothesized that CI dysfunction may have an impact on enzymatic activity of the mannitol dehydrogenase. Cells from wild type, *nuo2*Δ/Δ, and NUO2 AB strains were cultured in YEP medium supplemented with either glucose or mannitol, and mannitol dehydrogenase activity was determined by an assay previously described in *Sacchromyces cerevisiae* [39]. As expected, mannitol, but not glucose, triggers a significant increase of enzymatic activity in the wild type and complemented strains (Fig 4A). However, mannitol-induced enzymatic activity was significantly diminished in the *nuo2*Δ/Δ mutant, indicating that Nuo2 is required for upregulation of mannitol dehydrogenase activity. Considering the fact that *NUO2* encodes a subunit of NADH:ubiquinone oxidoreductase catalyzing the production of NAD^+^ from NADH and enzymatic activation of mannitol dehydrogenase is NAD^+^-dependent, we hypothesized that downregulation of mannitol dehydrogenase activity in *nuo2*Δ/Δ mutant could be due to a reduction of NAD^+^ level. To test this, we measured the ratio of cellular NADH/NAD^+^ following a carbon source shift. Cells were originally grown in the presence of glucose and then shift to a fresh medium using mannitol as the sole carbon source. As expected, deletion of *NUO2* led to about 40% reduction of NAD^+^ level (Fig 4B). Thus, our results clearly indicate that CI dysfunction by deleting *NUO2* significantly reduces the overall level of NAD^+^ and thereby downregulates the activity of NAD^+^-dependent mannitol dehydrogenase, a key enzyme responsible for mannitol metabolism. As a result, mannitol cannot be converted to mannose and glycolytic pathway through mannose metabolism is blocked. To test whether the defects in hyphal morphology and biofilm formation in *nuo2*Δ/Δ were due to reduced NAD^+^ levels, we examined morphological changes of *nuo2*Δ/Δ cells in mannitol-containing YEP medium supplemented with different concentrations of NAD^+^. Strikingly, NAD^+^ supplementation is able to rescue the defects in hyphae and biofilm formation (Fig 4C and 4D), supporting that glycolytic inhibition by decreasing availability of NAD^+^ contributes to impaired hyphae and biofilm formation of *nuo2*Δ/Δ cells in mannitol condition.

### Increased ROS in *nuo2*Δ/Δ mutant specifically activates the Hog1 MAPK pathway

**Fig 5:**
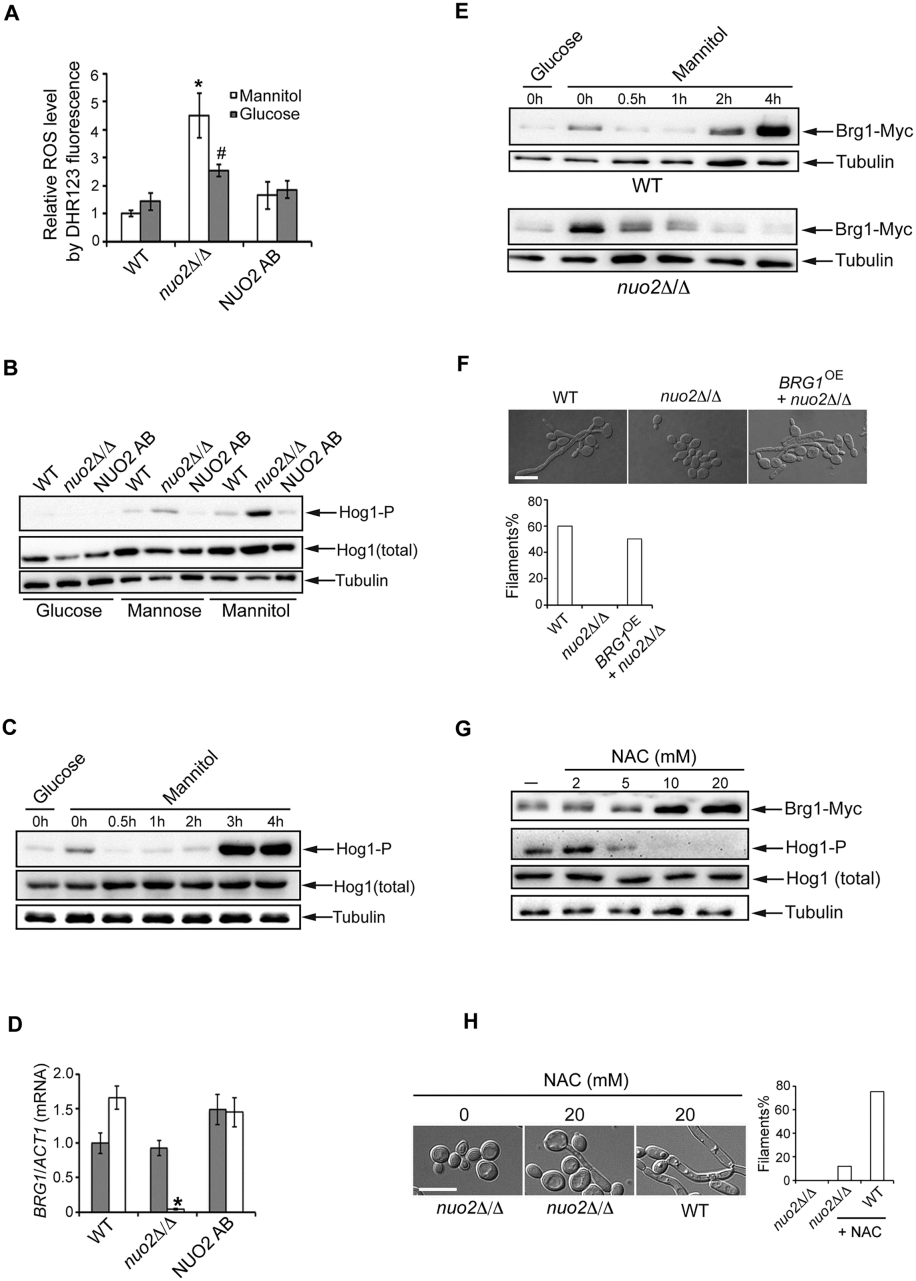
Deletion of *NUO2* triggers a ROS-dependent signaling pathway involving Hog1 activation and Brg1 repression in conditions when mannitol is the sole carbon source. (A) Measurement of ROS generation using a dihydrorhodamine 123 (DHR123) staining method in WT, *nuo2*Δ/Δ and NUO2 AB cells after incubation on glucose or mannitol-containing medium. Exponential-phase cells (6h of growth; OD_600_ = 0.5) derived from indicated strains were harvested and ROS production was determined by DHR123 fluorescence. For each strain, three independent biological replicates were used. “*” represents *P*<0.001 for WT vs. mutant under mannitol condition and “^#^” represents *P*<0.05 for WT vs. mutant under glucose condtion. The results represent the means of three independent experiments, and error bars represent the SDs. (B) Deletion of *NUO2* triggers a mannitol-dependent activation of Hog1. An overnight culture of each of indicated strains was diluted at 1:250 into YPD medium. Log phase cultures (6h; OD_600_ = 0.5) were collected, washed, and replaced with equal volume of YEP containing 2% of glucose, mannitol or mannose, and continued to grow at 37°C for 3 h. Protein extracts were fractionated by SDS-PAGE and immunoblotted with antibodies against either the phosphorylated or total Hog1. Immunoblotting with antibodies against the α-tubulin was used to control for variation in loading. (C) Hog1 is highly activated in *nuo2*Δ/Δ mutant following a carbon source-shifting assay. Log-phase mutant cells, originally grown in YPD medium, were collected, washed and re-inoculated to equal volume of YPM medium. Cells were continued to grow at 37°C and collected at indicated time intervals for Western analysis. Protein extracts were treated exactly the same as 5B. (D) Deletion of *NUO2* specifically downregulates *BRG1* expression in a mannitol-dependent manner. Cells from WT, *nuo2*Δ/Δ and NUO2 AB strains were grown in YPM medium to mid-log stage. Relative transcript levels of *BRG1*, a gene encoding one of the six core biofilm transcriptional regulators, were assessed by quantitative PCR (qPCR). Values obtained for each gene were normalized against *ACT1* for each sample to give relative expression. “*”represents *P*<0.001 for WT vs. mutant. Error bars represent standard deviation of three independent biological replicates. (E) Mannitol-induced Hog1 activation correlates with dampened Brg1 expression in *nuo2*Δ/Δ mutant. Cells from either Wild type (top panel) or *nuo2*Δ/Δ strain (bottom panel), each expressing genetically C-terminal tagged Brg1-Myc, were grown in YPD (YEP + 2% of glucose) medium to reach a log-phase (6h at 30°C; OD_600_ = 0.5). After wash at least three times in prewarmed PBS, cells were reinoculated to equal volume of YPM (YEP + 2% of mannitol; prewarmed to 37°C) medium, continued to grow at 37°C and collected at indicated time points for Western analysis, using antibodies against Myc epitope and α-tubulin (loading control). (F) Overexpression of *BRG1* in *nuo2*Δ/Δ mutant is sufficient to restore hyphal foramtion. Cells of wild type, *nuo2*Δ/Δ and *BRG1* overexpressed (*BRG1*^OE^ +*nuo2*Δ/Δ) strains were grown at 37°C for 2h in YPM. Hyphal morphologies were visualized under microscopy. Quantification of filamentation in 100 cells in each experiment is shown. Scale bar, 10μm. (G) A signaling pathway showing an inverse correlation between Hog1 activation and Brg1 expression specifically participates in the response to ROS. Log-phase of the *nuo2*Δ/Δ mutant cells grown in YPM were treated with or without the antioxidant N-Acetyl Cysteine (NAC) at different doses for 5 hours. Protein extracts were assayed by sequential immunoblotting with antibodies against Myc epitope, phosphorylated Hog1, total Hog1 and α-tubulin (loading control). (H) Treating the *nuo2*Δ/Δ mutant cells with NAC partially restores the hyphal growth in RPMI medium using mannitol (2%) as the sole carbon source. Overnight cultures of wild type and *nuo2*Δ/Δ mutant were diluted and inoculated in RPMI 1640 medium supplemented with 2% mannitol, in the presence or absence of NAC (20mM). Cells were continued to incubate at 37°C and hyphal morphologies were visualized under microscopy. Experiments were repeated in triplicates. Shown are representative images of *C*. *albicans* cells after 24h incubation. Quantification of filamentation in 100 cells in each experiment is shown on the right. Scale bar, 10μm.

It has been documented that CI inhibition impedes the reoxidation of NADH and thus free electrons react with ambient oxygen to produce reactive oxygen species (ROS) [40, 41]. Previous studies in *C*. *albicans* showed that treatment with rotenone, a specific CI inhibitor, significantly induces ROS generation [42]. In line with these observations, we and *She et al*. [20] also confirmed that deletion of *NUO2* results in significant accumulation of ROS in glucose condition (Fig 5A). Remarkably, mannitol treatment further increased the already elevated ROS levels (Fig 5A and S9 Fig; note that similar results were obtained using two different ROS detection methods), suggesting that glucose-triggered ROS production might be insufficient to impose a harmful effect on hyphal morphology of *nuo2*Δ/Δ cells, instead, defective hyphal growth necessitates accumulation of ROS to higher levels in the presence of mannitol. Given that ROS are no longer recognized as just a toxic by-product of mitochondrial respiration, but are now appreciated for their roles as important and common secondary messengers that are poised at the core of signaling pathways maintaining normal metabolic fluxes and different cellular functions [43, 44], we speculated that an uncharacterized ROS-mediated signaling pathway may contribute to the carbon source-dependent phenotypes observed in CI mutants. We considered a possibility of the classic Hog1 MAPK signaling cascade since this pathway has been found to play a crucial role in sensing and responding to oxidative stress, and in repressing hyphal formation in *C*. *albicans* [45, 46]. To test this, we performed two independent immunoblotting analyses by detecting activation of Hog1 under different growth conditions. After cells from WT, *nuo2*Δ/Δ and NUO2 AB strains were grown to exponential phase in YEP medium supplemented with different carbon sources, cell extracts were prepared and subjected to immunoblotting analysis, using an antibody that specifically recognizes the phosphorylated form of Hog1 since Hog1 is activated in response to cationic stress via phosphorylation at its conserved TGY motif [46]. Compared to the wild type and complemented strains, we observed significantly higher levels of phosphorylated Hog1 in the *nuo2*Δ/Δ mutant cells under the condition when mannitol, rather than glucose or mannose, was the sole carbon source (Fig 5B). As controls, analysis of Hog1 in wild type and mutant strains showed that the total Hog1 protein levels did not change upon treatment with different carbon sources. We further investigated the time course of Hog1 phosphorylation in both wild type and *nuo2*Δ/Δ mutant cells after shifting the carbon source from glucose to mannitol, glucose to glucose or glucose to mannose. Among these conditions, we found that a strong induction of phosphorylated Hog1 was only observed in the *nuo2*Δ/Δ cells after a glucose to mannitol shift (Fig 5C and S10 Fig). Taken together, our data demonstrate that CI dysfunction stimulates a signal from ROS to flow through the Hog1 MAPK system in a mannitol-dependent manner.

### An inverse correlation between Hog1 activation and Brg1 expression in *nuo2*Δ/Δ mutant

Hyphal formation is an important feature of biofilms as mutants deficient in hyphal growth are often impaired in biofilm development [47]. Because Hog1 activation has been previously documented to inhibit the yeast-to-hypha switch via the Sko1-dependent repression of *BRG1* expression [46], we hypothesized that the transcription factor Brg1 might act downstream of Hog1 signaling to mediate inhibition of hyphae and biofilm formation in *nuo2*Δ/Δ mutant when mannitol was used as the sole carbon source. To test this hypothesis, we first measured transcript levels of *BRG1* responding to varied carbon sources (Fig 5D). In the glucose condition, expression of *BRG1* remained at low levels and displayed no significant differences among the wild type, *nuo2*Δ/Δ mutant and NUO2AB strains. In marked contrast, its expression was abolished in the *nuo2*Δ/Δ mutant when mannitol was used as the sole carbon source, suggesting that impaired hyphae and biofilm formation of *nuo2*Δ/Δ cells could be due to mannitol-dependent *BRG1* downregulation. Second, inhibitory effect of CI dysfunction on *BRG1* expression was further evaluated by measuring its protein levels. An epitope-tagged version of Brg1 was utilized in which 13 copies of the Myc epitope were fused in-frame at the C-terminus and this fusion protein is fully functional since recombinant strain expressing Brg1-Myc had no effect on the yeast-to-hypha transition (S11 Fig). A glucose-mannitol shifting assay was carried out and we found that although the steady state level of Brg1-Myc in wild type was substantially induced by mannitol (Fig 5E, top panel), Brg1 induction was significantly abolished by deleting *NUO2* (Fig 5E, bottom panel). These results demonstrate that mannitol-dependent morphological defects in *nuo2*Δ/Δ involves decreased levels of Brg1. Neverthless, a recent study showed that Brg1, together with other 5 transcription regulators (Bcr1, Efg1, Ndt80, Rob1 and Tec1), constitutes a core transcriptional network that regulates biofilm formation and lack of any one of these regulators significantly blocked biofilm *in vitro* [25]. This prompted us to investigate whether CI dysfunction by deleting *NUO2* could specifically impact *BRG1* expression or have a general role in affecting expression of all genes in the network. Transcript levels of genes encoding the remaining 5 transcriptional regulators, including *BCR1*, *EFG1*, *NDT80*, *ROB1* and *TEC1*, were quantified by RT-qPCR. Our results indicated that unlike *BRG1*, the five genes had similar expression patterns between the wild type and *nuo2*Δ/Δ mutant in a carbon source-independent manner (S12 Fig), highlightling a specific role of Brg1 in CI-regulated, mannitol-dependent hyphal growth and biofilm development in *C*. *albicans*. Interestingly, a puzzling pattern from Western blots was observed in the two samples labeled as time point 0: a rapid induction of Brg1-Myc during the shift from glucose to mannitol (Fig 5E). A possibility we considered is that the irregular induction might be due to the samples cultured at different temperatures, given the fact that the glucose sample at time point 0 was cultured at 30°C, whereas the mannitol sample at time point 0 was actually maintained at 37°C after a carbon source shift. As for a higher level of Brg1 in the mutant than in the wild type at time point 0, a possible explanation could be that CI dysfunction may confer increased susceptibility to a rapid temperature change. A previous study has shown that a temperature increase in wild type *C*. *albicans* leads to decreased level of phosphorylated Hog1 and we therefore speculate that this effect might be strengthened by CI dysfunction, which could induce a higher level of Brg1, given an inverse relationship between Hog1 phosphorylation and Brg1 expression.

The above results had indicated a mannitol-dependent Brg1 downregulation as a consequence of CI dysfunction. To further confirm this finding, we sought to overexpress *BRG1* in *nuo2*Δ/Δ cells with the prediction that *BRG1* overexpression could result in a rescue of hyphae and biofilm defects. The endogenous promoter of *BRG1* was replaced with a constitutively active *TDH3* promoter in *nuo2*Δ/Δ strain (*nuo2*Δ/Δ + *BRG1*^OE^). Increased level of *BRG1* mRNA was confirmed by RT-qPCR (S13 Fig). As expected, overexpressed *BRG1* substantially rescued the defects in hyphae and biofilm formation (Fig 5F and S14 Fig), supporting the idea that Brg1 plays a critical role in CI-mediated cellular activities that sense and utilize available carbon sources to promote hyphal growth and biofilm formation. Surprisingly, overexpression of *BRG1* was unable to rescue the growth defect of *nuo2*Δ/Δ mutant (S15 Fig) even though the failure of the mutant to form hyphae and biofilm could be recovered, providing additional evidence for our conclusion that the mannitol-dependent defects in hyphae and biofilm formation in *nuo2*Δ/Δ mutant are unlikely to be attributable to the impaired growth.

In view of our results, we next ask whether the signaling pathway involving Hog1 activation and Brg1 repression specifically participates in the response to ROS. To this end, we treated the *nuo2*Δ/Δ mutant cells with N-acetyl-L-cysteine (NAC), a potent antioxidant. Strikingly, we found that 10mM NAC was sufficient to restore high levels of Brg1 expression and coincidently diminished the level of phosphorylated Hog1 (Fig 5G). As a control, we observed that NAC treatment in the wild type cells led to downregulation of Brg1 expression in a dose-dependent manner, whereas it had no effect on the level of phosphorylated Hog1 (S16 Fig). To our surprise, we found that NAC treatment only partially complemented the hyphae and biofilm defects of *nuo2*Δ/Δ cells (Fig 5H and S17 Fig), which could be due to the observation that although Brg1 was induced by NAC in a dose-dependent manner, its peak level in *nuo2*Δ/Δ mutant was still lower than that attained in untreated wild type (S18 Fig; compare lanes 9 and 10 to lane 1). Importantly, like the case with *BRG1* overexpression, NAC treatment also did not improve the growth of mutant cells (S19 Fig), further suggesting a disconnection between the morphological defect and impaired growth in *nuo2*Δ/Δ mutant under the mannitol condition. Interestingly, NAC treatment seems to have a negative impact on wild type biofilm formation (S17 Fig), possibly due to downregulation of Brg1 expression (S16 Fig).

Taken together, these results establish that CI dysfunction reduces the level of NAD^+^ and blocks the glycolytic pathway by downregulating enzymatic activity of the mannitol dehydrogenase, that *NUO2* disruption impedes the reoxidation of NADH and thus free electrons react with ambient oxygen to produce ROS, and that inhibition of hyphal growth and biofilm development occurs through a signal from ROS to flow through a specific, mannitol-dependent signaling pathway involving Hog1 activation and Brg1 repression. Intriguingly, the glycolytic inhibition appears to act independently from the ROS signaling as treating the *nuo2*Δ/Δ cells with NAD^+^ had no effect on Brg1 expression (S20 Fig). It is more likely that exogenous NAD^+^ bypasses the inhibitory effect of CI dysfunction on the activity of the NAD^+^-dependent mannitol dehydrogenase and thus enables the cells to enter the glycolysis, a pathway which contributes to hyphal growth and biofilm formation through an as yet uncharacterized, Brg1-independent mechanism.

### Nuo2 is required for mannitol-induced, Brg1-regulated expression of hyphae-specific genes

Brg1 promotes hyphae-specific gene expression by directly binding to hyphal gene promoters and sustains hyphae elongation [46, 48]. Since Brg1 expression was repressed when *nuo2*Δ/Δ mutant cells were treated with mannitol, we determined whether, under these conditions, Brg1 repression specifically had an effect on expression of hyphae-specific genes (HSGs). Cells from the wild type, *nuo2*Δ/Δ mutant and NUO2 AB strains were grown at 37°C in YEP medium supplemented with either glucose or mannitol and quantitative expression of HSGs were measured by RT-qPCR. Compared to the condition that glucose was the sole carbon source, mannitol strongly induced expression of three well-characterized HSGs (*ECE1*, *HWP1* and *ALS3*) in the wild type but the induction was abolished in the *nuo2*Δ/Δ mutant (S21A Fig). Interestingly, it appears that CI dysfunction does not influence the expression of all HSGs because *HYR1* shows similar levels of expression in both wild type and *nuo2*Δ/Δ mutant. In a similar manner we analyzed the expression of these four genes in *nuo2*Δ/Δ mutant treated with or without NAC (S21B Fig). After exposed to NAC, the *HWP1* transcript was largely increased, a slight but significant upregulation of *ECE1* was detected, but transcript levels of *HYR1* and *ALS3* remained unchanged. Importantly, their transcript expression modes nicely fit the morphological pattern showing a partial restoration of hyphae and biofilm formation after a NAC treatment. Thus, the results reinforce an essential role of a functional CI in regulating mannitol-induced hyphal growth and biofilm formation of *C*. *albicans*.

### CI activity is associated with murine gastrointestinal colonization of *C*. *albicans*

**Fig 6:**
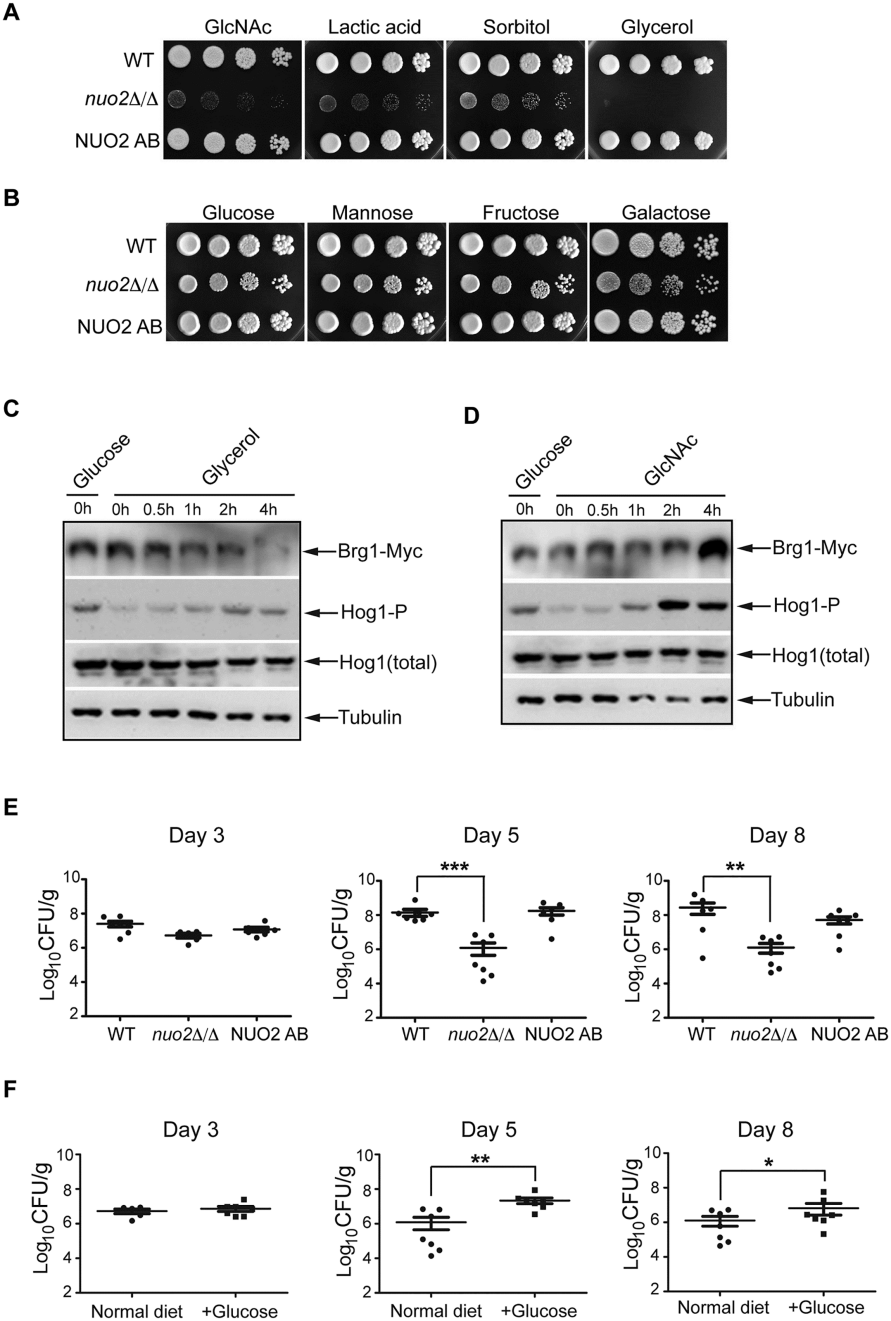
CI-regulated utilization of alternative carbon sources is vital for *C*. *albicans* commensalism in GI tract. (A) The *nuo2*Δ/Δ mutant cells displayed *in vitro* defects in proliferation in medium supplemented with different alternative carbon sources. Cultures diluted to OD_600_ = 0.8 were diluted serially in 10 fold increments prior to being spotted onto YEP plates supplemented with GlcNAc, lactic acid, sorbitol or glycerol, each at 2%. Plates were incubated at 30°C and pictures were taken after 3 days. (B) Growth of wild type, *nuo2*Δ/Δ and NUO2 AB strains in medium supplemented with varied fermentative carbon sources. Cultures of each strain were treated in a way similar to (A). (C) Similar to mannitol, glycerol treatment also yields an inverse correlation between Hog1 activation and Brg1 expression in *nuo2*Δ/Δ mutant. Log-phase of *nuo2*Δ/Δ cells were inoculated to YEP medium supplemented with 2% glycerol. Cells were continued to grow at 37°C and collected at indicated time points for Western analysis, using antibodies against Myc epitope, phosphorylated Hog1, total Hog1 and α-tubulin (loading control). (D) Unlike mannitol and glycerol, GlcNAc treatment leads to induction of both Hog1 and Brg1 in *nuo2*Δ/Δmutant. Cells were treated in exactly the same way as described in (C), with the exception that 1% GlcNAc were used. (E) *C*. *albicans nuo2*Δ/Δ mutant strain displays defect in gastrointestinal colonization compared to wild type or NUO2 AB strains. Cells of wild type, *nuo2*Δ/Δ or NUO2 AB strains were separately inoculated into 7–10 female BALB/c mice by oral gavage (10^8^ CFU/mouse). Colonization was measured in fresh fecal pellets on day 3, 5 and 8 post-inoculation. *C*. *albicans* CFU/g of material was determined. Each symbol represents a sample from an individual mouse; bars indicate geometric means. Data from three independent experiments is shown. Asterisks indicate that *nuo2*Δ/Δ mutant colonization is lower than either WT or complemented strain: *, p≤0.05 (pairwise t-test, Bonferroni correction). (F) Diet change significantly improves colonization of *nuo2*Δ/Δ mutant strain in GI tract. Female BALB/c mice were divided into two groups (each has 7–10 mice): one group was fed the regular drinking water, a second group was instead fed drinking water containing 5% glucose. In a similar way as described in E, equal number of *nuo2*Δ/Δ cells were orally inoculated into each group of mice (10^8^ CFU/mouse) and gastrointestinal colonization was measured in fresh fecal pellets on day 3, 5 and 8 post-inoculation. Asterisks indicate that adding glucose to the diet significantly improves the colonization of *nuo2*Δ/Δ mutant in GI tract: *, p≤0.05 (pairwise t-test, Bonferroni correction).

*C*. *albicans* thrives as a commensal in the gastrointestinal (GI) tract, a niche with lower proportion of energy-rich carbon sources (e.g. glucose, fructose and galactose), indicating that its colonization in GI tract must rely on alternative carbon sources such as glycerol, GlcNAc, lactic acid and mannitol. Moreover, this fungus uses transcriptional programs associated with the yeast-to-hypha transition to maintain attachment, to invade tissue and to promote survival and immune evasion, as well as to regulate the behavior during GI tract colonization [49, 50]. Our *in vitro* results suggest that CI dysfunction severely disrupts mannitol assimilation and consequently blocks hyphal morphogenesis and biofilm development. Therefore, we decided to address the role of CI in GI colonization. For this purpose, we first analyze if CI activity holds a general feature of affecting utilization of alternative carbon sources. To this end, we compared the *in vitro* proliferation rates of the wild type, *nuo2*Δ/Δ mutant and NUO2 AB strains by growing them on YEP medium supplemented with either fermentative or alternative carbon sources. As shown in Fig 6A, growth defects of the *nuo2*Δ/Δ mutant responding to a variety of alternative carbon sources (GlcNAc, lactic acid, sorbitol or glycerol) were almost as strong as the defect shown by the mutant to mannitol. This observation is in contrast to the phenotype displayed by the *nuo2*Δ/Δ mutant responding to fermentative sugars such as glucose, mannose and fructose, which significantly compromise the growth defects (Fig 6B).

In order to testify whether the CI-dependent Hog1 pathway has a universal role in response to alternative carbon sources, we examined morphogenesis of the wild type and *nuo2*Δ/Δ cells in conditions when the sole carbon source is either glycerol or GlcNAc, considering their abundance in GI tract. Apparently, both glycerol and GlcNAc induce robust filaments in the wild type (S22A Fig). However, opposite effects were observed for the *nuo2*Δ/Δ mutant, that is, filamentation was blocked in glycerol but maintained in GlcNAc (S22B Fig). To determine if these distinct morphological changes in response to the two carbon sources reflect their different impacts on activation of Hog1 signaling, we analyzed the expression levels of Hog1 and Brg1 in *nuo2*Δ/Δ mutant after a carbon source shift from glucose to glycerol or GlcNAc. Indeed, we note that similar to mannitol, treating the *nuo2*Δ/Δ cells with glycerol also leads to an inverse correlation between Hog1 and Brg1 expression, as illustrated by the pattern that increased levels of phosphorylated Hog1 correlates with the spontaneous reduction of Brg1 expression during the course of glycerol (Fig 6C). This appears to be specific for the mutant cells since we observed a different pattern in the wild type showing that a glycerol shift induced robust Brg1 expression while the level of phosphorylated Hog1 was almost undetected (S23A Fig). Interestingly, unlike the effects of mannitol and glycerol on this signaling pathway, we found that under GlcNAc, the total amount of Brg1 and of phosphorylated Hog1 was strongly increased in both wild type and *nuo2*Δ/Δ mutant cells (Fig 6D and S23B Fig), suggesting that CI activity appears to have no role in sensing and utilizing this alternative carbon source. Furthermore, it is very likely that in addition to Brg1, other transcription factor(s) may operate to sense signal through Hog1, depending on which carbon source is available.

Our *in vitro* data highlight the importance of CI activity in promoting hyphal growth and biofilm development by regulating utilization of alternative carbon sources known to be highly enriched in GI tract. Since defects of hyphal growth, biofilm formation and utilization of alternative carbon sources were observed in the *nuo2*Δ/Δ mutant, we ask whether these defects could have profound effects on the ability of *C*. *albicans* cells to function as a commensal in a mammalian host. To this end, we assessed the influence of CI dysfunction on *in vivo* colonization of *C*. *albicans* in a mouse model of gastrointestinal infection. Following a protocol described previously [51], we inoculated 7–10 antibiotic-treated BALB/c mice by oral gavage with 1:1 mixtures of wild type and *nuo2*Δ/Δ mutant (1x10^8^ CFU/mouse). Fecal pellets were collected at specified intervals and plated on Sabouraud Dextrose Agar (SDA) medium. Because the *nuo2*Δ/Δ mutant displays a severe growth delay than the wild type, we can easily distinguish these two types of cells based on the size of colonies. A representative image that shows *C*. *albicans* cell colonies recovered from fecal samples at day 5 post-inoculation is displayed in S24 Fig. To our surprise, we found that the plate is fully occupied by wild type cells with only few small colonies representing the *nuo2*Δ/Δ mutant cells (S24 Fig, typified with arrows). Genetic backgrounds of these tiny colonies were further confirmed by PCR. However, it turns out to be problematic for the next step to measure the abundance of each strain in the innoculum and after recovery from fecal pellets by real-time PCR. Using primers specifically designed for identifying mutant cells, we did not detect any PCR signals when genomic DNAs derived from recovered cells were used as template. The most parsimonious explanation for our observed results is that wild type cells completely outcompete the *nuo2*Δ/Δ mutant in GI tract; however, we cannot exclude another possibility that competitive advantage of the wild type cells over mutant occurs only after the mixed cells from fecal pellets are patched on SDA plate. It is likely that overgrowth of the wild type on SDA plates significantly inhibits proliferation of the *nuo2*Δ/Δ mutant cells since our *in vitro* studies indicate that the mutant has severe growth defect and grows more slowly than the wild type (Fig 3A and S6 Fig). The results obtained from this mixed infection assay may not truly reflect the competitive relationship between wild type and the *nuo2*Δ/Δ mutant in GI tract.

To assess the role of CI in commensalism more accurately, we decided to inoculate mice with a single strain other than a 1:1 mixture. Cells of WT, *nuo2*Δ/Δ mutant or NUO2 AB strain were inoculated by oral gavage into the antibiotic-treated BALB/c mice (1x10^8^ CFU/mouse). Each strain shown here was evaluated in 7–10 mice, experiments were repeated twice independently. GI tract colonization was measured in fresh fecal pellets on days 3, 5 and 8 post-inoculation. On day 3 post-inoculation, the *nuo2*Δ/Δ mutant colonization was moderately lower than either wild type or NUO2 AB. The colonization defects of *nuo2*Δ/Δ mutant were even more significant on day 5 and 8 post-inoculation (Fig 6E). Thus, these results suggest that Nuo2 subunit is implicated in the murine gastrointestinal colonization by *C*. *ablicans*, possibly through its important roles in regulating utilization of alternative carbon sources which are abundant in GI tract. To further verify that CI-regulated carbon flexibility is the key factor contributing to *C*. *albicans* gut commensalism, we re-test the ability of *nuo2*Δ/Δ mutant to colonize the GI tract by changing the diet. We added 5% glucose to the drinking water together with antibiotics and persistently fed the mice throughout the experiment. Colonization of the *nuo2*Δ/Δ mutant in GI tract was monitored on day 3, 5 and 8 post-inoculation. We found that colonization defects of the *nuo2*Δ/Δ mutant were substantially rescued by glucose treatment (Fig 6F). In contrast, a glucose diet did not influence the gastrointestinal colonization of the wild type *C*. *albican* cells (S25 Fig). Therefore, these results highlight the importance of CI activity in metabolism being crucial to compete in the GI tract and CI dysfunction entails a disadvantage that affects the capability of *C*. *albicans* to efficiently assimilate alternative carbon sources which are highly enriched in this niche. Collectively, our data suggest that CI activity integrates its roles in sensing and utilizing available carbon sources to promote hyphal growth and biofilm formation, a process that may contribute to *C*. *albicans* commensalism in the GI tract.

## Discussion

In this paper, we use a combination of *in vitro* and *in vivo* approaches to provide the first description of the mechanism underlying regulation of alternative carbon assimilation in affecting *C*. *albicans* hyphal morphology, biofilm development and intestinal commensalism. Our data establish that mitochondiral complex I (CI) targets mannitol utilization by influencing the mannitol dehydrogenase activity and is essential for mannitol-induced hyphal growth and biofilm formation in *C*. *albicans*. We provide experimental evidence that the mannitol-dependent morphological defects upon CI dysfunction are uncoupled from impaired growth and may instead involve two distinct mechanisms. Finally, we demonstrate that CI dysfunction confers a severe defect of *C*. *albicans* in gastrointestinal colonization. By dissecting the involvement of CI in regulating *C*. *albicans* hyphal morphology, biofilm development and intestinal colonization, we unraveled a novel function of CI in bridging the connection between regulation of alternative carbon assimilation and *C*. *albicans* gastrointestinal commensalism.

### CI dysfunction affects hyphal growth and biofilm development of *C*. *albicans* through two independent mechanisms

**Fig 7:**
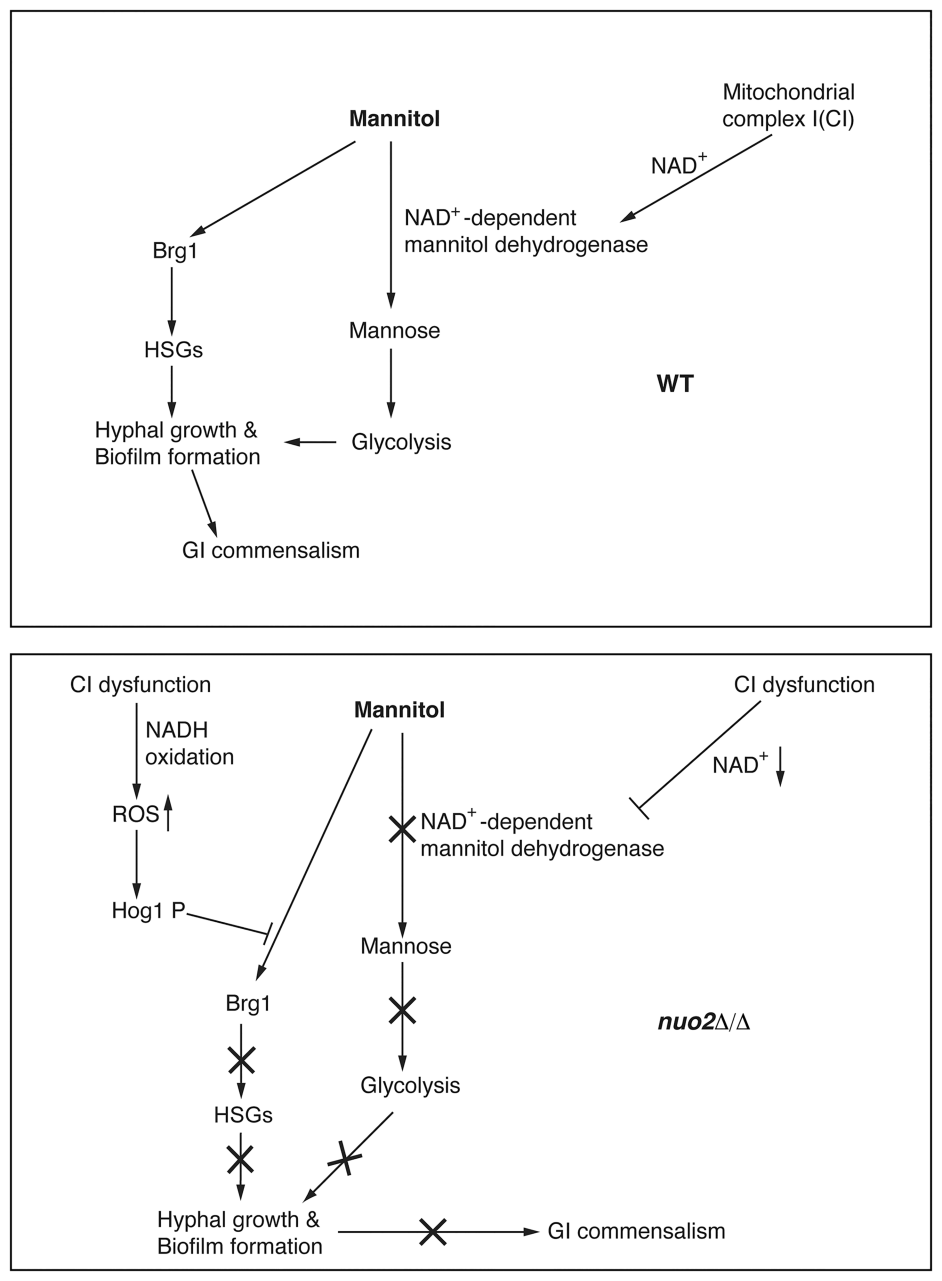
Model for the role of CI activity in mannitol-dependent fungal morphogenesis and gastrointestinal commensalism. Wild type *C*. *albicans* enters the glycolysis pathway by converting mannitol to mannose, a step catalyzed by the NAD^+^-dependent mannitol dehydrogenase whose activity is sustained by CI via producing NAD^+^. CI dysfunction by deleting *NUO2* inhibits hyphal growth, biofilm formation and gastrointestinal commensalism through at least two distinct mechanisms. For the first mechanism, deletion of *NUO2* blocks the glycolytic pathway by decreasing availability of NAD^+^ and therefore inhibits hyphae and biofilm formation through an as yet uncharacterized way. For the second mechanism, cells lacking *NUO2* produces high levels of ROS which triggers a mannitol-dependent signaling pathway involving Hog1 MAPK activation and Brg1 repression. In consequence, downregulation of HSGs inhibits the hyphal growth and biofilm development in a mannitol-dependent manner. By responding to nutrients available in gastrointestinal tract, *C*. *albicans* cells adjust CI activity to coordinate pathways required for hyphal morphogenesis and biofilm development that possibly promotes commensalism.

We propose a model (Fig 7) for the role of CI activity in promoting hyphal morphogenesis, biofilm formation and gastrointestinal commensalism in *C*. *albicans*. In conditions when the sole carbon source is mannitol, CI acts to provide sufficient levels of NAD^+^ to sustain the enzymatic activity of NAD^+^-dependent mannitol dehydrogenase and advance the glycolysis by converting mannitol to mannose. Accordingly, CI dysfunction by deleting *NUO2* significantly inhibits hyphae and biofilm formation in a mannitol-dependent manner, and the morphological defects are attributable to at least two distinct mechanisms. First, glycolytic inhibition by decreasing availability of NAD^+^ contributes to defective hyphae and biofilm formation of *nuo2*Δ/Δ cells because the defects can be rescued by NAD^+^ addition (Fig 4C–4E). Second, CI dysfunction triggers production of high levels of ROS which stimulates a mannitol-dependent signaling pathway involving Hog1 phosphorylation and the transcription factor Brg1 repression, leading to transcriptional downregulation of HSGs and subsequent inhibition of hyphal growth and biofilm development (Fig 5 and S21 Fig). Most importantly, glycolytic inhibition appear to be independent from activation of ROS signaling because NAD^+^ addition could rescue the morphological defects of *nuo2*Δ/Δ cells but had no effect on Brg1 expression (Fig 4 and S20 Fig). Given the impact of glycolysis on glycerol production and the regulatory role of glycerol on biofilm formation of *C*. *albicans* [52], it is likely that glycolytic inhibition in *nuo2*Δ/Δ mutant may affect the production of glycerol which has been known to play a regulatory role in biofilm formation by regulating expression of numerous biofilm-associated genes, in addition to its role in metabolism [52]. In addition, our observations that small amount of glucose or mannose seems to fully restore hyphal growth of *nuo2*Δ/Δ mutant imply the presence of the alternative mechanisms (Fig 3C), which warrant future studies.

In this work, we provide sufficient evidence that defects in filamentation and growth are uncoupled in the *nuo2*Δ/Δ mutant. Our data support the conclusion that a defect in growth may not be the predominant mechanism by which CI dysfunction blocks hyphae and biofilm formation in a mannitol-dependent manner. However, the contribution of growth defect could not be ruled out, but may play a relatively minor role in the morphological defects of *nuo2*Δ/Δ mutant. This conclusion was drawn upon two experimental points. First, treating the *nuo2*Δ/Δ cells with 0.1% glucose or mannose only partially improves its growth but almost fully restores hyphae formation (Fig 3D and S6D–S6G Fig). Second, the mutant cells exhibited shorter filaments than the wild type after 7 days under a hypoxic condition (Fig 3B), however, they remained in yeast form even after 30 days under a normoxic condition (S2 Fig).

Moreover, the observation that mannitol-dependent hyphal defect in *nuo2*Δ/Δ mutant could be rescued under hypoxia suggests the presence of a distinct signaling pathway that may override the effect of mannitol (Fig 3B). A recent study in *C*. *albicans* [29] has illustrated that under hypoxia, filamentous growth could be highly induced by activation of the Cek1 MAPK pathway and a number of transcription factors, including the activator Ace2 and two repressors like Efg1 and Bcr1, are involved. Interestingly, filamentation (37°C, hypoxia) requires Brg1 only in the absence of CO_2_; in its presence, Brg1 was dispensable.

### CI dysfunction increases intracellular ROS production and triggers a carbon source-dependent signaling pathway

Our analysis showed that a ROS-triggered signaling pathway, which involves Hog1 activation and Brg1 downregulation, contributes to the defective hyphae and biofilm formation of *nuo2*Δ/Δ mutant. More importantly, this ROS-responsive pathway seems to operate in a CI- and carbon source-dependent manner. For example, glycerol treatment also results in an inverse correlation between Hog1 activation and Brg1 repression in the *nuo2*Δ/Δ mutant but not wild type (Fig 6C and S23A Fig), a pattern that was characteristic of mannitol effects. In contrast, GlcNAc appears to act in a CI-independent way, as we observed simultaneous induction of Hog1 phosphorylation and Brg1 expression in both wild type and *nuo2*Δ/Δ mutant (Fig 6D and S23B Fig). These results suggest that additional, as yet uncharacterized, mechanisms may exist, depending on available carbon sources. We speculate that the differences could be due to the fact that the glycerol catabolic pathway in the yeast starts with the oxidation of glycerol to dihydroxyacetone (DHA) via a NAD^+^-dependent glycerol dehydrogenase [53], however, NAD^+^ appears to be dispensable for activities of GlcNAc metabolic enzymes [54].

An interesting question was raised as to why *nuo2*Δ/Δ cells trigger ROS accumulation when glucose is the sole carbon source [20], yet defects in hyphae and biofilm formation were not observed under such conditions? In order to tackle this question, we measured intracellular ROS production of different strains in conditions when glucose or mannitol was used as the sole carbon source. Although the *nuo2*Δ/Δ cells exhibited relatively higher levels of ROS than wild type in glucose condition, we surprisingly observed a continued increase of endogenous ROS after exposure to mannitol (Fig 5A, S9 Fig). Since *NUO2* encodes the subunit of NADH:ubiquinone oxidoreductase catalyzing the transfer of electrons from NADH to ubiquinone in a reaction involving proton translocation across the membrane [15], it is plausible that in the mannitol condition, loss of *NUO2* blocks glycolysis via downregulation of the NAD^+^-dependent mannitol dehydrogenase and thus might redirect the carbohydrate flux to an as yet unknown pathway resulting in a further accumulation of electrons that react with ambient oxygen to produce even higher levels of ROS [40,41].

### Carbon flexibility affects *C*. *albicans* gastrointestinal colonization

In addition to being the major systemic fungal pathogen of humans, *C*. *albicans* is predominantly considered as part of the commensal microbiome on the mucosal surfaces of the oral and vaginal cavities and gastrointestinal (GI) tract. Environmental cues, such as changes in carbon source, were found to be vital for the variety and dynamism of the niches that *C*. *albicans* inhabits in the human host niches [55]. It has been well documented that fermentative carbon hydrates such as glucose, fructose, or galactose, although are widely used in our *in vitro* assays for culturing fungal cells, are not available or present at low concentrations in host niches mentioned above [6, 9, 56]. Instead, alternative carbon sources, such as amino acids, glycerol, mannitol and organic acids, provide the main nutrients that support the *in vivo* growth of the infecting fungus. A previous study in humans has shown that 74% of mannitol passes through the small intestine and reaches the large intestine, where it is utilized by microbes like beneficial bacteria to produce organic acids, which can be used by the host [57]. Similarly, mannitol in rats was found to escape metabolism in the small intestine and reaches the cecum intact, where it is fermented by local microbes and promotes absorption of calcium and magnesium in the large intestine [58]. Therefore, metabolic flexibility of *C*. *albicans* reflects its ability to utilize a variety of different nutrients available in the diverse microenvironments of the host. If so, it makes sense that a successful colonization of *C*. *albicans* in GI tract may rely on its ability to assimilate locally available carbon sources such as mannitol. Given a severe defect of the *nuo2*Δ/Δ cells in assimilation of alternative carbon sources, it is not surprising that CI dysfunction may have profound effects on *C*. *albicans* commensalism in a mammalian host. Indeed, our commensalism experiments revealed that the *nuo2*Δ/Δ cells, but not wild type, conferred a significant defect in fitness in the mammalian GI tract (Fig 6E and S25 Fig). Remarkably, we note that this commensal defect can be rescued by changing a glucose-rich diet (Fig 6F). We conclude from this set of experiments that CI activity plays a major role in metabolism being crucial to compete in the GI tract and CI dysfunction entails a disadvantage that affects the capability of *C*. *albicans* to efficiently assimilate locally available nutrients, in particular, alternative carbon sources which are highly enriched and crucial for its colonization in this niche.

Although a growing body of literature has validated the necessity of regulation of alternative carbon source assimilation in promoting *C*. *albicans* pathogenicity [9, 22, 59], studies about its role in gastrointestinal commensalism are scarce. The results presented here, to our knowledge, for the first time unravel a mechanism underlying the interplay between CI-regulated utilization of alternative carbon sources and *C*. *albicans* commensalism. We propose that CI executes a carbon-dependent strategy to allow *C*. *albicans* to adjust to disparate environments with varied availability of carbon sources, highlighting its important role in bridging a connection between carbon flexibility regulation and gastrointestinal commensalism. Future work will aim to decipher the comprehensive picture of this signaling pathway responding to different alternative sources and if possible, to evaluate its impact on *C*. *albicans* colonization in other host niches such as skin and genital mucosa.

## Materials and methods

### Animals

Female BALB/c mice were purchased from Shanghai Laboratory Animal Center (Shanghai, China). Mice that were 6–8 weeks old and weighed 16-20g were used. These mice were maintained in a pathogen-free animal facility at Institut Pasteur of Shanghai. Infections were performed under SPF conditions. Animals were inoculated by oral gavage with the indicated dose of *C*. *albicans* cells in 100μl PBS buffer.

### Ethics statement

All animal experiments were carried out in strict accordance with the regulations in the Guide for the Care and Use of Laboratory Animals issued by the Ministry of Science and Technology of the People's Republic of China. All efforts were made to minimize suffering. The protocol was approved by IACUC at the Institut Pasteur of Shanghai, Chinese Academy of Sciences (Permit Number: A150291)

### Media and growth conditions

*C*. *albicans* strains were routinely propagated at 30°C in YPD. For growth phenotypes, yeast cells were grown at 37°C in YEP (1% yeast extract, 2% Facto-Peptone), Spider [60] or RPMI 1640 cell culture medium (Gibco corp.) supplemented with the appropriate carbon source including each of fermentable sugars (glucose, mannose, fructose and galactose) or alternative carbon sources (mannitol, GlcNAc, lactic acid, sorbitol, glycerol) at indicated concentrations. All carbons were purchased from Sigma-Aldrich. For carbon source-shift assays, logarithmically growing cells were diluted 1:250 in YPD medium and incubated at 37°C for 6 hours to reach an OD_600_ of 0.5, and sequentially washed twice with pre-warmed sterile water and reinoculated to equal volume of either YPD (glucose as carbon source) or YPM (mannitol as carbon source). Cells were continued to grow at 37°C and harvested for immunoblotting assays at indicated time points.

### Plasmids and strain construction

SC5314 genomic DNA was used as the template for all PCR amplifications of *C*. *albicans* genes. The *C*. *albicans* strains used in this study are listed in S1 Table. The primers used for PCR amplification are listed in S2 Table. Plasmids used for Brg1-Myc tagging and knockout gene complementation are listed in S3 Table. Construction of *C*. *albicans* knockout mutants, complemented strains, strains expressing Myc-tagged Brg1 fusion protein, and overexpression strain for *BRG1* was performed as previously described [51].

### *In vitro* biofilm assay

The *in vitro* biofilm growth assays were carried out using a previously established protocol with minor modifications [25]. In brief, overnight cultures of *C*. *albicans* strains were grown in YPD at 30°C. After wash twice with phosphate-buffered saline (PBS), the cells were diluted to an optical density at OD_600_ of 0.5 in 2ml of Spider medium. The 12-well polystyrene plate alone or 12-well plate with silicone square were previously treated with 10% fetal bovine serum overnight and washed with 2 ml of PBS. After inoculating each of indicated *C*. *albicans* strains, the plates were incubated at 37°C for 90 min at 100 rpm to allow initial adhesion of cells. Each well was washed once with 2ml of PBS to remove any nonadhering cells, and 2ml of fresh spider media was added to each well and biofilms were grown for another 48 h as described above. After removing the medium, each well was washed with 2ml of PBS, dried, and photographed or cells in the biofilm resuspended and quantified. For dry-mass measurements, the silicone squares were dried overnight and weighed the following day. The average total biomass of each strain was calculated from six independent samples after subtracting the mass of square with no cells added. Statistical significance (*P* values) was calculated using the Student’s two-tailed paired *t* test.

### Hyphal induction assays

Hyphal induction was manipulated using a standard method. Strains were grown overnight in a liquid YPD at 30°C, pelleted, washed twice in PBS, resuspended in an equal volume of PBS, and diluted 1:250 in different growth media. Cells were incubated at 37°C, harvested and hyphal morphologies were visualized under microscopy at indicated time points. For hyphal induction in hypoxia, experiments were conducted using Thermo Scientific Forma CO_2_ incubator (Thermo Scientific) by maintaining the oxygen concentration less than 0.2% O_2_. Following strain inoculation, the plate was placed into the chamber at 37°C immediately and photographed after 7 days.

### Quantitative real-time PCR (qPCR)

Saturated overnight cultures of wild type, *nuo2*Δ/Δ, *nuo2*Δ/Δ+*NUO2* complemented strains were inoculated into YPD to OD_600_ = 10^−4^ and incubated with shaking at 30°C. The next morning, logarithmically growing cells were diluted 1:250 in YPD medium and incubated at 30°C to reach an OD_600_ = ~0.4–0.5. Cells were collected, washed twice with pre-warmed water, resuspended in equal volume of freshly prepared YPD or YPM medium. After 2 h of incubation at 37°C, cells were harvested by centrifugation and methods for RNA isolation were carried out using a hot phenol method [61]. 1–2μg of each RNA was treated with RNase-free DNase I (Promega, Madison WI, USA) and reverse transcribed using the GoScript Reverse Transcription System (Promega, Madison WI, USA). qPCR was performed using the SYBR Green Master Mix (High ROX Premixed) (Vazyme, Nanjing, China) using the primers CBO278 and CBO279 for *BCR1*, CBO280 and CBO281 for *BRG1*, CBO282 and CBO283 for *EFG1*, CBO284 and CBO285 for *TEC1*, CBO286 and CBO287 for *NDT80*, CBO288 and CBO289 for *ROB1*, CBO290 and CBO291 for *HYR1*, CBO292 and CBO293 for *HWP1*, CBO294 and CBO295 for *ECE1*, CBO296 and CBO297 for *ALS3*. Normalization of expression levels was carried out using the *ACT1* genes and the primers for *ACT1* was used as previously described [61]. At least three biological replicates were performed per strain per condition.

### Protein extraction and western blotting

A previously described protocol was used to prepare *C*. *albicans* protein extracts [51]. Lysates corresponding to 1 OD_600_ of cells were analyzed by SDS-PAGE and immunoblotted with the antibodies including the anti-c-Myc (9E10, Covance Research) for Myc-tagged proteins, the anti-phospho-p38 MAPK (Thr180/Tyr182) (3D7) antibody 9215S (Cell Signaling, MA, USA) for phosphorylated Hog1 proteins, the anti-Hog1 (y-215) antibody sc-9079 (Santa Cruz Biotechnology, CA, USA) for total Hog1 proteins. Immunoblots were also probed with anti-α-tubulin antibody NB100-1639 (Novus Biologicals, CO, USA) as a loading control.

### Enzymatic activity assays

Cell extracts for *C*. *albicans* mannitol dehydrogenase activity assays were prepared using a protocol adapted from a method previously described in *S*. *cerevisiae* [39]. Briefly, cells of wild type, *nuo2*Δ/Δ and NUO2 AB strains were grown at 37°C in 50ml of YPD (YEP + 2% of glucose) or YPM (YEP + 2% of mannitol) for 2 days with continuous shaking at the speed of 95 rpm. After washed once with sterile water, half of the cells were resuspended in 750μl of 50 mM sodium phosphate (pH 6.5) and disrupted with acid-washed glass beads (Sigma-Aldrich) using a high-throughput tissue homogenizer scientz-192 (settings: 70MHZ/s, 8 x 30s with an 30s interval on ice). Cell lysates were centrifuged at 20,000x*g* for 10 min at 4°C and the supernatant was used for enzymatic assays. Mannitol dehydrogenase activity was assayed at 30°C using a standard method described previously [62]. The assay mixture contained 100 mM mannitol, 0.36 mM NAD in 25 mM Na 2-(*N-*cyclohexylamino) ethanesulfonic (CHES) buffer (pH 9.0), and cell extract (0.5 to 10g). Reactions were initiated with enzyme and NADH formation was monitored at 340 nm by using a SmartSpec Plus spectrophotometer (Bio-Rad). One unit of enzyme activity was defined as 1.0 mol of NADH produced in 1 min at 30°C.

### NAD^+^ and NADH content assays

A single colony of *nuo2*Δ/Δ strain was inoculated to YPD and incubated at 30°C. Logarithmically growing cells were washed three times with pre-warmed sterile water, resuspended in the same volume of YEP supplemented with 2% of mannitol, and continued to incubate at 37°C. Cell lysates were prepared with one-third volume of 0.5mm acid-washed glass beads (Sigma-Aldrich) using a high-throughput tissue homogenizer scientz-192 (Scientz, Ningbo, China), and NAD^+^ and NADH contents were measured using a NAD^+^/NADH Quantification Kit (Sigma-Aldrich).

### Measurement of reactive oxygen species (ROS) production

Intracellular ROS production was detected by a dihydrorhodamine 123 (DHR 123) staining method previously described [63]. Briefly, Cells of each strain were grown to mid-log stage in either YPD or YPM medium. After incubation, cells were harvested, washed, and resuspended in sterile PBS to a concentration of 2x10^7^ CFU/ml and stained with DHR123 (10mM) in the dark for 60min. After stained cells were collected by centrifugation and washed twice with PBS, total fluorescence of each sample was measured using a Cytoflow 2300 fluorescence spectrometer (Millipore Co., Billerica, MA) with excitation at 488 nm and emission at 525 nm.

### *In vivo* animal assays

We carried out two independent *in vivo* approaches to explore the effect of CI activity on *C*. *albicans* commensalism in murine GI tract. First, a competitive infection assay was performed using the mouse model of *C*. *albicans* commensalism, based on a previously reported protocol [51]. Briefly, groups of 6–8 week female BALB/c mice (Shanghai Laboratory Animal Center, Shanghai, China) were treated with penicillin (1500 μm/ml) and streptomycin (2 mg/ml) added to drinking water containing with or without 5% of glucose throughout the experiment beginning 3 d prior to inoculation. Mice were inoculated by oral gavage with 1x10^8^ CFUs of 1:1 mix of wild type and *nuo2*Δ/Δ. Fecal pellets were collected at various days post-inoculation and *C*. *albicans* was recovered by plating homogenates of mouse feces onto Sabouraud agar medium (with ampicillin 50 μm/ml, gentamicin 15 μm/ml). Relative abundances of strains in the infecting inoculum and after recovery from murine fecal pellets were determined by qPCR, using strain-specific primers (S2 Table). A second approach was carried out using a protocol described previously [64]. In this assay, most of procedures are exactly the same as the first one, unless each group of mice was inoculated by gavage with each of the three strains (wild type, *nuo2*Δ/Δ or NUO2AB; 10^8^ CFUs cells/mouse). Facal pellets were collected at various days post-inoculation, homogenized in PBS buffer, diluted and plated on Sabouraud agar medium (with ampicillin 50 μm/ml, gentamicin 15 μm/ml). Colonization was determined by counting the number of *C*. *albicans* cells grown on each plate. Data were analyzed using R and statistical significance (*P* values) was calculated using the post-hoc pairwise *t*-tests.

## Supporting information

S1 Methods Additional methods underlying the analysis described in the main text. Related references were also included.
(DOC)

S1 Fig Analysis of the role of CI subunits in biofilm formation in the culture condition when mannitol is the sole carbon source. As in Fig 1A, *C*. *albicans* cells, including wild type, the four CI mutants and their respective complemented derivatives, were grown as biofilms in Spider medium with shaking at 37°C. (A) Images of the wild type and CI mutant cells adhering to plastic following a 14-day incubation. (B) Quantitative measurement of biofilm dry weights for cells derived from indicated strains. “*”represents *P*<0.001 and “^#^” represents *P*<0.05 for WT vs. mutant. Values are the mean ± SD from two independent experiments with at least three replicates. AB indicates addback of one copy of the deleted gene to the corresponding mutant. (C) Analysis of the four CI mutants in a biofilm assay on plate with silicone square.
(TIF)

S2 Fig Cells lacking *NUO2* remain in the yeast form after an extended incubation period in mannitol condition. An overnight culture of *nuo2*Δ/Δ was diluted and re-inoculated in liquid (A) or on solid (B) YEP medium supplemented with 2% of mannitol. Cells were continued to incubate at 37°C and hyphal morphologies were visualized under microscopy after a long time incubation (14 days in liquid medium and 30 days on solid plate, respectively).
(TIF)

S3 Fig Partial restoration of hyphal growth in *nuo2*Δ/Δ mutant on glucose as the sole carbon source. As in Fig 2, overnight cultures of wild type, *nuo2*Δ/Δ and NUO2 AB strains were diluted and inoculated in either YEP or Spider medium using glucose (2%) as the sole carbon source. Cells were continued to incubate at 37°C and hyphal morphologies were visualized under microscopy. Experiments were repeated in triplicates. Shown are representative images of *C*. *albicans* cells at 1.5 h (A), 3 h (B) and 6 h (C) after incubation in medium containing glucose. Scale bars are 10μm.
(TIF)

S4 Fig Partial restoration of hyphal growth in *nuo2*Δ/Δ mutant on mannose as the sole carbon source. As in Fig 2, overnight cultures of wild type, *nuo2*Δ/Δ and NUO2 AB strains were diluted and inoculated in either YEP or Spider medium using mannose (2%) as the sole carbon source. Cells were continued to incubate at 37°C and hyphal morphologies were visualized under microscopy. Experiments were repeated in triplicates. Shown are representative images of *C*. *albicans* cells at 1.5 h (A), 3 h (B) and 6 h (C) after incubation in medium containing mannose. Scale bars are 10μm.
(TIF)

S5 Fig ATP production in indicated strains. Cells of wild type, *nuo2*Δ/Δ and NUO2 AB strains were incubated for 2h at 37°C in YEP medium supplemented with 2% of mannitol (A), glucose (B) or mannose (C). After an additional 2-hour incubation at 37°C, intracellular ATP levels were measured. “*” represents *P*<0.001 and “^#^” represents *P*<0.05 for WT vs. mutant. Values are the mean ± SD from two independent experiments with at least three replicates.
(TIF)

S6 Fig Cell proliferation of wild type, *nuo2*Δ/Δ and NUO2 AB strains under different growth conditions. Growth curves of indicated strains were performed in YEP medium supplemented with 2% glucose (A), 2% mannose (B), 2% mannitol (C), 0.1% glucose (D), 0.1% mannose (E), 2% mannitol+0.1% glucose (F), or 2% mannitol+0.1% mannose (G). Growth of each strain was monitored by OD_600_ measurements over a 36-h time course. The data shown are the average of two experiments done in duplicate. Error bars represent the standard deviations. Note that y axis scale of each figure may be different.
(TIF)

S7 Fig The effect of temperature change on cell morphology and proliferation in *nuo2*Δ/Δ mutant. (A) The *nuo2*Δ/Δ mutant cultures were grown overnight in a liquid YPD at 30°C, diluted in YEP medium supplemented with 2% mannitol, and incubate at either 30°C or 37°C with shaking. Sample were collected at the indicated intervals and cell morphology was visualized under microscopy. (B) The *nuo2*Δ/Δ mutant cells were treated in exactly the same way as described in A. Growth was monitored by OD_600_ measurements over a 36-h time course. The data shown are the average of two experiments done in duplicate. Error bars represent the standard deviations.
(TIF)

S8 Fig Inhibition of CI activity by rotenone treatment leads to a severe defect in hyphal growth but marginally affects the growth. (A) Effect of rotenone on cell growth in the basal stalts medium (BSM). Wild type cells were grown overnight in a liquid YPD at 30°C, pelleted, washed, and sub-cultured to OD_600_ ~0.05 in BSM supplemented with or without 80μg/ml rotenone (50mg/ml stock in 100% chloroform). Cells were continued to incubate at 30°C for 12h. Growth was monitored by optical density (O.D.) measurements. The data shown are the average of two experiments done in duplicate. As a control, the growth of *nuo2*Δ/Δ in BSM was also monitored during the time course. (B) Rotenone treatment inhibits hyphal growth of wild type *C*. *albicans* cells. Cells with an initial OD_600_ of 0.05 were grown at 37°C for 5h in BSM medium supplemented with or without rotenone (80μg/ml) and hyphal morphologies were visualized by microscopy. Experiments were repeated in triplicates. Shown are representative images of wild type cells after 5h incubation. Quantification of filamentation in 100 cells in each experiment is shown on the right. (C) Rotenone treatment downregulates the expression of *NUO2* in wild type. As in B, cells were harvested after 5h inbucation and relative transcript levels of *NUO2* were assessed by quantitative PCR (qPCR). Values obtained for each treatment were normalized against *ACT1* for each sample to give relative expression. “*” represents *P*<0.001 for untreated vs. treated sample. Error bars represent standard deviation of three independent biological replicates. (D) Cells lacking *NUO2* displayed decreased filamention in BSM. Shown are cell morphologies of wild type and *nuo2*Δ/Δ mutant grown at 37°C for 2h in BSM.
(TIF)

S9 Fig Cells lacking *NUO2* displayed increased level of intracellular ROS in a condition using mannitol as the sole carbon source. Measurement of ROS generation using DCFDA in WT, *nuo2*Δ/Δ and NUO2 AB cells after incubation on mannitol-containing medium. Exponential-phase cells (6h of growth; OD_600_ = 0.5) derived from indicated strains were harvested and ROS production was determined by DCF fluorescence. For each strain, three independent biological replicates were used. “*” represents *P*<0.001 for WT vs. mutant. The results represent the means of three independent experiments, and error bars represent the SDs.
(TIF)

S10 Fig ROS-triggered Hog1 activation is mannitol-dependent and requires a functional CI. Similar to Fig 5C, log-phase cells of WT and *nuo2*Δ/Δ strains, originally grown in YPD medium, were collected, washed and re-inoculated to equal volume of YEP medium supplemented with 2% of mannitol (A), glucose (B) or mannose (C). Cells were continued to grow at 37°C and collected at indicated time intervals for Western analysis. Protein extracts were fractionated by SDS-PAGE and immunoblotted with antibodies against either the phosphorylated or total Hog1. Immunoblotting with antibodies against the α-tubulin was used to control for variation in loading.
(TIF)

S11 Fig Expression of a Myc epitope-tagged Brg1 has no effect on the yeast-to-hypha transition of wild type *C*. *albicans*. The wild type *C*. *albicans* cells expressing a C-terminally myc-tagged version of Brg1 (Brg1-Myc) were grown under the yeast-inducing (30°C, YPD) or hyphae-inducing (37°C, YPM) conditions, respectively. Cell morphology was checked through a light microscopy.
(TIF)

S12 Fig Effect of *NUO2* deletion on transcription of genes encoding the core biofilm transcriptional regulators. Similar to Fig 5D, cells from WT, *nuo2*Δ/Δ and NUO2 AB strains were grown in YPM medium to mid-log stage. Relative transcript levels of listed genes (*BCR1*, *EFG1*, *NDT80*, *ROB1* and *TEC1*) were assessed by quantitative PCR (qPCR). Values obtained for each gene were normalized against *ACT1* for each sample to give relative expression. Error bars represent standard deviation of three independent biological replicates.
(TIF)

S13 Fig Replacement of the endogenous promoter with *TDH*3 promoter results in overexpression of *BRG1* RNA in *nuo2*Δ/Δ strain background. RT-qPCR analysis of *BRG1* RNA in *nuo2*Δ/Δ and *BRG1*^OE^ (*BRG1*^OE^+*nuo2*Δ/Δ) strains. Results are shown for strains propagated in mannitol-containing medium.
(TIF)

S14 Fig Overexpression of *BRG1* is sufficient to rescue biofilm defect of *nuo2*Δ/Δ mutant under the condition using mannitol as the sole carbon source. Cells from wild type, *nuo2*Δ/Δ, NUO2 AB or *BRG1*^OE^ (*BRG1*^OE^+*nuo2*Δ/Δ) were grown as biofilms in Spider medium with shaking at 37°C and samples were analyzed by (A) cell adhesion on plate with silicone square and (B) biofilm dry weights. Values are the mean ± SD from two independent experiments with at least three replicates.
(TIF)

S15 Fig Overexpression of *BRG1* is incapable of rescuing the growth defect of *nuo2*Δ/Δ mutant under the condition using mannitol is the sole carbon source. Cells from the *nuo2*Δ/Δ or *BRG1*^OE^ (*BRG1*^OE^ + *nuo2*Δ/Δ) strain were sub-cultured to OD_600_ ~0.05 and continued to grow at 30°C for 36h. Growth curves of indicated strains were performed in YEP medium supplemented with 2% glucose (A) or 2% mannitol (B). Growth of each strain was monitored by OD_600_ measurements over a 36-h time course. The data shown are the average of two experiments done in duplicate. Note that y axis scales of A and B are different.
(TIF)

S16 Fig Effect of NAC supplementation on Hog1 activation and Brg1 expression in the wild type *C*. *albicans*. Similar to Fig 5G, the wild type cells grown in mannitol-containing YEP were treated with different doses of the antioxidant N-Acetyl Cysteine (NAC) or were not treated. Samples were taken after 5 hours incubation at 37°C and protein extracts were assayed by sequential immunoblotting with antibodies against Myc epitope, phosphorylated Hog1, total Hog1 and α-tubulin (loading control).
(TIF)

S17 Fig NAC treatment partially restores biofilm formation in *nuo2*Δ/Δ mutant under the mannitol condition. Cells from wild type or *nuo2*Δ/Δ were grown as biofilms in mannitol-containing Spider medium in the presence or absence of 20mM NAC. Samples were analyzed by (A) cell adhesion on plate with silicone square and (B) biofilm dry weights. Values are the mean ± SD from two independent experiments with at least three replicates.
(TIF)

S18 Fig The peak level of Brg1 in *nuo2*Δ/Δ cells treated with NAC is less than that of untreated wild type under the mannitol condition. As in S16 Fig, log-phase of wild type or *nuo2*Δ/Δ cells grown in mannitol-containing YEP medium were treated with different doses of NAC or were not treated. Samples were taken after 5 hours incubation at 37°C and protein extracts were assayed by immunoblotting with antibodies against Myc epitope. Immunoblotting with antibodies against the α-tubulin was used to control for variation in loading.
(TIF)

S19 Fig NAC treatment is incapable of rescuing the growth defect of *nuo2*Δ/Δ mutant under the condition using mannitol as the sole carbon source. Cells from *nuo2*Δ/Δ (A), wild type (B) or NUO2 AB (C) were sub-cultured to OD_600_ ~0.05 and continued to grow at 30°C for 36h. Growth curves were performed in mannitol-containing YEP medium in the presence or absence of 20mM NAC. Growth of each strain was monitored by OD_600_ measurements over a 36-h time course. The data shown are the average of two experiments done in duplicate. Note that the y axis scale of each figure may be different.
(TIF)

S20 Fig NAD^+^ alone is insufficient to induce Brg1 expression. Log-phase of *nuo2*Δ/Δ mutant cells were treated with NAC (10mM), NAD^+^ (10mM or 20mM), or both and protein extracts were analyzed by immunoblotting with antibodies against Myc epitope and α-tubulin (loading control).
(TIF)

S21 Fig Comparison of transcript levels of a list of classic hyphae-specific genes (HSGs) in *C*. *albicans cells grown* under different conditions. (A) Cells from wild type, *nuo2*Δ/Δ and NUO2 AB strains were grown on YPD or YPM medium to mid-log stage. Relative transcript levels of four HSGs including *HWP1*, *ALS3*, *ECE1* and *HYR1* were assessed by quantitative PCR (qPCR). Values obtained for each gene were normalized against *ACT1* for each sample to give relative expression. “*”represents *P*<0.001 for WT vs. mutant. Error bars represent standard deviation of three independent biological replicates. (B) An overnight cultures of *nuo2*Δ/Δ cells was diluted and inoculated in mannitol-containing YEP medium in the presence or absence of NAC (20mM), and continuted to incubate at 37°C for 6-8h. Transcript levels of the same set of genes mentioned above were analyzed by RT-qPCR.
(TIF)

S22 Fig Differential impact of glycerol and GlcNAc on hyphal morphogenesis of wild type and *nuo2*Δ/Δ mutant. Log-phase cells of WT (A) and *nuo2*Δ/Δ (B) strains, originally grown in YPD medium, were collected, washed and re-inoculated to equal volume of YEP medium supplemented with 2% of glycerol or GlcNAc. Cells were continued to grow at 37°C for 4h and cell morphologies were visualized under microscopy.
(TIF)

S23 Fig Effect of glycerol or GlcNAc on Brg1 expression and Hog1 activation in the wild type *C*. *albicans*. (A) Treating the wild type cells with glycerol induced robust Brg1 expression while the level of phosphorylated Hog1 was almost undetected. As in Fig 6C, log-phase of wild type cells, orginally grown in YPD, were collected, washed and re-inoculated to equal volume of YEP medium supplemented with 2% of glycerol. Cells were continued to grow at 37°C and collected at indicated time points for Western analysis, using antibodies against Myc epitope, phosphorylated Hog1, total Hog1 and α-tubulin (loading control) (B) GlcNAc treatment increased Brg1 expression and Hog1 phosphorylation in wild type *C*. *albicans*. Cells were treated exactly the same as in (A), with the exception that 1% of GlcNAc were used.
(TIF)

S24 Fig Wild type *C*. *albicans* strongly outcompetes *nuo2*Δ/Δ mutant in mixed competitive infections. 6–8 week female BLAB/c mice were infected by oral gavage with 10^8^ CFUs of a 1:1 mixtures wild type (SN250) and *nuo2*Δ/Δ mutant. Fecal pellets were collected at specified intervals and homogenates of mouse feces were plated onto Sabouraud agar medium (with ampicillin 50 μm/ml, gentamicin 15 μm/ml). Shown is a representative image of mixed *C*. *albicans* cells isolated from mouse fecal samples at day 5 after infection. Small colonies (typified with arrows) were confirmed by PCR to be derived from *nuo2*Δ/Δ mutant.
(TIF)

S25 Fig Diet change did not affect gastrointestinal colonization of wild type *C*. *albicans*. As in Fig 6F, a similar commensalism experiment were conducted. All manipulations were the same with the exception that the wild type *C*. *albicans* strain was used.
(TIF)

S1 Table Strains used in this study. (XLSX)

S2 Table Primers used in this study. (XLSX)

S3 Table Plasmids used in this study. (XLSX)

S4 Table A list of biofilm regulators identified from our screen. (XLSX)
