# Supplementary material for: Mitochondrial complex I bridges a connection between regulation of carbon flexibility and gastrointestinal commensalism in the human fungal pathogen Candida albicans
Source: PLoS Pathog. 2017 Jun 1;13(6):e1006414. doi: 10.1371/journal.ppat.1006414 (PMC5469625; doi:10.1371/journal.ppat.1006414)
Supplement: S1 Methods — Related references were also included. (DOC) [file ppat.1006414.s001.doc]

**S1 Methods. Supporting methods including additional methods and references**

**Media**

Basal salts medium (BSM; 0.5% NH_4_SO_4_, 0.02% MgSO_4_, 0.5% NaCl, 0.25% K_2_HPO_4_, 0.001% biotin, pH6.5, supplemented with complete amino acids) was prepared following a published protocol [1]. The CI inhibitor rotenone (Sigma-Aldrich, USA) was prepared and stored as a stock of 50mg/ml in 100% chloroform.

**Measurement of reactive oxygen species (ROS) production using a DCFDA staining method**

Intracellular ROS production was detected by a method previously described . This method depends on intracellular deacetylation and the oxidation of 2′,7′-dichlorodihydrofluorescein diacetate (DCFDA) to the corresponding fluorescent compound. Cells (10^7^ cells/ml) of each strain were resuspended in 1ml of PBS and stained with 20 μM DCFDA at 30°C for 30 min in the dark. After stained cells were collected by centrifugation and washed twice with PBS, fluorescence intensity of each sample was measured using a Cytoflow 2300 fluorescence spectrometer (Millipore Co., Billerica, MA) with excitation at 480 nm and emission at 530 nm. The arbitrary units were based directly on fluorescence intensity.

**ATP measurement**

Cells of wild type, *nuo2*Δ/Δ and NUO2 AB strains were pre-grown at 30°C to reach an OD_600_ of 0.8-1.0, harvested and washed with sterile water, and then re-inoculated to 50ml of YEP supplemented with different carbon sources for another 2-hour incubation at 37°C. The adenosine-5’-triphophate (ATP) content of *C. albicans* strains was determined with the ATP-Lite Assay Kit (Vigorous biotechnology, Beijing, China) following the procedures described by the manufacturer.

**References**

1. Torosantucci A, Angiolella L, Filesi C, Cassone A. Protein synthesis and amino acid pool during yeast-mycelial transition induced by N-acetyl-D-glucosamine in Candida albicans. Journal of general microbiology. 1984;130(12):3285-93. doi: 10.1099/00221287-130-12-3285. PubMed PMID: 6394717.

2.Nett JE, Cain MT, Crawford K, Andes DR. Optimizing a Candida biofilm microtiter plate model for measurement of antifungal susceptibility by tetrazolium salt assay. Journal of clinical microbiology. 2011;49(4):1426-33. doi: 10.1128/JCM.02273-10. PubMed PMID: 21227984; PubMed Central PMCID: PMC3122839.
