# Supplementary figures and images for: Mitochondrial complex I bridges a connection between regulation of carbon flexibility and gastrointestinal commensalism in the human fungal pathogen Candida albicans
Source: PLoS Pathog. 2017 Jun 1;13(6):e1006414. doi: 10.1371/journal.ppat.1006414 (PMC5469625; doi:10.1371/journal.ppat.1006414)

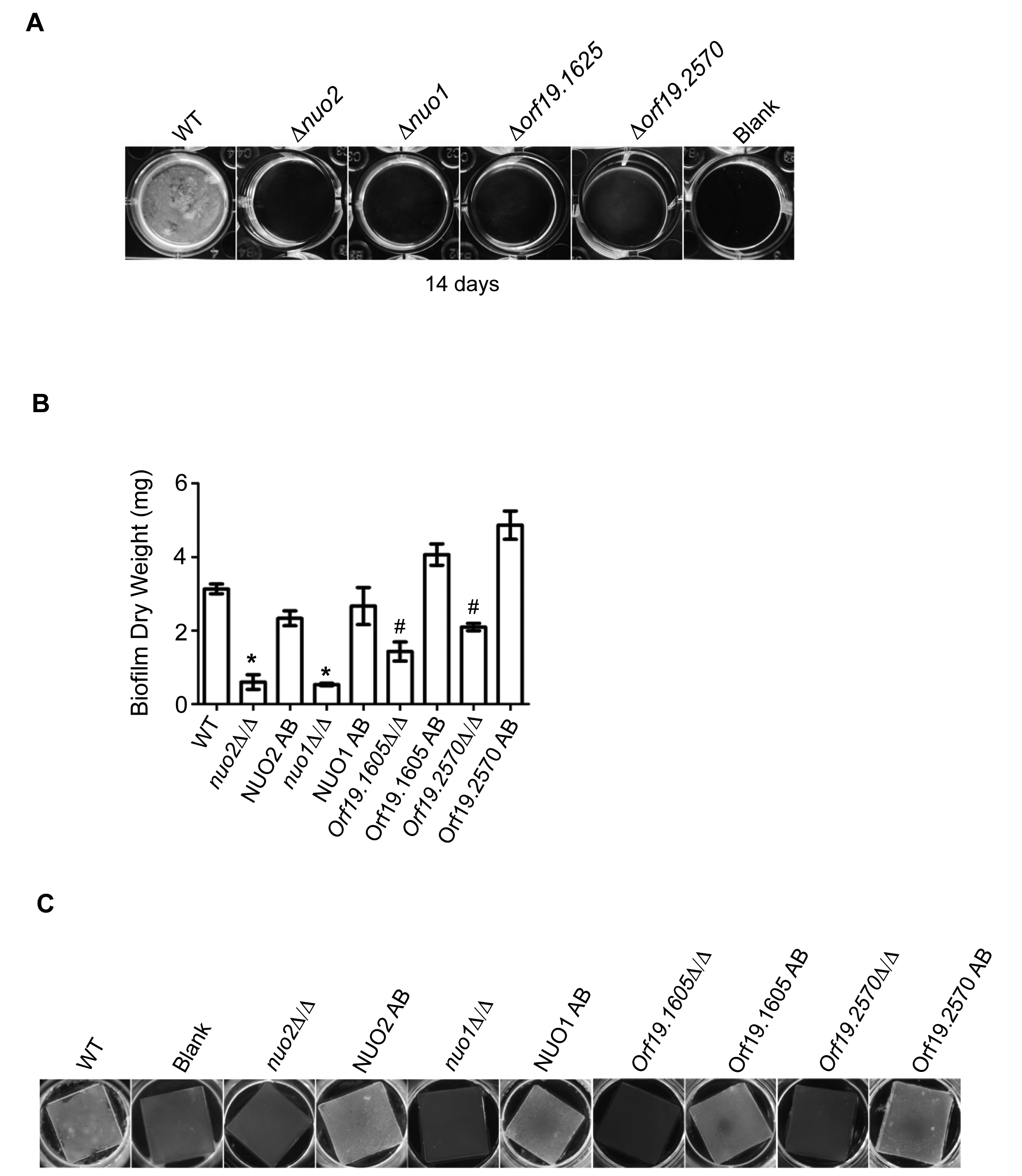

Supplement: S1 Fig — As in Fig 1A, C. albicans cells, including wild type, the four CI mutants and their respective complemented derivatives, were grown as biofilms in Spider medium with shaking at 37°C. (A) Images of the wild type and CI mutant cells adhering to plastic following a 14-day incubation. (B) Quantitative measurement of biofilm dry weights for cells derived from indicated strains. “*”represents P<0.001 and “#” represents P<0.05 for WT vs. mutant. Values are the mean ± SD from two independent experiments with at least three replicates. AB indicates addback of one copy of the deleted gene to the corresponding mutant. (C) Analysis of the four CI mutants in a biofilm assay on plate with silicone square. (TIF) [file ppat.1006414.s002.tif]

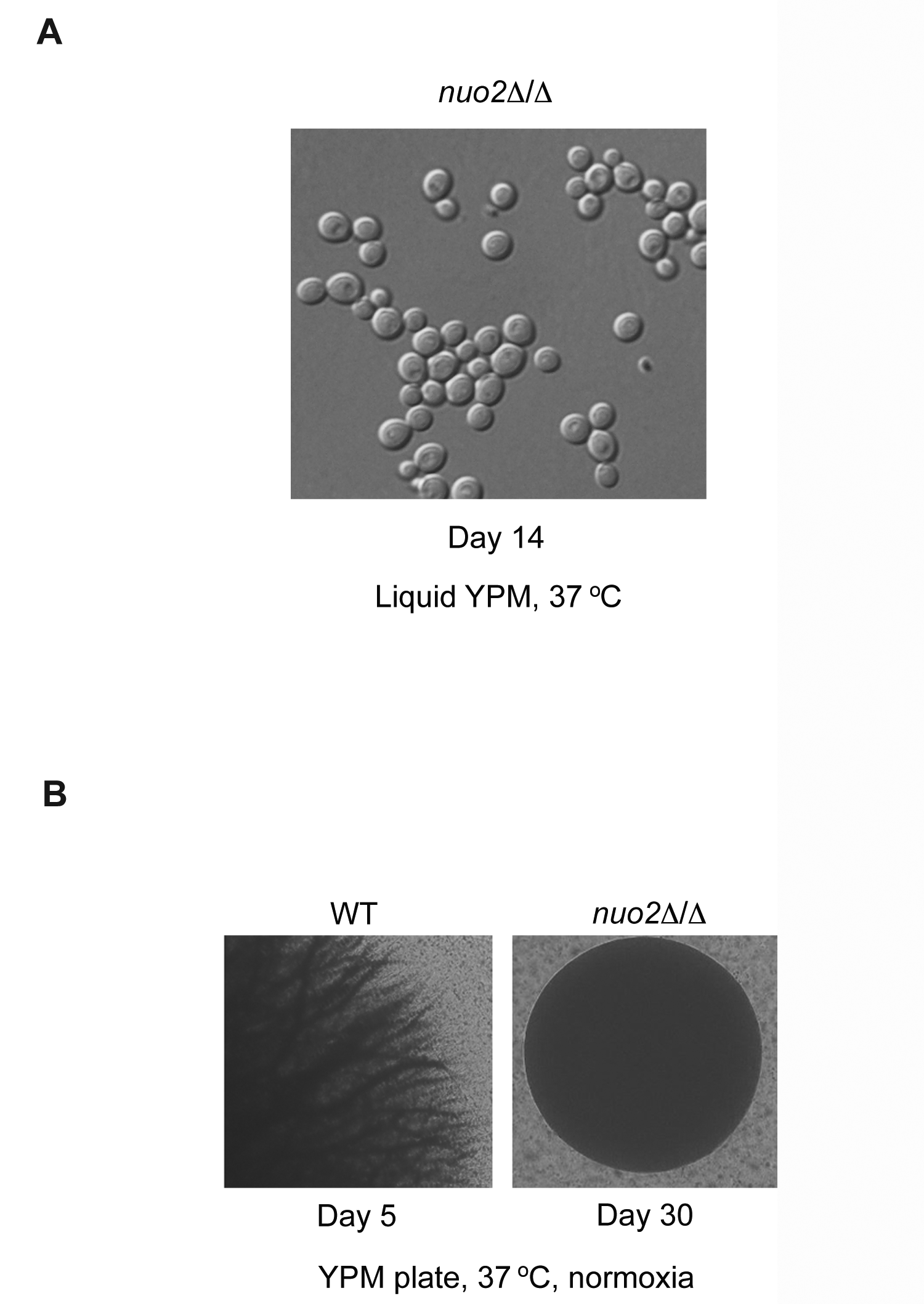

Supplement: S2 Fig — An overnight culture of nuo2Δ/Δ was diluted and re-inoculated in liquid (A) or on solid (B) YEP medium supplemented with 2% of mannitol. Cells were continued to incubate at 37°C and hyphal morphologies were visualized under microscopy after a long time incubation (14 days in liquid medium and 30 days on solid plate, respectively). (TIF) [file ppat.1006414.s003.tif]

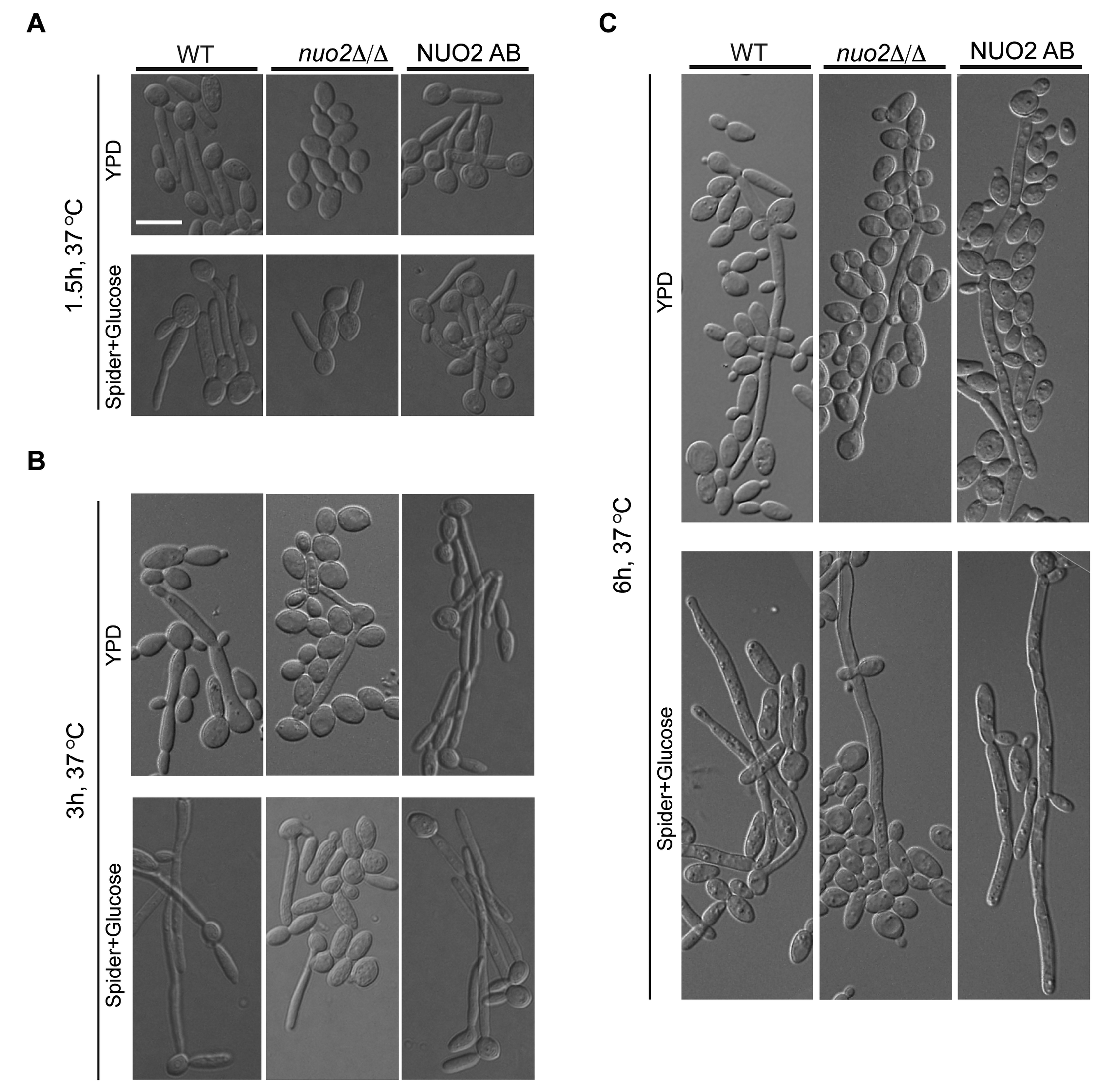

Supplement: S3 Fig — As in Fig 2, overnight cultures of wild type, nuo2Δ/Δ and NUO2 AB strains were diluted and inoculated in either YEP or Spider medium using glucose (2%) as the sole carbon source. Cells were continued to incubate at 37°C and hyphal morphologies were visualized under microscopy. Experiments were repeated in triplicates. Shown are representative images of C. albicans cells at 1.5 h (A), 3 h (B) and 6 h (C) after incubation in medium containing glucose. Scale bars are 10μm. (TIF) [file ppat.1006414.s004.tif]

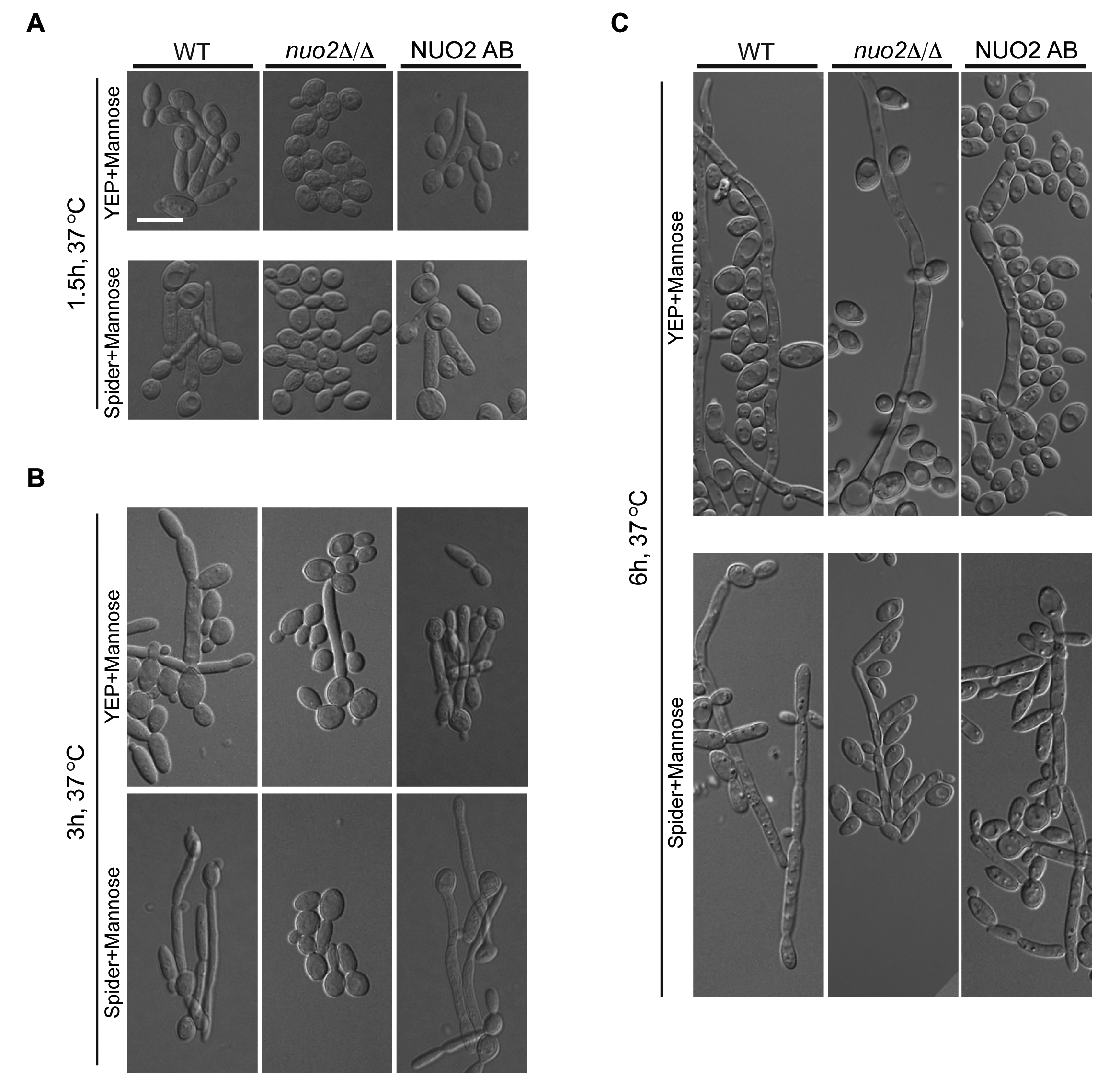

Supplement: S4 Fig — As in Fig 2, overnight cultures of wild type, nuo2Δ/Δ and NUO2 AB strains were diluted and inoculated in either YEP or Spider medium using mannose (2%) as the sole carbon source. Cells were continued to incubate at 37°C and hyphal morphologies were visualized under microscopy. Experiments were repeated in triplicates. Shown are representative images of C. albicans cells at 1.5 h (A), 3 h (B) and 6 h (C) after incubation in medium containing mannose. Scale bars are 10μm. (TIF) [file ppat.1006414.s005.tif]

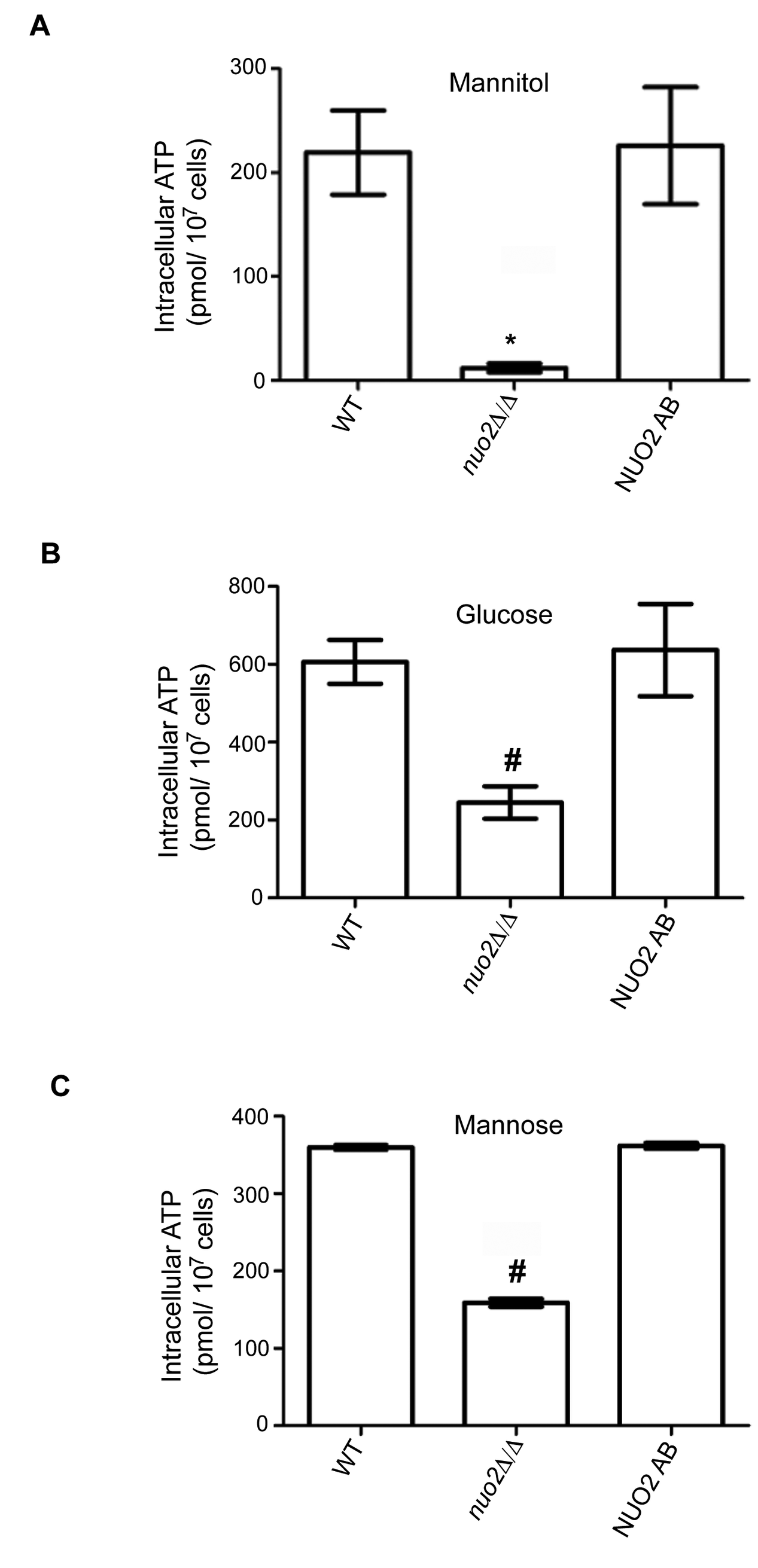

Supplement: S5 Fig — Cells of wild type, nuo2Δ/Δ and NUO2 AB strains were incubated for 2h at 37°C in YEP medium supplemented with 2% of mannitol (A), glucose (B) or mannose (C). After an additional 2-hour incubation at 37°C, intracellular ATP levels were measured. “*” represents P<0.001 and “#” represents P<0.05 for WT vs. mutant. Values are the mean ± SD from two independent experiments with at least three replicates. (TIF) [file ppat.1006414.s006.tif]

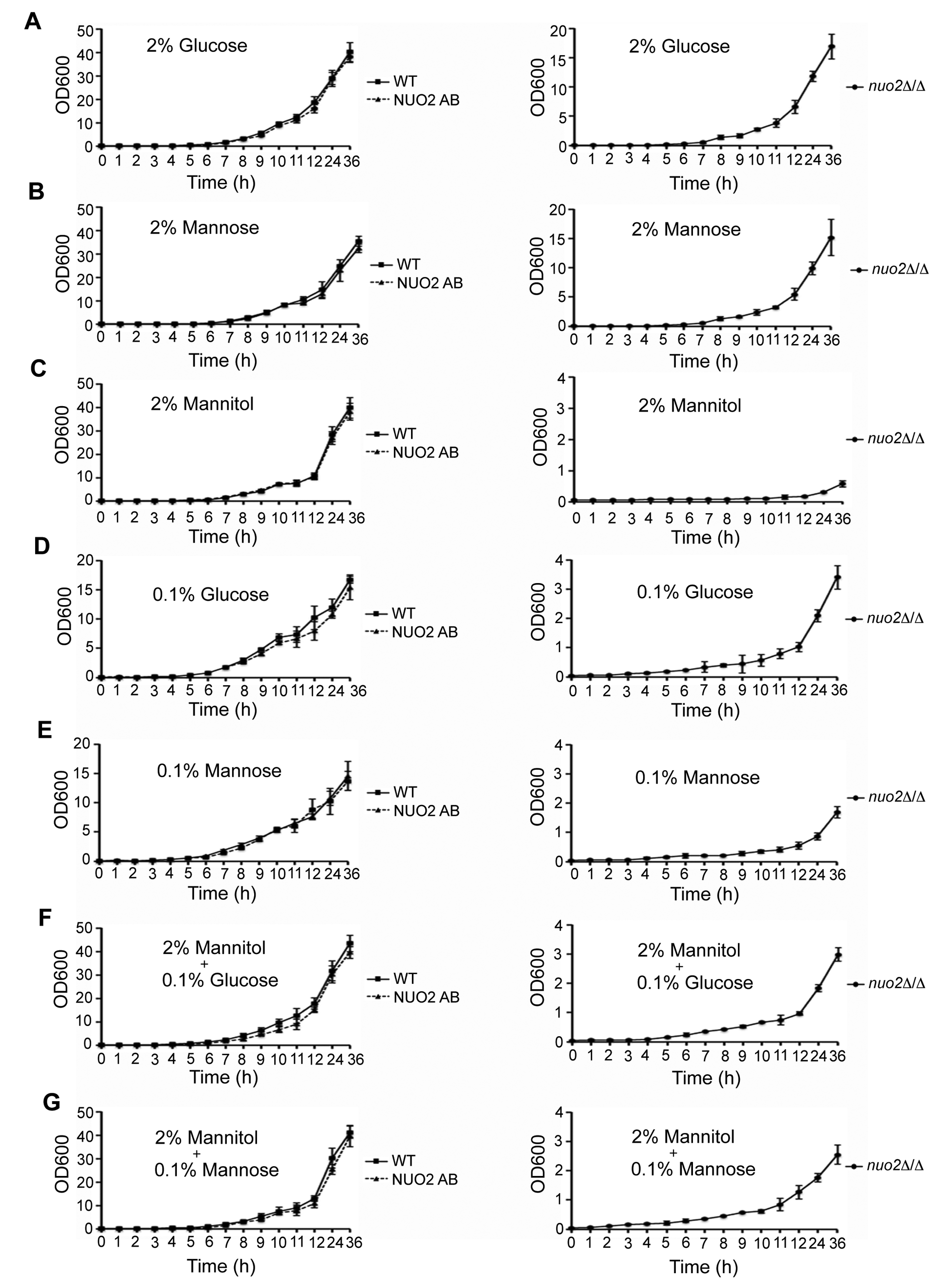

Supplement: S6 Fig — Growth curves of indicated strains were performed in YEP medium supplemented with 2% glucose (A), 2% mannose (B), 2% mannitol (C), 0.1% glucose (D), 0.1% mannose (E), 2% mannitol+0.1% glucose (F), or 2% mannitol+0.1% mannose (G). Growth of each strain was monitored by OD600 measurements over a 36-h time course. The data shown are the average of two experiments done in duplicate. Error bars represent the standard deviations. Note that y axis scale of each figure may be different. (TIF) [file ppat.1006414.s007.tif]

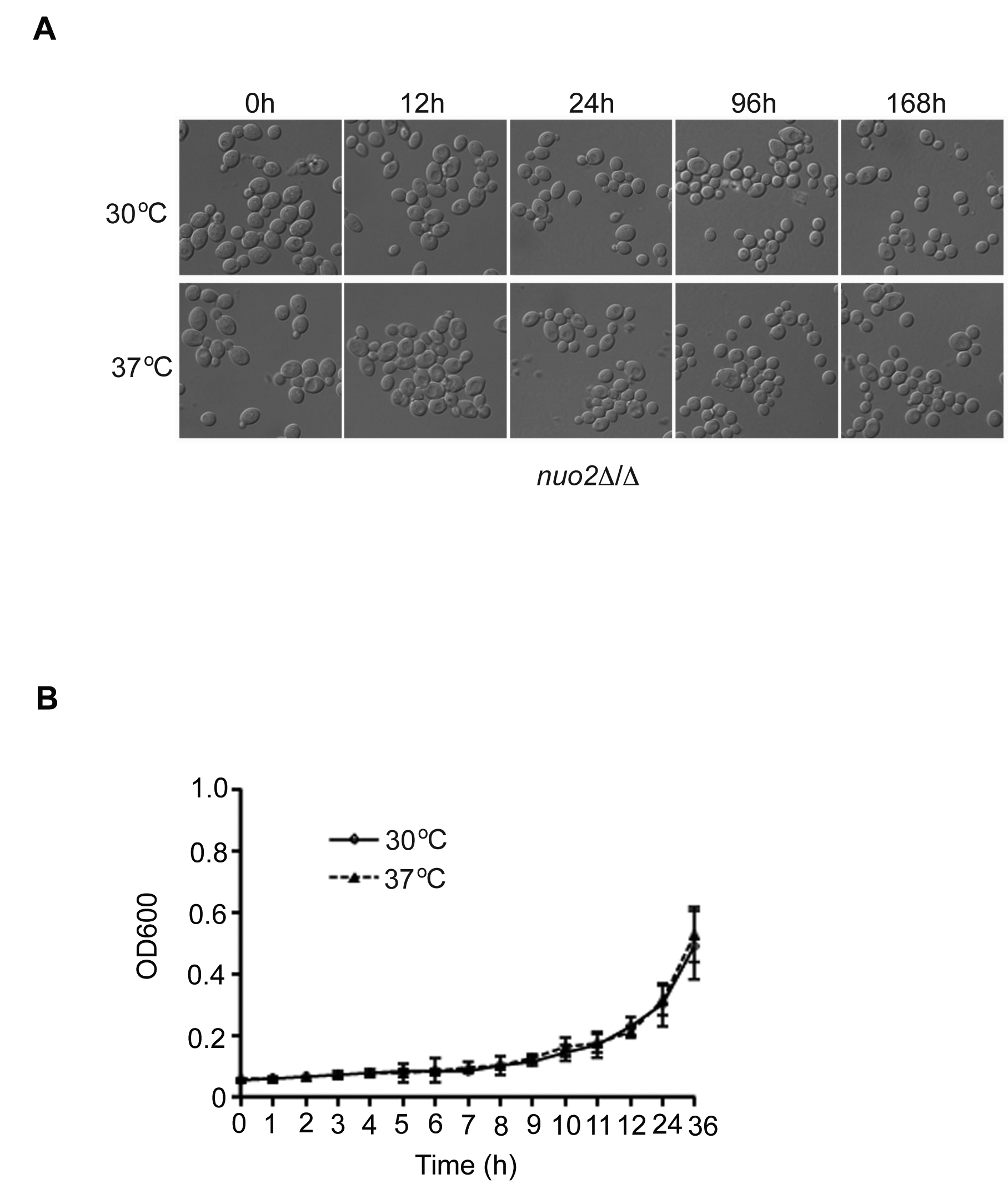

Supplement: S7 Fig — (A) The nuo2Δ/Δ mutant cultures were grown overnight in a liquid YPD at 30°C, diluted in YEP medium supplemented with 2% mannitol, and incubate at either 30°C or 37°C with shaking. Sample were collected at the indicated intervals and cell morphology was visualized under microscopy. (B) The nuo2Δ/Δ mutant cells were treated in exactly the same way as described in A. Growth was monitored by OD600 measurements over a 36-h time course. The data shown are the average of two experiments done in duplicate. Error bars represent the standard deviations. (TIF) [file ppat.1006414.s008.tif]

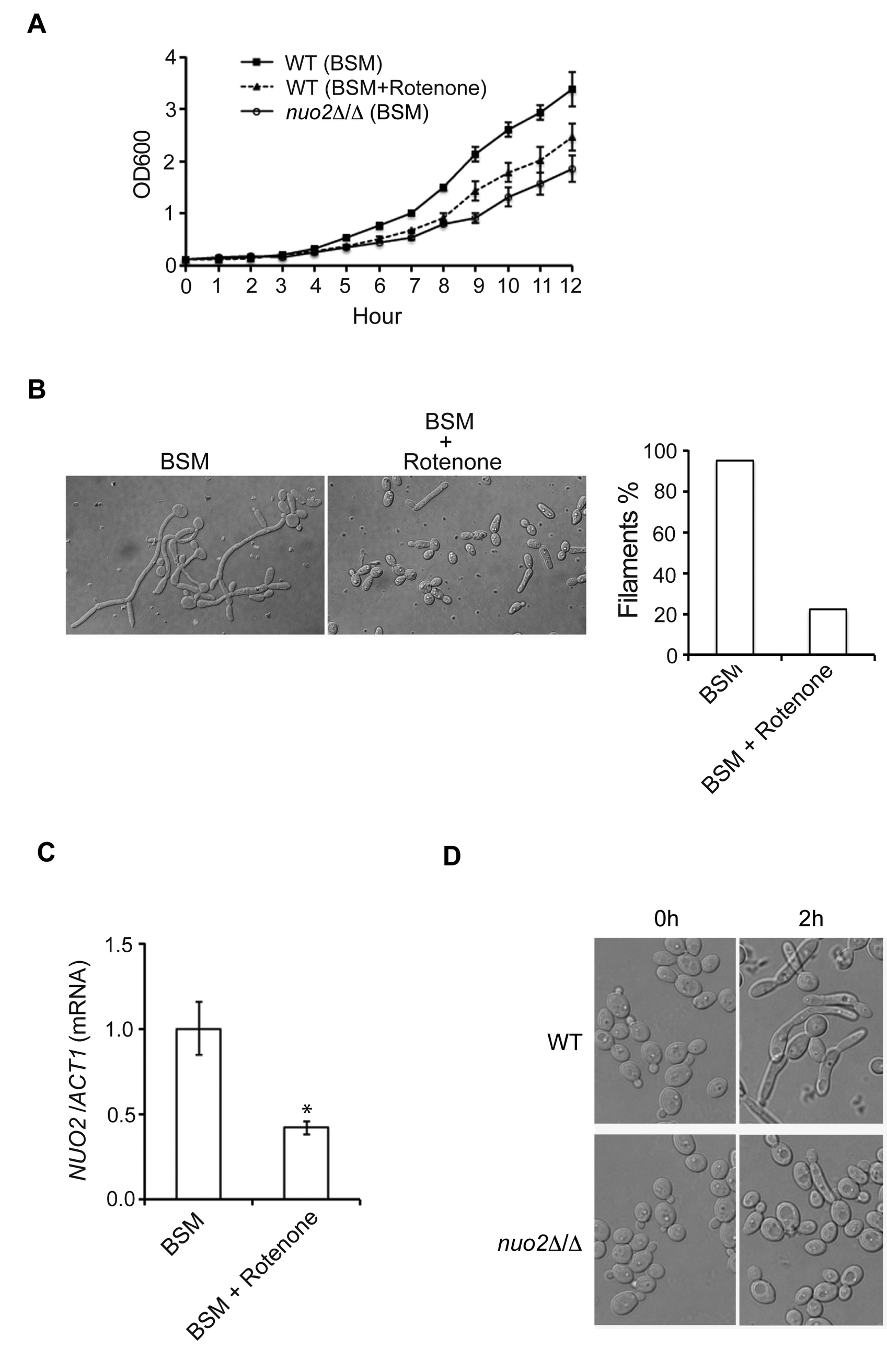

Supplement: S8 Fig — (A) Effect of rotenone on cell growth in the basal stalts medium (BSM). Wild type cells were grown overnight in a liquid YPD at 30°C, pelleted, washed, and sub-cultured to OD600 ~0.05 in BSM supplemented with or without 80μg/ml rotenone (50mg/ml stock in 100% chloroform). Cells were continued to incubate at 30°C for 12h. Growth was monitored by optical density (O.D.) measurements. The data shown are the average of two experiments done in duplicate. As a control, the growth of nuo2Δ/Δ in BSM was also monitored during the time course. (B) Rotenone treatment inhibits hyphal growth of wild type C. albicans cells. Cells with an initial OD600 of 0.05 were grown at 37°C for 5h in BSM medium supplemented with or without rotenone (80μg/ml) and hyphal morphologies were visualized by microscopy. Experiments were repeated in triplicates. Shown are representative images of wild type cells after 5h incubation. Quantification of filamentation in 100 cells in each experiment is shown on the right. (C) Rotenone treatment downregulates the expression of NUO2 in wild type. As in B, cells were harvested after 5h inbucation and relative transcript levels of NUO2 were assessed by quantitative PCR (qPCR). Values obtained for each treatment were normalized against ACT1 for each sample to give relative expression. “*” represents P<0.001 for untreated vs. treated sample. Error bars represent standard deviation of three independent biological replicates. (D) Cells lacking NUO2 displayed decreased filamention in BSM. Shown are cell morphologies of wild type and nuo2Δ/Δ mutant grown at 37°C for 2h in BSM. (TIF) [file ppat.1006414.s009.tif]

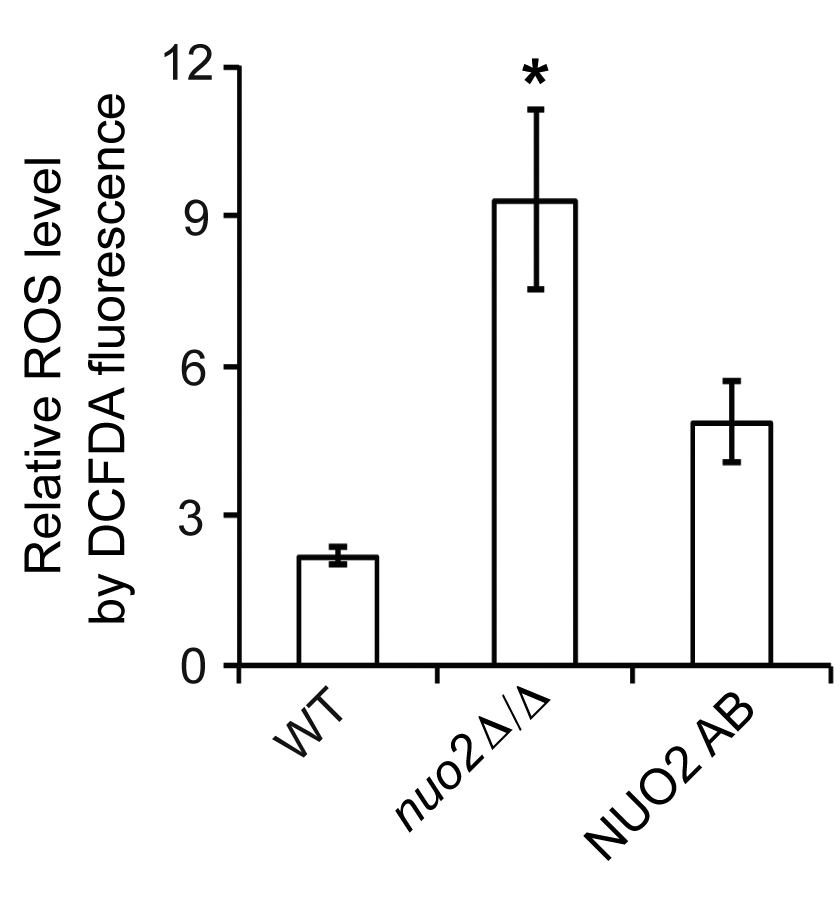

Supplement: S9 Fig — Measurement of ROS generation using DCFDA in WT, nuo2Δ/Δ and NUO2 AB cells after incubation on mannitol-containing medium. Exponential-phase cells (6h of growth; OD600 = 0.5) derived from indicated strains were harvested and ROS production was determined by DCF fluorescence. For each strain, three independent biological replicates were used. “*” represents P<0.001 for WT vs. mutant. The results represent the means of three independent experiments, and error bars represent the SDs. (TIF) [file ppat.1006414.s010.tif]

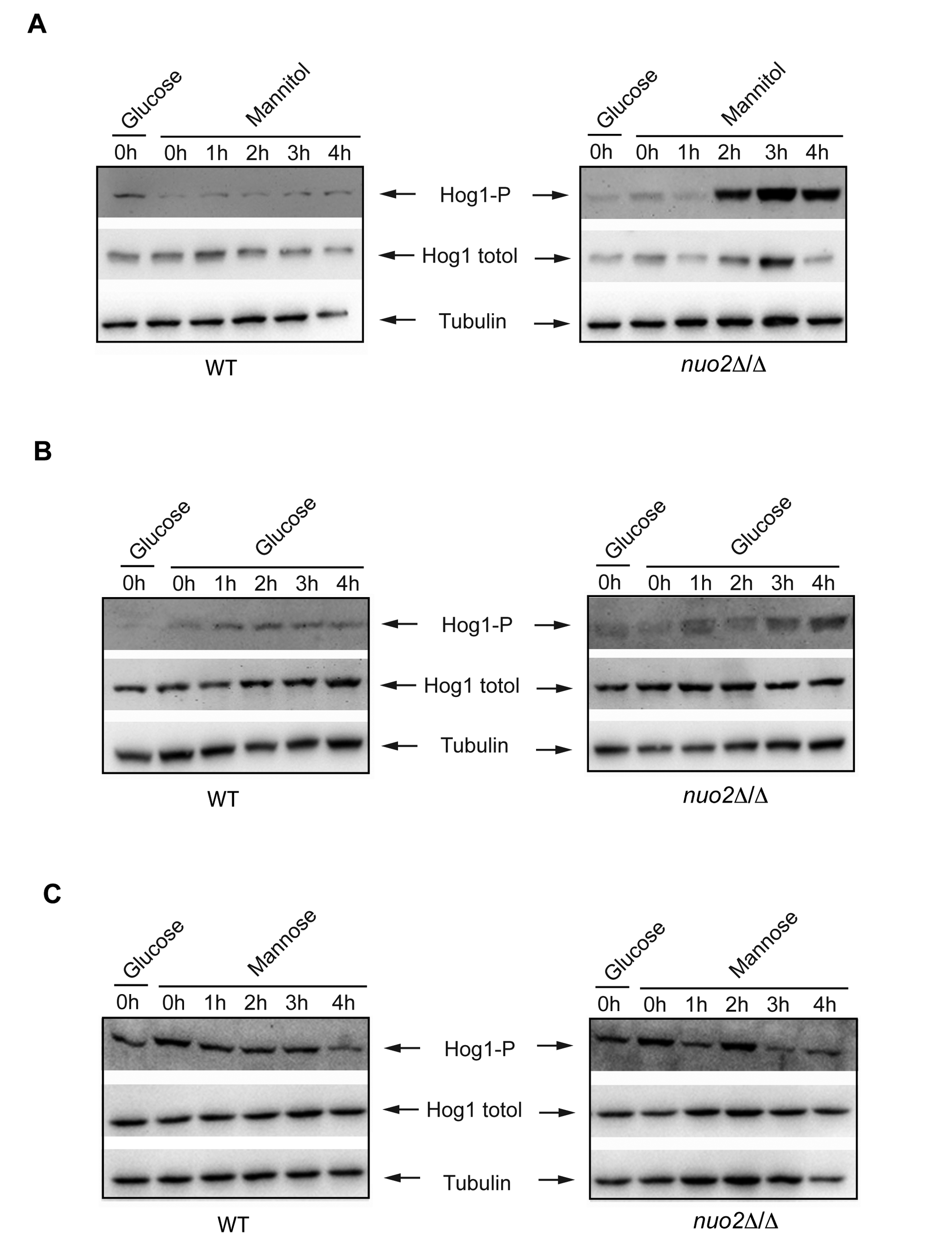

Supplement: S10 Fig — Similar to Fig 5C, log-phase cells of WT and nuo2Δ/Δ strains, originally grown in YPD medium, were collected, washed and re-inoculated to equal volume of YEP medium supplemented with 2% of mannitol (A), glucose (B) or mannose (C). Cells were continued to grow at 37°C and collected at indicated time intervals for Western analysis. Protein extracts were fractionated by SDS-PAGE and immunoblotted with antibodies against either the phosphorylated or total Hog1. Immunoblotting with antibodies against the α-tubulin was used to control for variation in loading. (TIF) [file ppat.1006414.s011.tif]

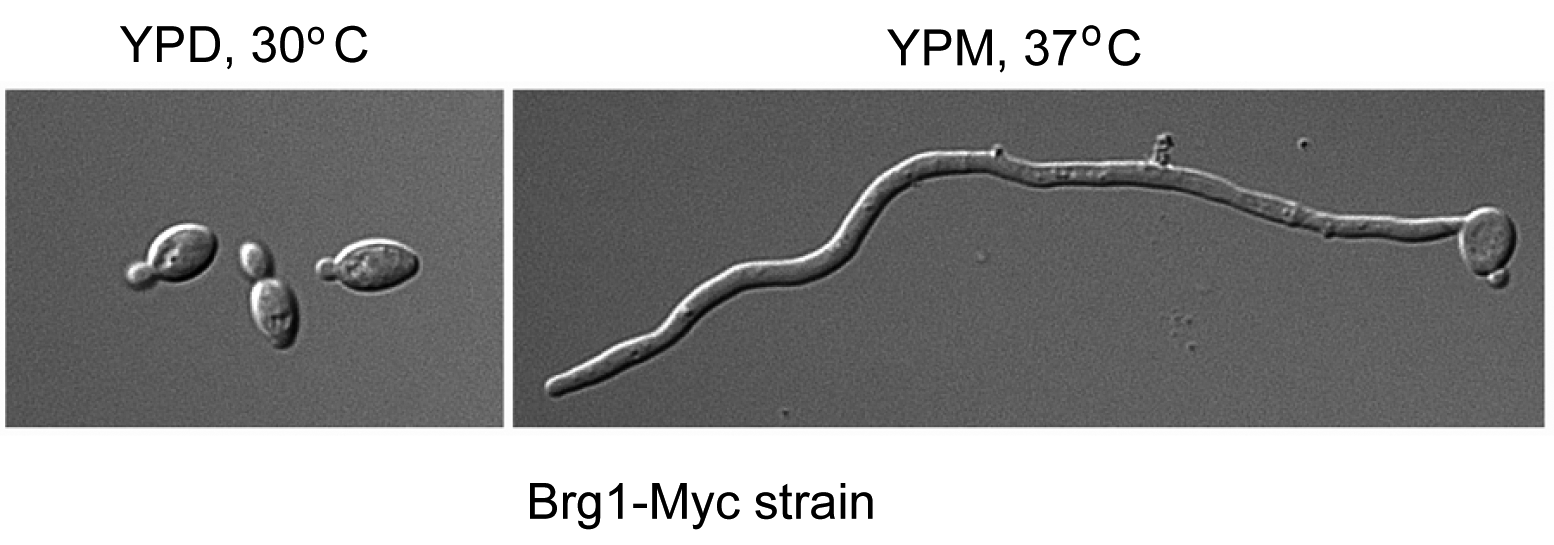

Supplement: S11 Fig — The wild type C. albicans cells expressing a C-terminally myc-tagged version of Brg1 (Brg1-Myc) were grown under the yeast-inducing (30°C, YPD) or hyphae-inducing (37°C, YPM) conditions, respectively. Cell morphology was checked through a light microscopy. (TIF) [file ppat.1006414.s012.tif]

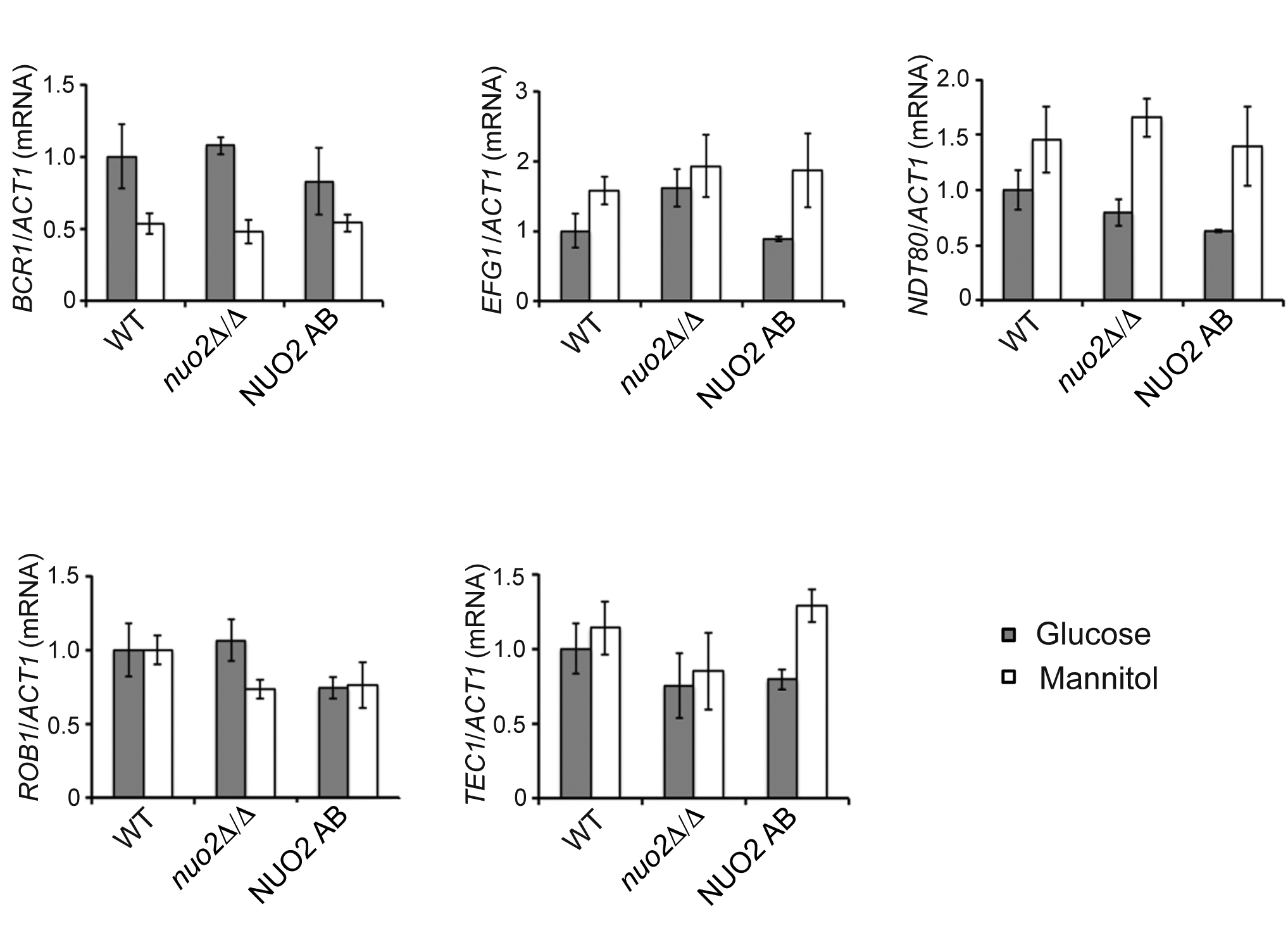

Supplement: S12 Fig — Similar to Fig 5D, cells from WT, nuo2Δ/Δ and NUO2 AB strains were grown in YPM medium to mid-log stage. Relative transcript levels of listed genes (BCR1, EFG1, NDT80, ROB1 and TEC1) were assessed by quantitative PCR (qPCR). Values obtained for each gene were normalized against ACT1 for each sample to give relative expression. Error bars represent standard deviation of three independent biological replicates. (TIF) [file ppat.1006414.s013.tif]

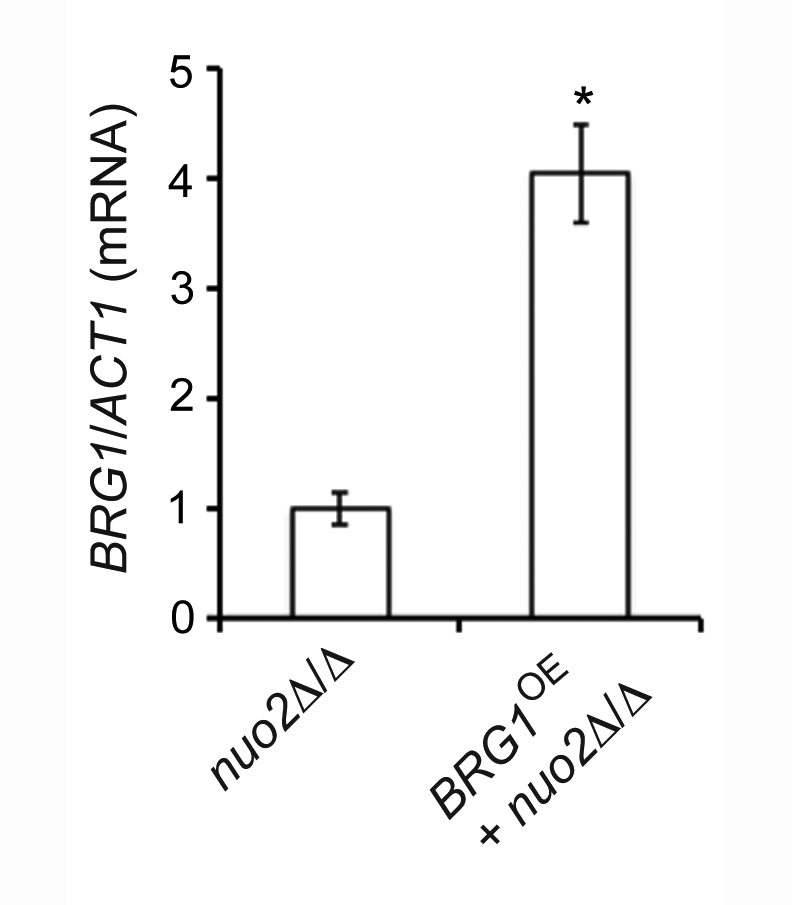

Supplement: S13 Fig — RT-qPCR analysis of BRG1 RNA in nuo2Δ/Δ and BRG1OE (BRG1OE+nuo2Δ/Δ) strains. Results are shown for strains propagated in mannitol-containing medium. (TIF) [file ppat.1006414.s014.tif]

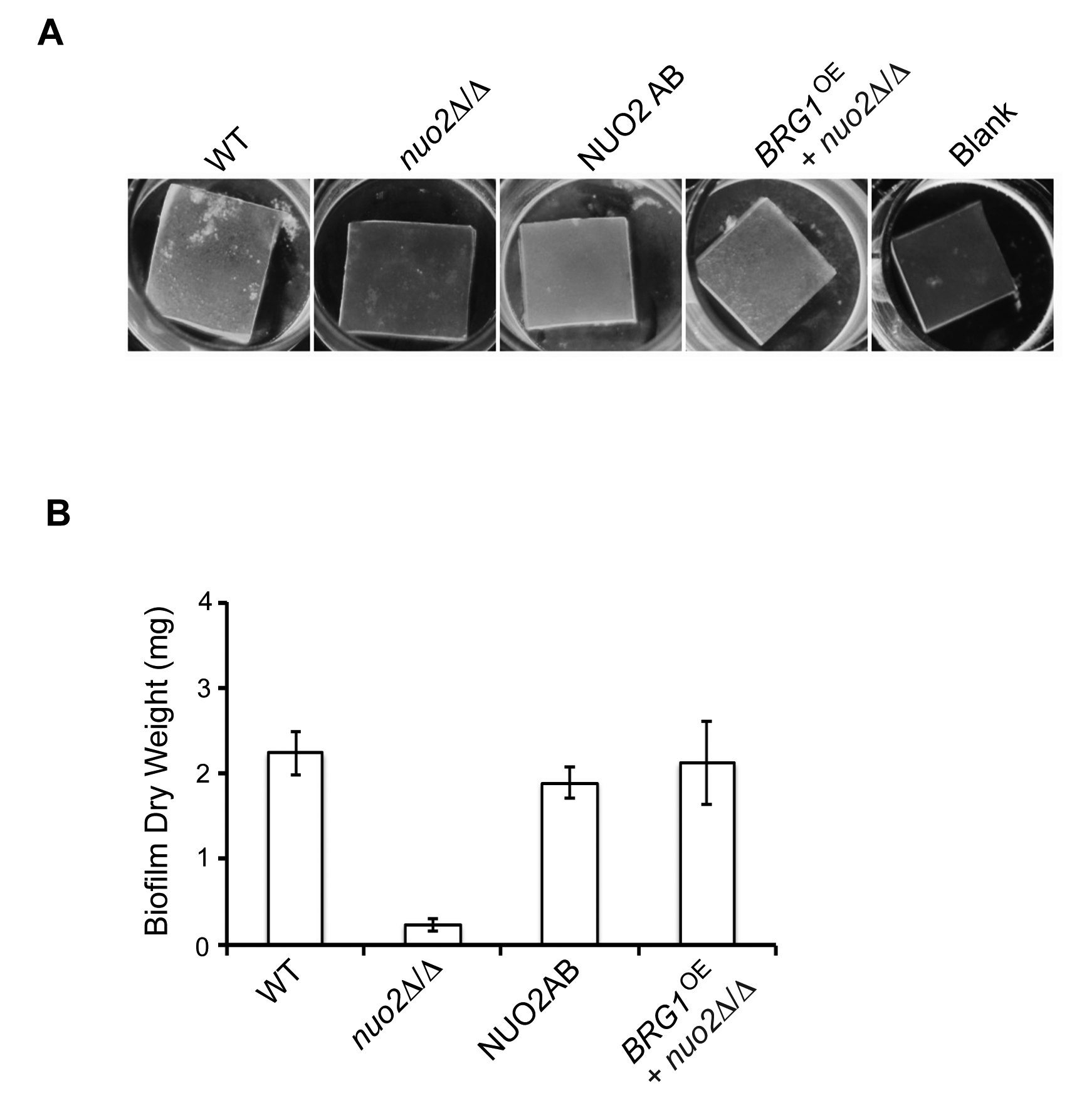

Supplement: S14 Fig — Cells from wild type, nuo2Δ/Δ, NUO2 AB or BRG1OE (BRG1OE+nuo2Δ/Δ) were grown as biofilms in Spider medium with shaking at 37°C and samples were analyzed by (A) cell adhesion on plate with silicone square and (B) biofilm dry weights. Values are the mean ± SD from two independent experiments with at least three replicates. (TIF) [file ppat.1006414.s015.tif]

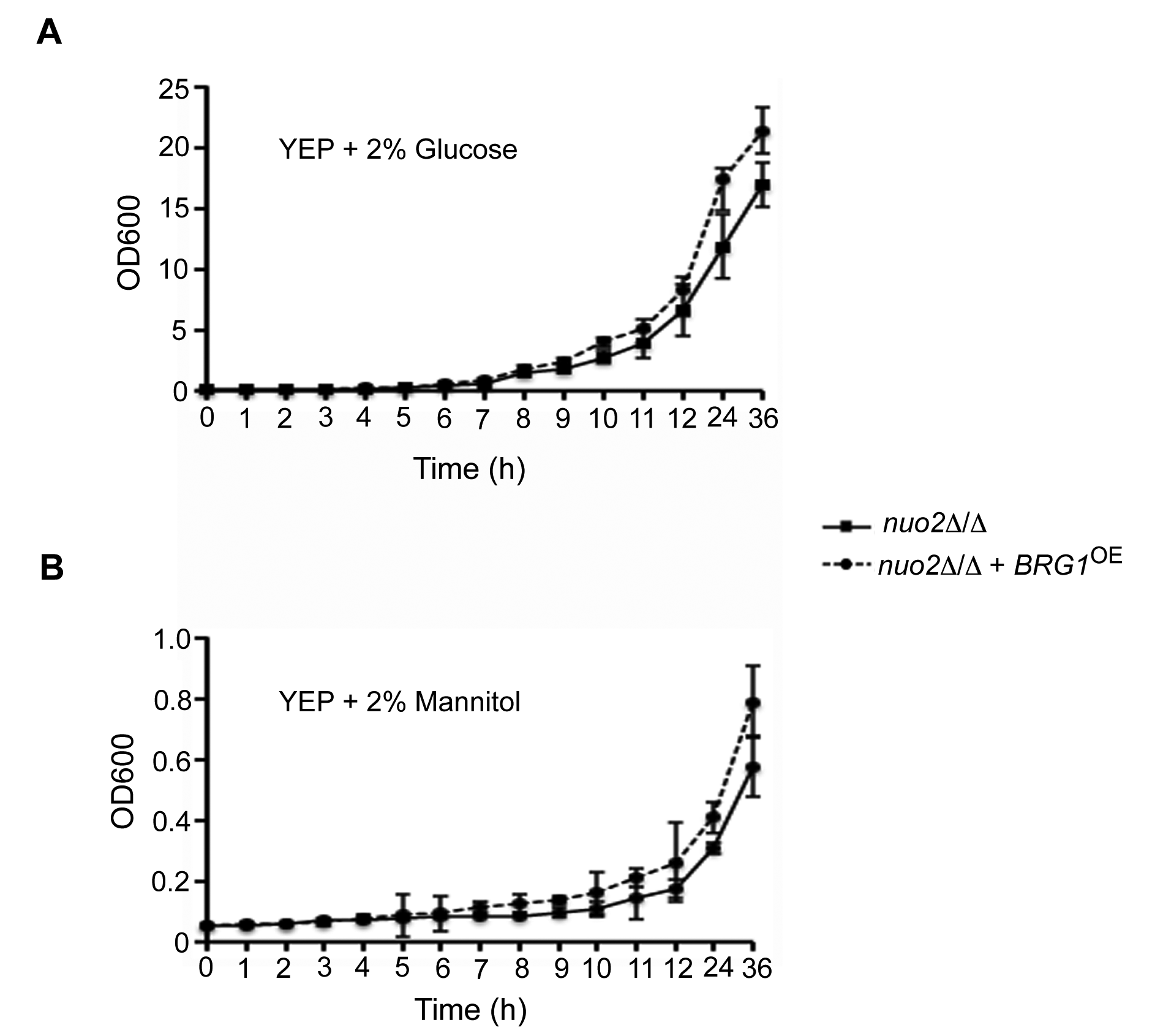

Supplement: S15 Fig — Cells from the nuo2Δ/Δ or BRG1OE (BRG1OE + nuo2Δ/Δ) strain were sub-cultured to OD600 ~0.05 and continued to grow at 30°C for 36h. Growth curves of indicated strains were performed in YEP medium supplemented with 2% glucose (A) or 2% mannitol (B). Growth of each strain was monitored by OD600 measurements over a 36-h time course. The data shown are the average of two experiments done in duplicate. Note that y axis scales of A and B are different. (TIF) [file ppat.1006414.s016.tif]

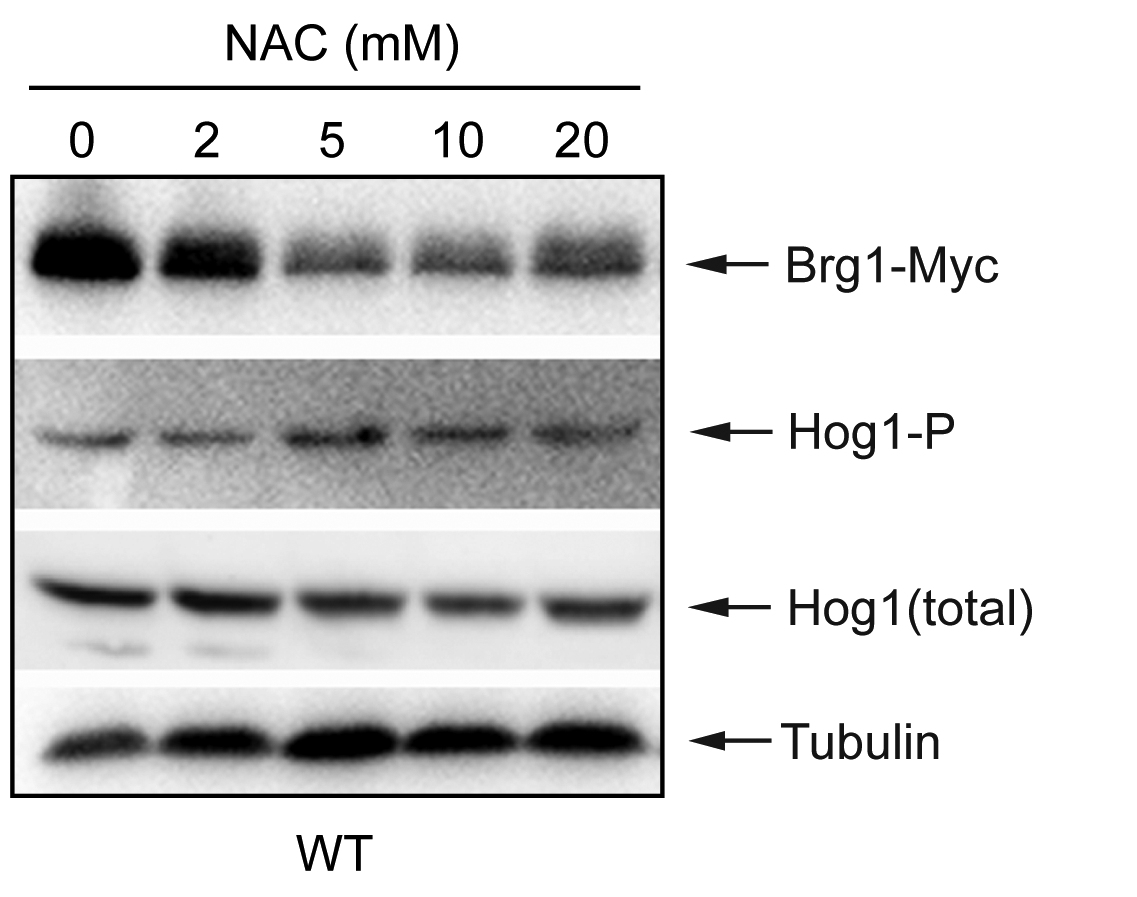

Supplement: S16 Fig — Similar to Fig 5G, the wild type cells grown in mannitol-containing YEP were treated with different doses of the antioxidant N-Acetyl Cysteine (NAC) or were not treated. Samples were taken after 5 hours incubation at 37°C and protein extracts were assayed by sequential immunoblotting with antibodies against Myc epitope, phosphorylated Hog1, total Hog1 and α-tubulin (loading control). (TIF) [file ppat.1006414.s017.tif]

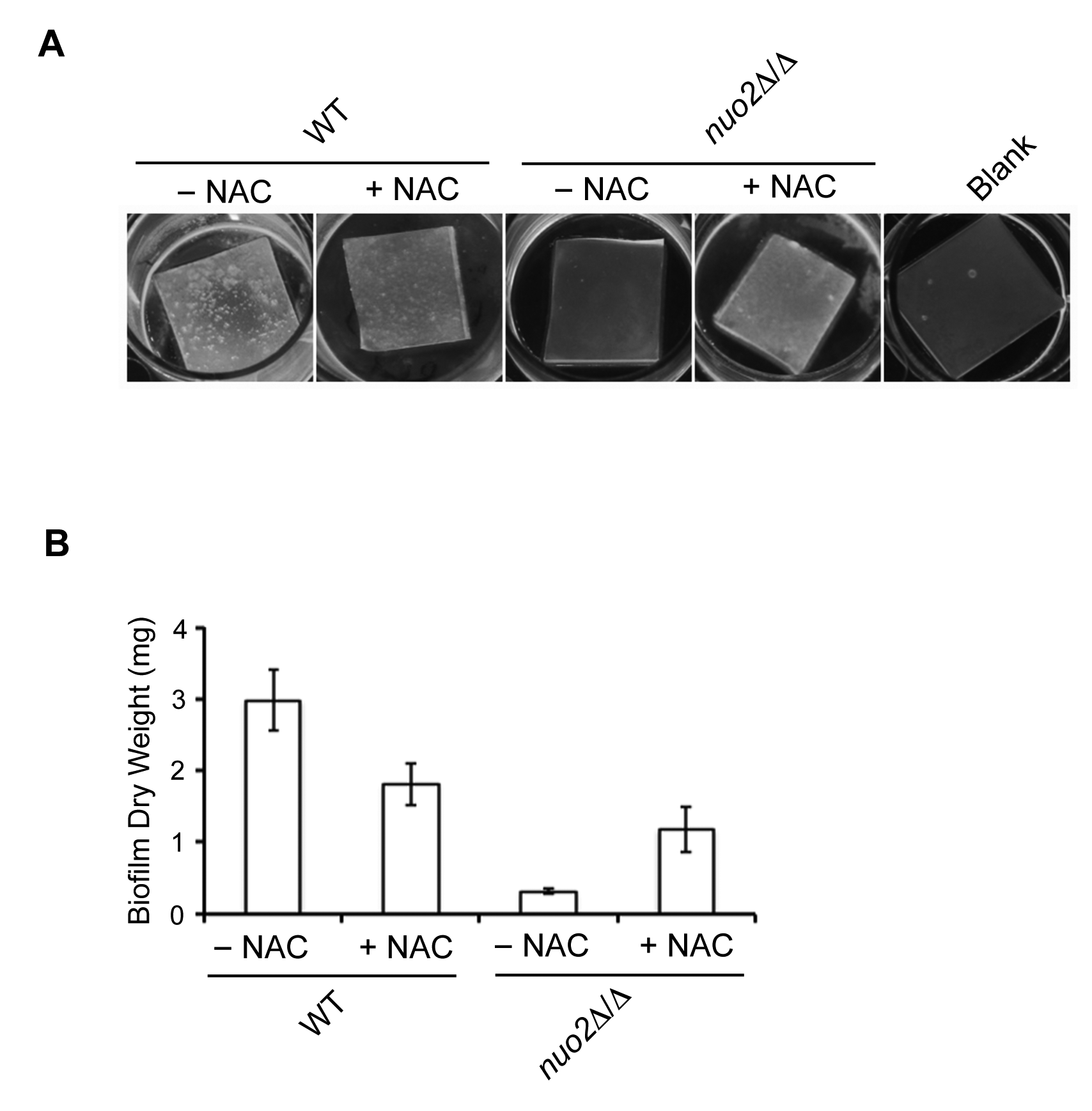

Supplement: S17 Fig — Cells from wild type or nuo2Δ/Δ were grown as biofilms in mannitol-containing Spider medium in the presence or absence of 20mM NAC. Samples were analyzed by (A) cell adhesion on plate with silicone square and (B) biofilm dry weights. Values are the mean ± SD from two independent experiments with at least three replicates. (TIF) [file ppat.1006414.s018.tif]

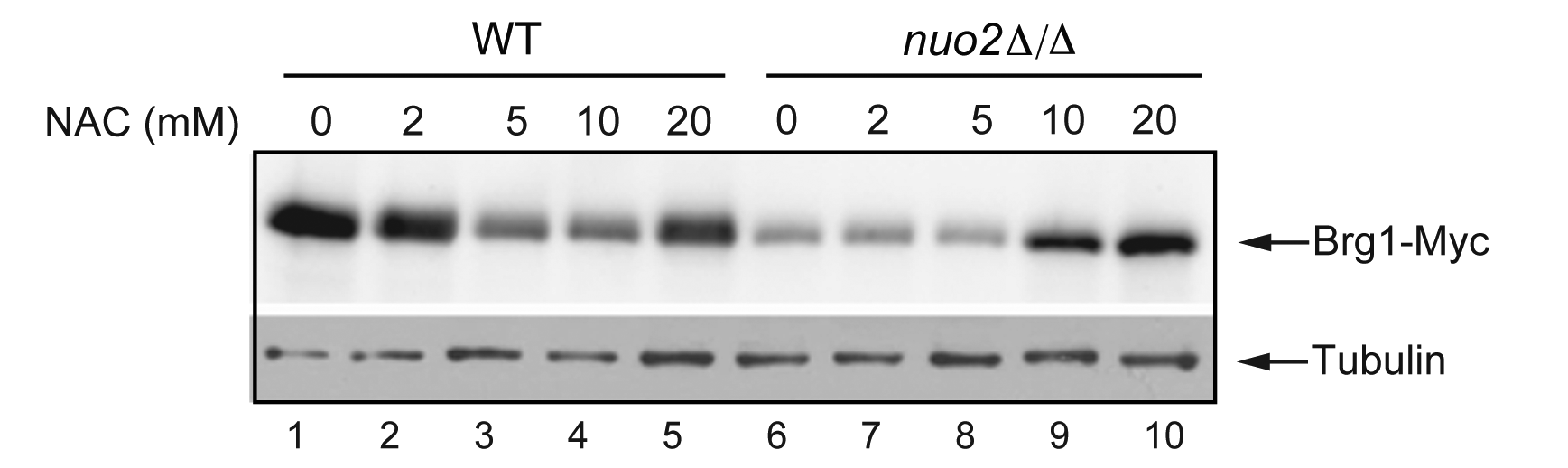

Supplement: S18 Fig — As in S16 Fig, log-phase of wild type or nuo2Δ/Δ cells grown in mannitol-containing YEP medium were treated with different doses of NAC or were not treated. Samples were taken after 5 hours incubation at 37°C and protein extracts were assayed by immunoblotting with antibodies against Myc epitope. Immunoblotting with antibodies against the α-tubulin was used to control for variation in loading. (TIF) [file ppat.1006414.s019.tif]

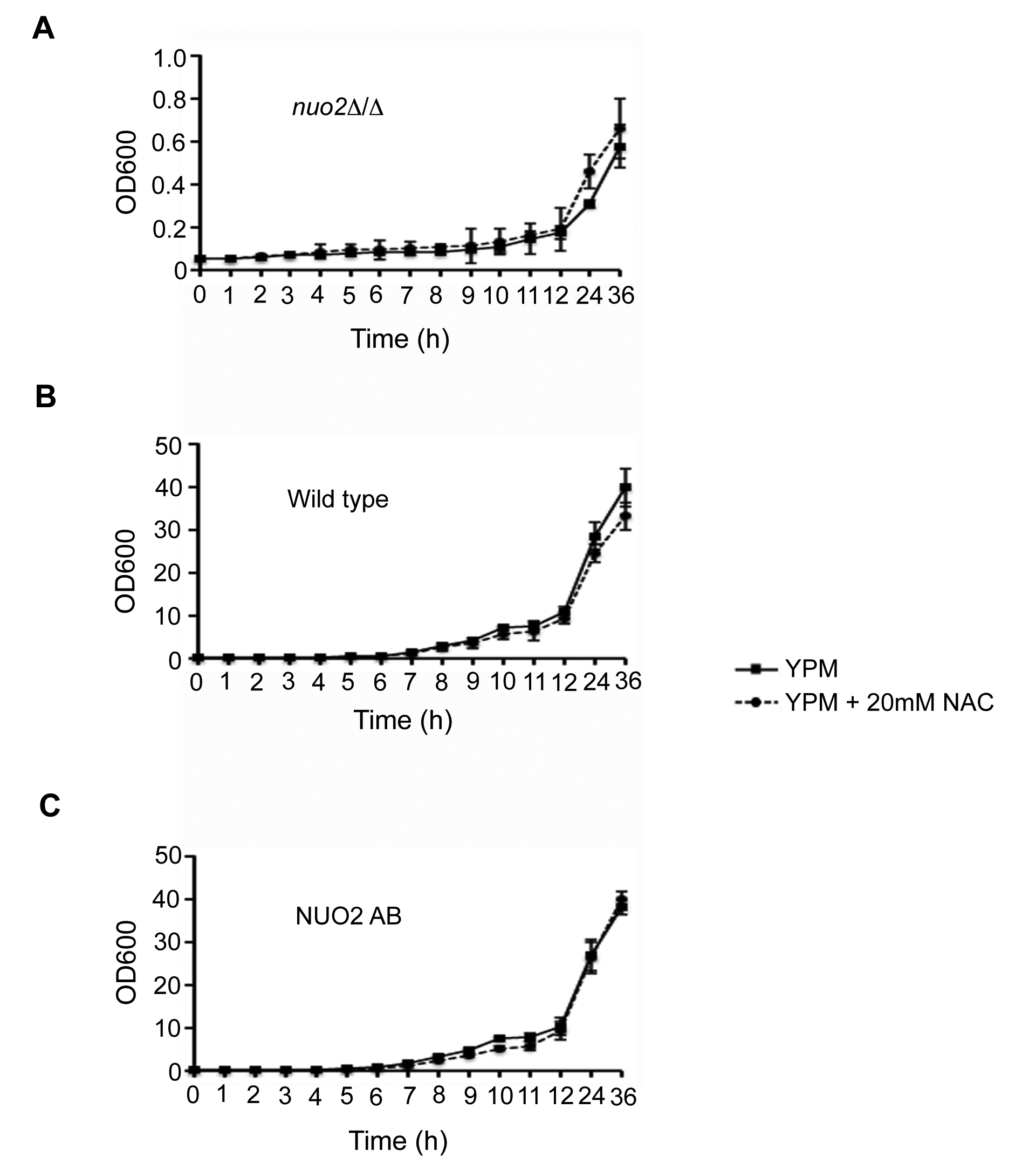

Supplement: S19 Fig — Cells from nuo2Δ/Δ (A), wild type (B) or NUO2 AB (C) were sub-cultured to OD600 ~0.05 and continued to grow at 30°C for 36h. Growth curves were performed in mannitol-containing YEP medium in the presence or absence of 20mM NAC. Growth of each strain was monitored by OD600 measurements over a 36-h time course. The data shown are the average of two experiments done in duplicate. Note that the y axis scale of each figure may be different. (TIF) [file ppat.1006414.s020.tif]

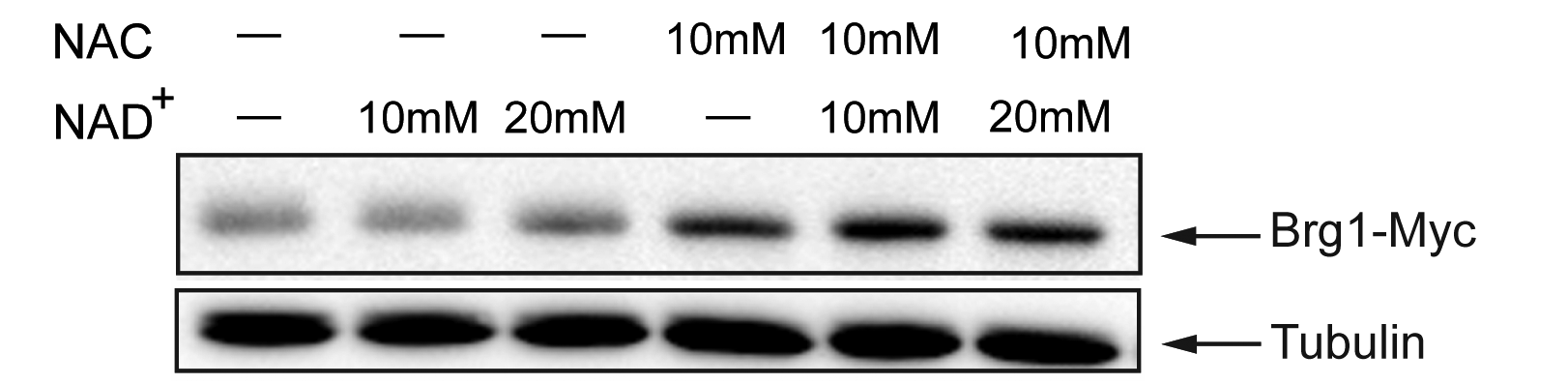

Supplement: S20 Fig — Log-phase of nuo2Δ/Δ mutant cells were treated with NAC (10mM), NAD+ (10mM or 20mM), or both and protein extracts were analyzed by immunoblotting with antibodies against Myc epitope and α-tubulin (loading control). (TIF) [file ppat.1006414.s021.tif]

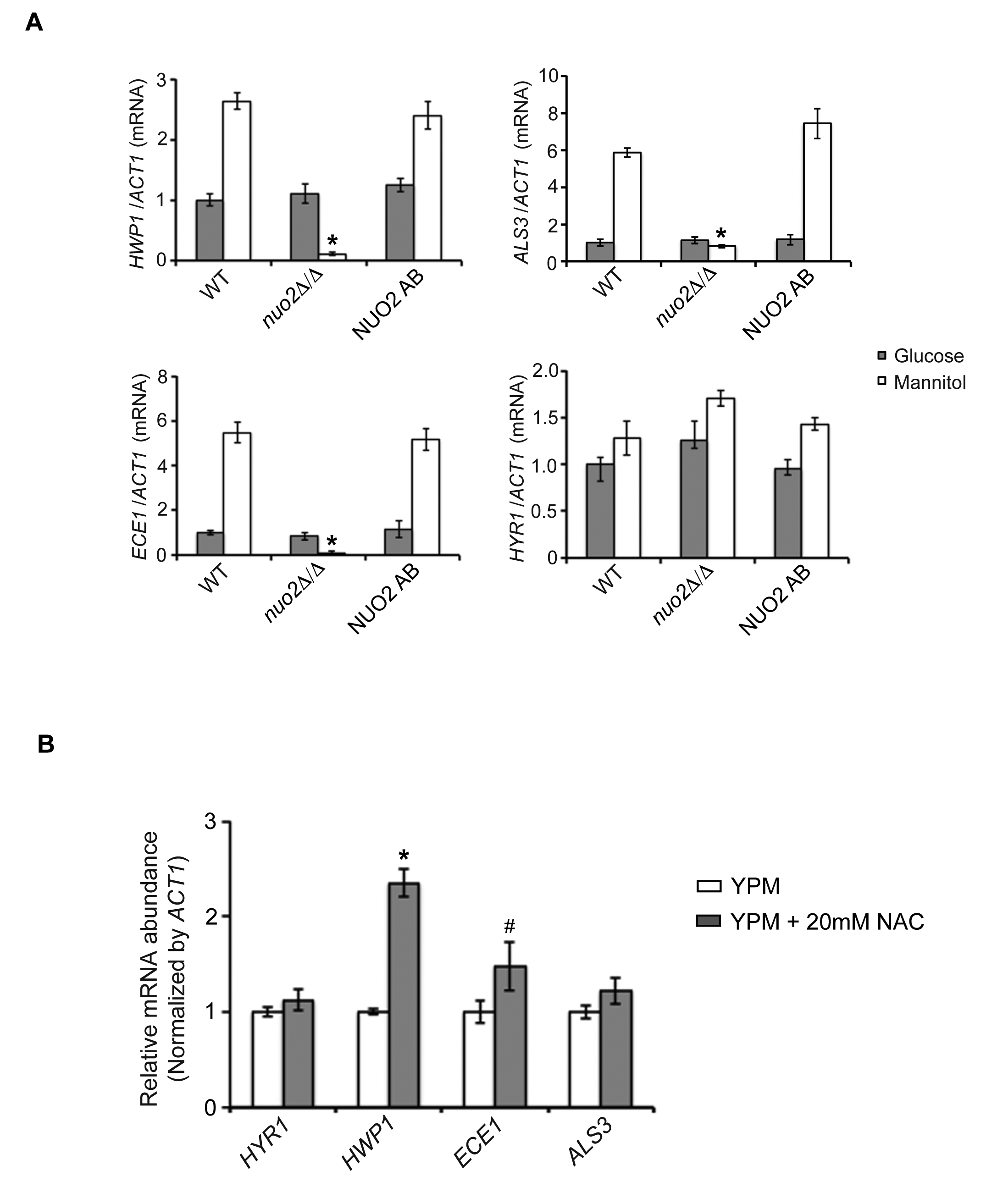

Supplement: S21 Fig — (A) Cells from wild type, nuo2Δ/Δ and NUO2 AB strains were grown on YPD or YPM medium to mid-log stage. Relative transcript levels of four HSGs including HWP1, ALS3, ECE1 and HYR1 were assessed by quantitative PCR (qPCR). Values obtained for each gene were normalized against ACT1 for each sample to give relative expression. “*”represents P<0.001 for WT vs. mutant. Error bars represent standard deviation of three independent biological replicates. (B) An overnight cultures of nuo2Δ/Δ cells was diluted and inoculated in mannitol-containing YEP medium in the presence or absence of NAC (20mM), and continuted to incubate at 37°C for 6-8h. Transcript levels of the same set of genes mentioned above were analyzed by RT-qPCR. (TIF) [file ppat.1006414.s022.tif]

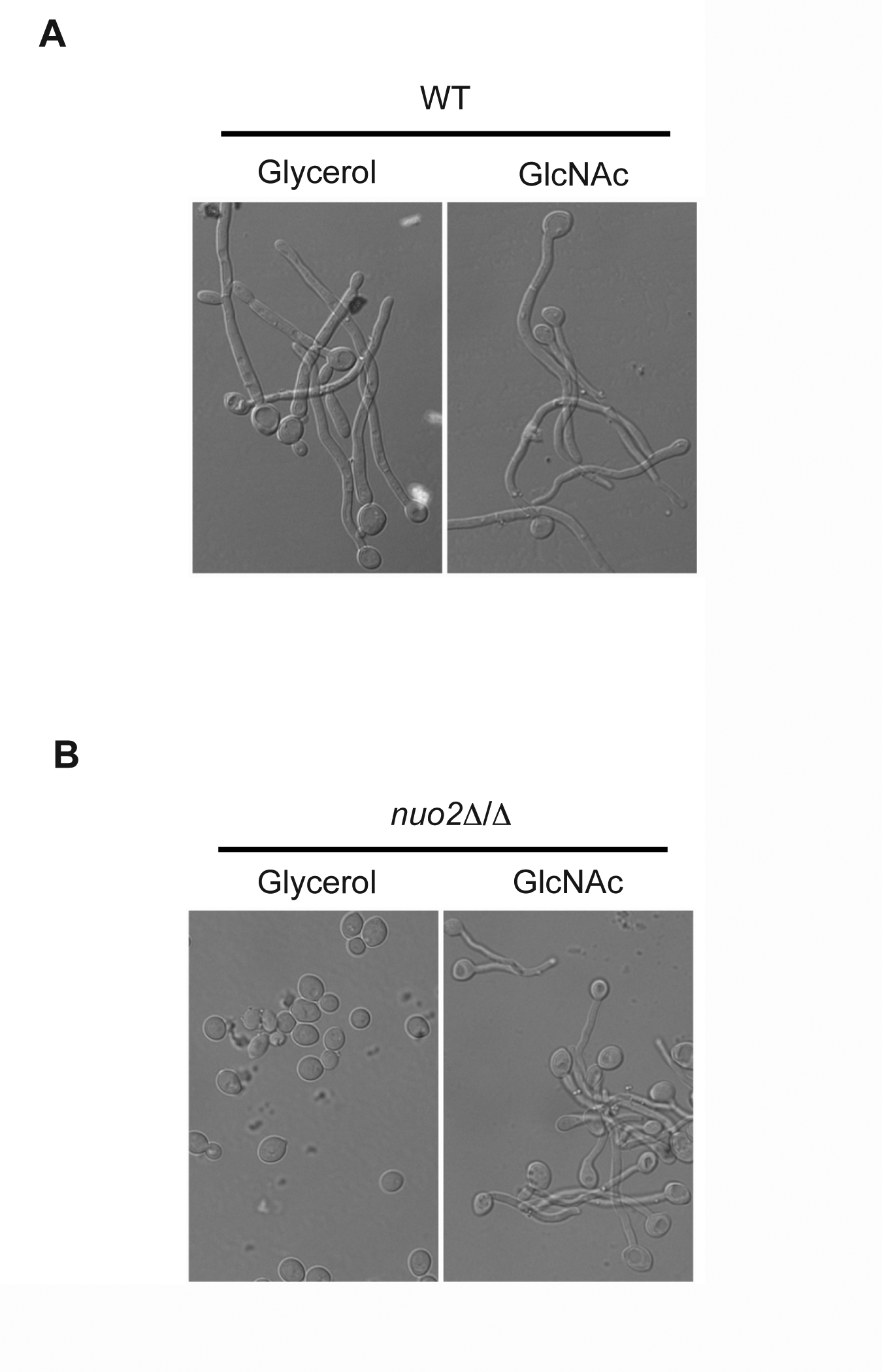

Supplement: S22 Fig — Log-phase cells of WT (A) and nuo2Δ/Δ (B) strains, originally grown in YPD medium, were collected, washed and re-inoculated to equal volume of YEP medium supplemented with 2% of glycerol or GlcNAc. Cells were continued to grow at 37°C for 4h and cell morphologies were visualized under microscopy. (TIF) [file ppat.1006414.s023.tif]

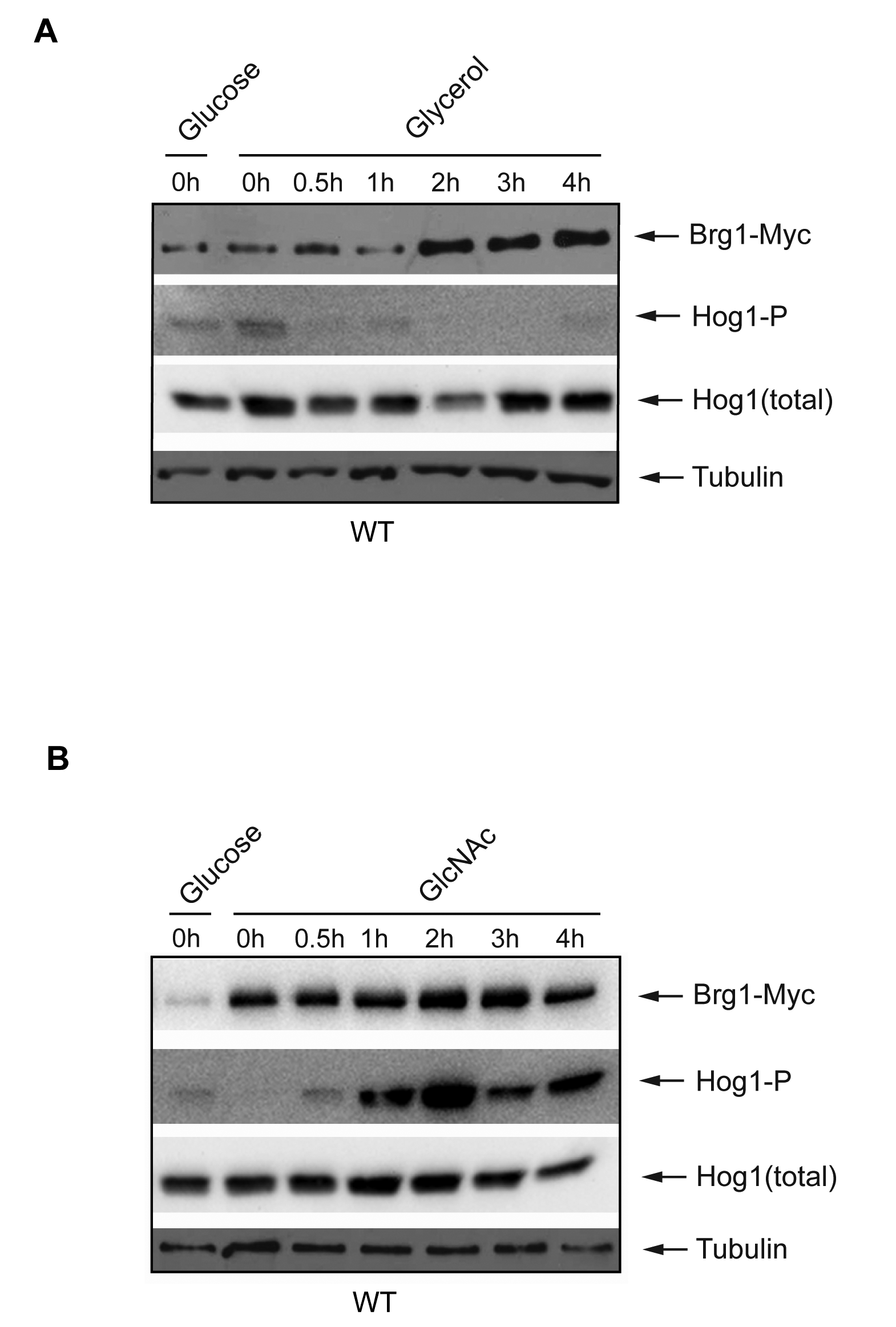

Supplement: S23 Fig — (A) Treating the wild type cells with glycerol induced robust Brg1 expression while the level of phosphorylated Hog1 was almost undetected. As in Fig 6C, log-phase of wild type cells, orginally grown in YPD, were collected, washed and re-inoculated to equal volume of YEP medium supplemented with 2% of glycerol. Cells were continued to grow at 37°C and collected at indicated time points for Western analysis, using antibodies against Myc epitope, phosphorylated Hog1, total Hog1 and α-tubulin (loading control) (B) GlcNAc treatment increased Brg1 expression and Hog1 phosphorylation in wild type C. albicans. Cells were treated exactly the same as in (A), with the exception that 1% of GlcNAc were used. (TIF) [file ppat.1006414.s024.tif]

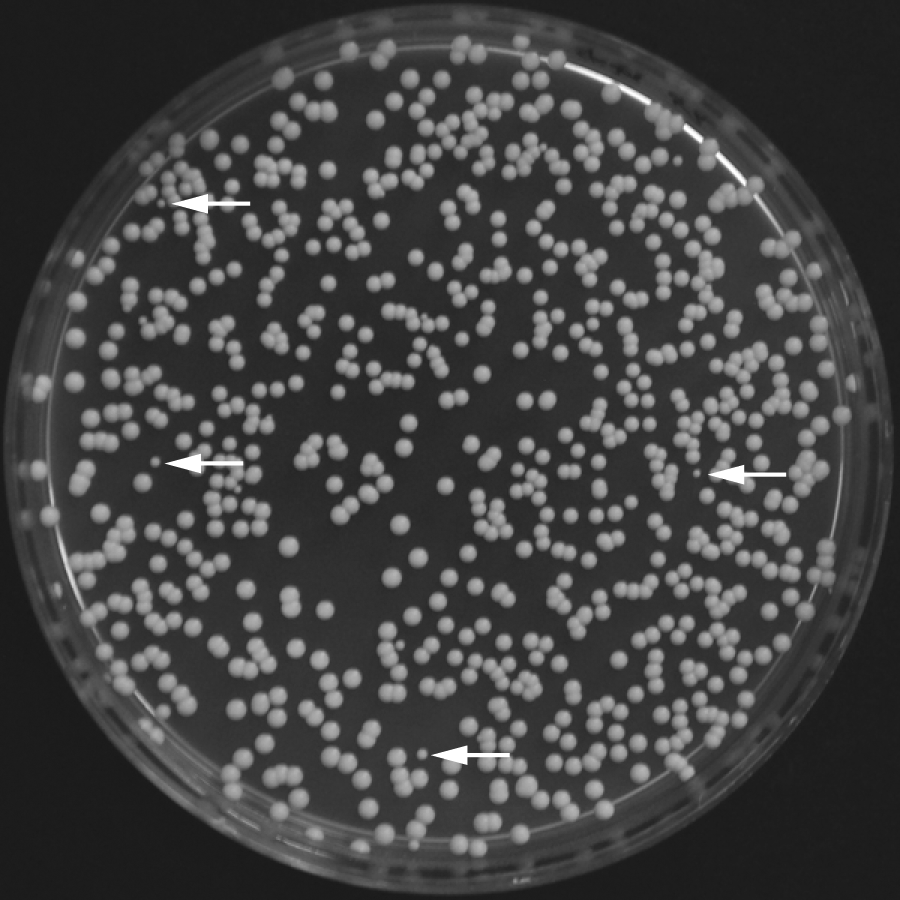

Supplement: S24 Fig — 6–8 week female BLAB/c mice were infected by oral gavage with 108 CFUs of a 1:1 mixtures wild type (SN250) and nuo2Δ/Δ mutant. Fecal pellets were collected at specified intervals and homogenates of mouse feces were plated onto Sabouraud agar medium (with ampicillin 50 μm/ml, gentamicin 15 μm/ml). Shown is a representative image of mixed C. albicans cells isolated from mouse fecal samples at day 5 after infection. Small colonies (typified with arrows) were confirmed by PCR to be derived from nuo2Δ/Δ mutant. (TIF) [file ppat.1006414.s025.tif]

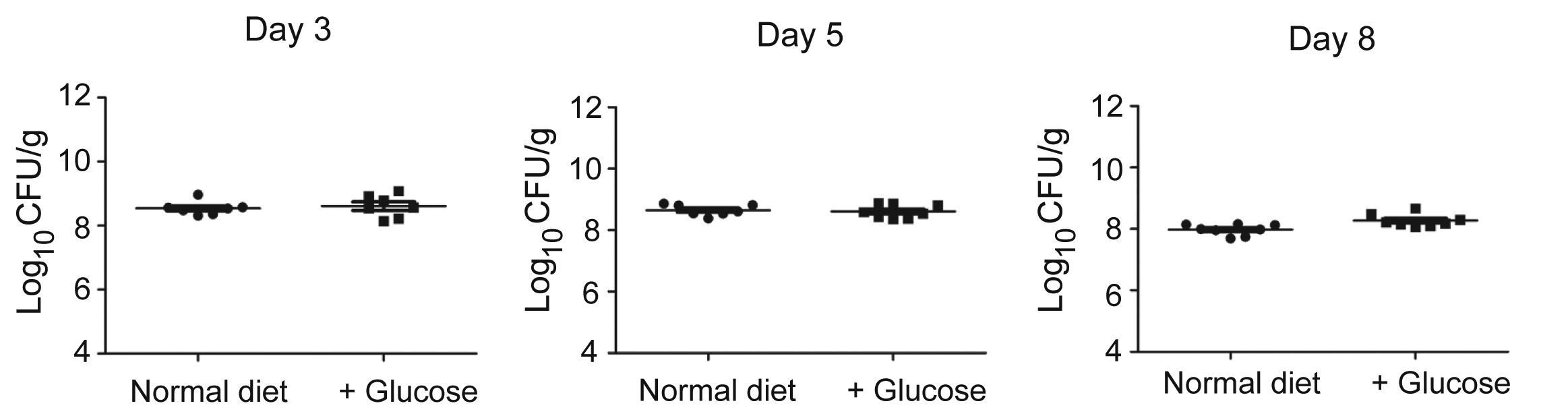

Supplement: S25 Fig — As in Fig 6F, a similar commensalism experiment were conducted. All manipulations were the same with the exception that the wild type C. albicans strain was used. (TIF) [file ppat.1006414.s026.tif]
